# Impact of COVID-19, lockdowns and vaccination on immune responses in a HIV cohort in the Netherlands

**DOI:** 10.3389/fimmu.2024.1459593

**Published:** 2024-12-18

**Authors:** Twan Otten, Xun Jiang, Manoj Kumar Gupta, Nadira Vadaq, Maartje Cleophas-Jacobs, Jéssica C. dos Santos, Albert Groenendijk, Wilhelm Vos, Louise E. van Eekeren, Marc J. T. Blaauw, Elise M.G. Meeder, Olivier Richel, Vasiliki Matzaraki, Jan van Lunzen, Leo A. B. Joosten, Yang Li, Cheng-Jian Xu, Andre van der Ven, Mihai G. Netea

**Affiliations:** ^1^ Department of Internal Medicine and Radboud Center for Infectious Diseases, Radboud University Medical Center, Nijmegen, Netherlands; ^2^ Department of Internal Medicine and Infectious Diseases, Elizabeth-Tweesteden Ziekenhuis, Tilburg, Netherlands; ^3^ Centre for Individualised Infection Medicine (CiiM), a joint venture between the Helmholtz-Centre for Infection Research (HZI) and the Hannover Medical School (MHH), Hannover, Germany; ^4^ TWINCORE, a joint venture between the Helmholtz-Centre for Infection Research (HZI) and the Hannover Medical School (MHH), Hannover, Germany; ^5^ Department of Internal Medicine and Department of Medical Microbiology and Infectious diseases, Erasmus Medical Center (MC), Erasmus University, Rotterdam, Netherlands; ^6^ Department of Internal Medicine and Infectious Diseases, OLVG, Amsterdam, Netherlands; ^7^ Department of Psychiatry, Radboudumc, Radboud University, Nijmegen, Netherlands; ^8^ Donders Institute for Brain, Cognition and Behavior, Radboud University, Nijmegen, Netherlands; ^9^ Nijmegen Institute for Scientist-Practitioners in Addiction (NISPA), Radboud University, Nijmegen, Netherlands; ^10^ Translational Medical Research, ViiV Healthcare, Brentford, United Kingdom; ^11^ Department of Medical Genetics, Iuliu Hatieganu University of Medicine and Pharmacy, Cluj-Napoca, Romania; ^12^ Department of Immunology and Metabolism, Life and Medical Sciences Institute, University of Bonn, Bonn, Germany

**Keywords:** COVID-19, lockdown, vaccination, inflammation, hygiene hypothesis

## Abstract

**Introduction:**

During the COVID-19 pandemic, major events with immune-modulating effects at population-level included COVID-19 infection, lockdowns, and mass vaccinations campaigns. As immune responses influence many immune-mediated diseases, population scale immunological changes may have broad consequences.

**Methods:**

We investigated the impact of lockdowns, COVID-19 infection and vaccinations on immune responses in the 2000HIV study including 1895 asymptomatic virally-suppressed people living with HIV recruited between October 2019 and October 2021. Their inflammatory profile was assessed by targeted plasma proteomics, immune responsiveness by cytokine production capacity of circulating immune cells, and epigenetic profile by genome-wide DNA methylation of immune cells.

**Results:**

Past mild COVID-19 infection had limited long-term immune effects. In contrast, COVID-19 vaccines and especially lockdowns significantly altered both the epigenetic profile in immune cells at DNA methylation level and immune responses. Lockdowns resulted in a strong overall exaggerated immune responsiveness, while COVID-19 vaccines moderately dampened immune responses. Lockdown-associated immune responsiveness alterations were confirmed in 30 healthy volunteers from the 200FG cohort that, like the 2000HIV study, is part of the Human Functional Genomics Project.

**Discussion:**

Our data suggest that lockdowns have unforeseen immunological effects. Furthermore, COVID-19 vaccines have immunological effects beyond anti-SARS-CoV-2 activity, and studies of their impact on non-COVID-19 immune-mediated pathology are warranted.

## Introduction

The emergence of the novel coronavirus disease 2019 (COVID-19), caused by the severe acute respiratory syndrome coronavirus 2 (SARS-CoV-2) (1, 2), was first reported in late December 2019. Since then, the virus spread throughout the world becoming a major pandemic at the beginning of 2020, with severe consequences for the health of millions of individuals. As human populations lacked specific immunity against this novel infection, despite some limited cross-protection by previous exposure to other members of the coronavirus family (3), this led initially to very high absolute levels of morbidity and mortality. Therefore, combinations of urgent measures were taken by various countries: measures of limiting social contact to slow the spread of the infection (lockdowns, but also social distancing and masks), urgently increasing hospital capacity, drug repurposing to treat the disease, and massive investment in the development of new COVID-19 vaccines. Over the following three years, these measures were successful in mitigating the spread of the infection, while at the same time a slow but consistent increase of immunity at population level developed as a result of exposure to the virus or the newly developed vaccines (4). New anti-viral medications and repurposed immunotherapies improved outcome of infected individuals (5). Since the beginning of 2023 the infection has entered a new endemic phase, and it is believed that COVID-19 will remain part of the spectrum of common human infections, especially during the winter season.

During the three years of the pandemic phase of the infection, the general use of public health measures to modify infectious pressure in the population (lockdowns, social distancing, masks) or to directly increase anti-viral immune responses (vaccines) were the most important features of the strategy to control the spread of the virus. However, these measures can potentially also modulate immune responses at population level beyond the direct interaction with SARS-CoV-2 alone. In addition, COVID-19 infection itself exerts long-term immune effects (6) that, although primarily aimed at limiting the impact of the virus itself, can also have heterologous non-specific consequences. Immune responses do not only determine the outcome of infections, but are also crucial for the pathophysiology of many other diseases including inflammatory and autoimmune diseases, cancer, allergic disease, and neurodegenerative diseases (7). Therefore, it is important to assess the potential immune-modulatory effects of COVID-19 infection, lockdowns and vaccination campaigns at population level. This would allow us to assess their potential impact on various pathologies, as well as to draw lessons for the use of such measures for future pandemic preparedness.

## Results

### Human cohorts

**Figure 1:**
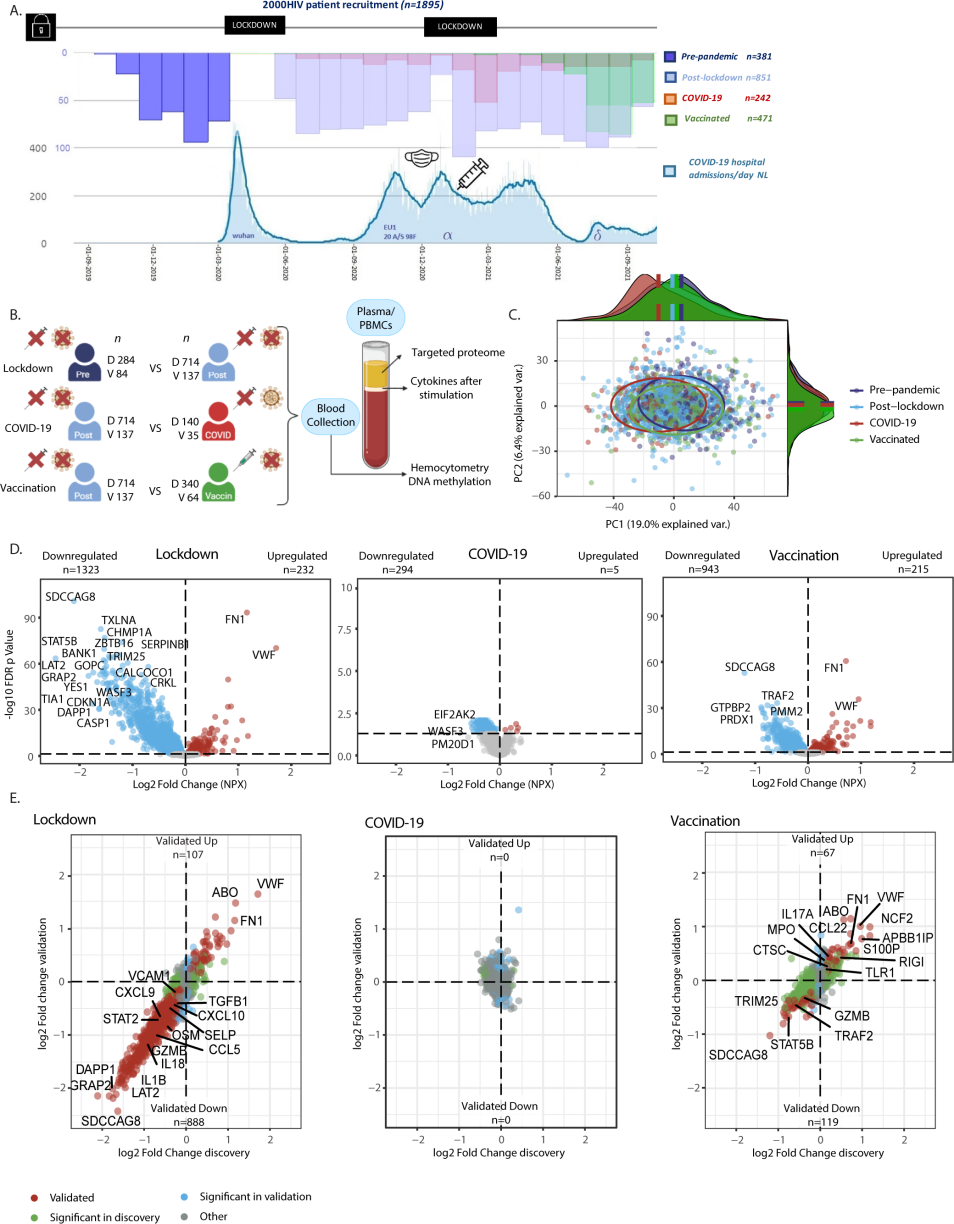
Study methods and influence of the pandemic on plasma proteome in people living with HIV (PLWHIV). **(A)** Timeline of Participant Recruitment and COVID-19 Pandemic in the Netherlands. Upper Histogram: Study Recruitment Timeline. Bottom Graph: COVID-19 Hospitalizations and Dominant Circulating Strain*. X-axis: Date. Y-axis: Number of Patients (Different scales per graph). Mask: Introduction of Mask Obligation. Vaccin: Introduction of Vaccination Campaign. Lockdown: Period of Lockdowns. **(B)** Methods Overview. PLWHIV were enrolled into separate discovery and validation cohorts. The effects of the following groups were compared: -Social Isolation (referred to as Lockdown): Unvaccinated COVID-19 negative PLHIV before (Pre-pandemic) vs after the first lockdown (Post-lockdown; n discovery 284 vs 714, validation 84 vs 137, respectively). -COVID-19: Unvaccinated PLWH with vs without past COVID-19, included after the first lockdown (n discovery 140 vs 714, validation 35 vs 137, respectively). -COVID-19 Vaccination (referred to as Vaccination): PLWH with vs without COVID-19 vaccination, enrolled after the first lockdown, excluding those with past COVID-19 infection (n discovery 340 vs 714, validation 64 vs 137, respectively). Blood was collected during participant visits, and results from hemocytometry, targeted plasma proteomics, ex-vivo PBMC stimulation experiments, and DNA methylation comparing these groups are shown. **(C)** Principal Component Analysis (PCA) of Protein Levels from the Discovery Cohort. PCA on residuals after adjusting for sex and age showing distinct proteomic profiles of study groups based on PC1 and PC2. Ellipses were centered around the median of the PCs; On PC1 all groups showed statistically significant differences (adj. p <0.05; Wilcoxon’s). On PC2 the vaccinated vs. pre-pandemic, post-lockdown, and COVID-19 group showed statistically significant differences. **(D)** Volcano Plots Showing Differential Abundance of Proteins in the Discovery Cohort. X-axis: Log2 Fold Change of Normalized Protein Abundance (NXP). Y-axis: -Log10 Benjamin-Hochberg False Discovery Rate (FDR) adjusted p-value. Colored dots represent FDR adj. p < 0.05. Red dots indicate upregulated proteins, while blue dots represent downregulated proteins. Please note the different Y-axis range in COVID-19 plot. Results from linear models adjusted for age, sex, and seasonality. Labeled are the most significantly differentially abundant proteins (DAPs). **(E)** Four Quadrant Scatter Plots Showing Log2 Fold Change in Normalized Protein Abundance (NXP) in the Discovery Cohort on the X-axis and Validation Cohort on the Y-axis. Green dots represent proteins significant only in the discovery cohort (FDR adj. p < 0.05). Blue dots indicate proteins significant only in the validation cohort (p < 0.05). Red dots indicate proteins significant in the same direction in both cohorts (=validated). Labeled are specific DAPs involved in (systemic) inflammation, as well as the one with the largest effect size. Pre, Pre-pandemic; Post, Post-lockdown; COVID, COVID-19; Vaccin, Vaccinated; D, Discovery cohort; V, Validation cohort; *n*, number of participants. * derived from publicly available data from the RIVM and Dutch government at https://coronadashboard.government.nl/landelijk/ziekenhuis-opnames.

In the present study, we investigated the impact of lockdowns, SARS-CoV-2 infection and COVID-19 vaccination on the immune responses at populations level in the 2000HIV study, which is part of the Human Functional Genomics Project (8). The 2000HIV study is a large observational study that assesses factors that impact immune responses and comorbidities in 1895 people living with HIV (PLHIV) in the Netherlands who are virologically suppressed on combination antiretroviral therapy (cART) for more than six months and free of any acute conditions (9). The inclusion of the 2000HIV study participants started in October 2019, before the onset of the pandemic, and continued till October 2021. Thus, we were able to recruit a large number of PLHIV at all stages of the pandemic during which different public health interventions were implemented. The PLHIV were enrolled into an independent discovery cohort (n=1559) and a validation cohort (n=336), divided based on the specialized HIV treatment center that recruited the participants. Patients who had both a positive coronavirus status and were vaccinated (n=63), had no covid serology measured (n=7), had positive COVID-19 serology before the pandemic (n=7) or were on immunosuppressants (n=20) were excluded from our analysis. Subsequently, both the discovery and validation cohort were further stratified into: pre-pandemic (368 individuals recruited before the start of the pandemic), post-lockdown (851 individuals recruited after the imposition of lockdowns in the Netherlands, who did not contract COVID-19 and were not vaccinated), COVID-19 infection (175 individuals who were infected in the period before blood sampling as defined by positive PCR or serology test), and COVID-19 vaccinated groups (404 individuals vaccinated against COVID-19) (**Figures 1A, B**).

Results from hemocytometric analysis, targeted plasma proteomics, DNA methylation and ex-vivo stimulation experiments were first compared between these groups in the 2000HIV study (**Figure 1B**). While the clinical characteristics were generally similar between the groups within the discovery and validation cohorts (e.g. alcohol/recreational drug use, BMI, age, latest CD4 count which were generally in normal range and years on ART), patients included before the pandemic were more frequently male and of European ancestry: subsequently, when appropriate, we corrected for these variables in the analyses. Furthermore, the median duration between most recent COVID-19 infection or COVID-19 vaccination and blood drawing was 117 (IQR 40-177) and 49 days (IQR 20-71) in the discovery cohort, and 74 (IQR 35-140) and 21 days (IQR 11-34) in the validation cohort, respectively. As a consequence of the study timelines and vaccination patterns, participants in the post-lockdown group were recruited sooner after implementation of the most recent lockdown as compared to COVID-19 vaccinated participants (median 91 versus 232 days in the discovery cohort respectively, p<0.0001; **Table 1**).

**Table 1:** Baseline characteristics of the 2000HIV cohort.

Discovery	pre-pandemic	post-lockdown	p value*	COVID-19	p value**	vaccinated	p value***
n	284	714	-	140	-	340	-
ethnic-white	85%	74%	0.0002	77%	0.026	67%	0.023
female	7%	16%	0.0001	24%	0.0003	17%	0.47
age [years]	54(44-60)	53(43-59)	0.52	49(38-56)	<0.0001	53(44-60)	0.81
smoking	25%	31%	0.013	31%	0.92	32%	0.56
VL > 50	2%	3%	0.68	3%	1.0	3%	1.0
CD4 latest [x10^6^ cells/L]	710 (510-920)	735(570-928)	0.074	700(550-910)	0.32	683(503-924)	0.030

Validation	pre-pandemic	post-lockdown	p value*	COVID-19	p value**	vaccinated	p value***
n	84	137		35		64	
ethnic-white	83%	87%	0.56	86%	0.79	88%	0.41
female	12%	18%	0.34	20%	0.81	13%	1.0
age [years]	52(47-59)	52(45-59)	0,8	51(44-58)	0.68	58(51-64)	0.0008
smoking	24%	34%	0.033	34%	1.0	28%	0.25
VL > 50	5%	4%	0.73	6%	0.63	3%	1.0
CD4 latest [x10^6^ cells/L]	605(465-760)	650(450-810)	0.47	660(445-790)	0.96	715(545-870)	0.14

Statistical testing: Categorial variables: proportions are shown and difference tested with Fisher's exact test. Continuous variables: Median with (quartile 1 - quartile 3) are shown and difference tested with Mann-Whitney U test.

*p value comparing the pre-pandemic group to the post lockdown group.

**p value comparing the COVID-19 group to the post lockdown group.

***p value comparing the vaccinated group to the post lockdown group.

In addition, healthy volunteers were assessed during and after the pandemic in a second independent cohort named 200FG, that is also part of the Human Functional Genomics Project. In the 200FG study, approximately 200 healthy volunteers donate blood yearly for the assessment of their immune response profile (10, 11). In 36 out of the 101 volunteers, in which samples from both 2020 and 2022 were available, the cytokine production upon stimulation of peripheral blood mononuclear cells (PBMCs) with microbial and non-microbial stimuli was assessed (**Supplementary Table S1**).

### Distinct proteomic profiles before and during the COVID-19 pandemic in PLHIV

First, we assessed the overall inflammatory status of the 2000HIV participants by analyzing their plasma proteomic profiles. A total of 3072 proteins were measured using proximity extension assay technology (Olink^®^) and, after applying quality controls, 2367 proteins were included for statistical analysis. Principal component analysis (PCA) was performed, adjusting for sex and age (**Figure 1C**), revealing significant shifts between all groups (**Supplementary Table S2**). Specifically, the results of Wilcoxon’s rank-sum test showed significant differences in principal component (PC)1 between the pre-pandemic versus post-lockdown group (p <0.0001), the post-lockdown versus COVID-19 group (p <0.0001), and between the post-lockdown and vaccinated group (p =0.029). Additionally, in PC2, the vaccinated group differed significantly from the pre-pandemic (p <0.0001) group, the post-lockdown group (p =0.025), and the COVID-19 group (p =0.025), indicating distinct proteomic profiles in each group.

Next, we further characterized these differences through differential abundance analysis. In the discovery cohort of the 2000HIV study, we identified 1323 downregulated and 232 upregulated proteins in the post-lockdown group compared to pre-pandemic group, after false discovery rate (FDR) correction and adjustment for the effects of seasonality, sex, and age (**Figure 1D**). Compared with the post-lockdown proteome profile, we identified 294 downregulated and 5 upregulated proteins in the COVID-19 group, and 943 downregulated and 215 upregulated proteins in the COVID-19 vaccinated group (**Figure 1D**). In the validation cohort of the 2000HIV study, we found 925 downregulated and 137 upregulated proteins after lockdown, 23 and 55 proteins down- and up-regulated after COVID-19, and 128 and 128 proteins down- and up-regulated after vaccination (**Supplementary Figure 1A**).

**Figure 2:**
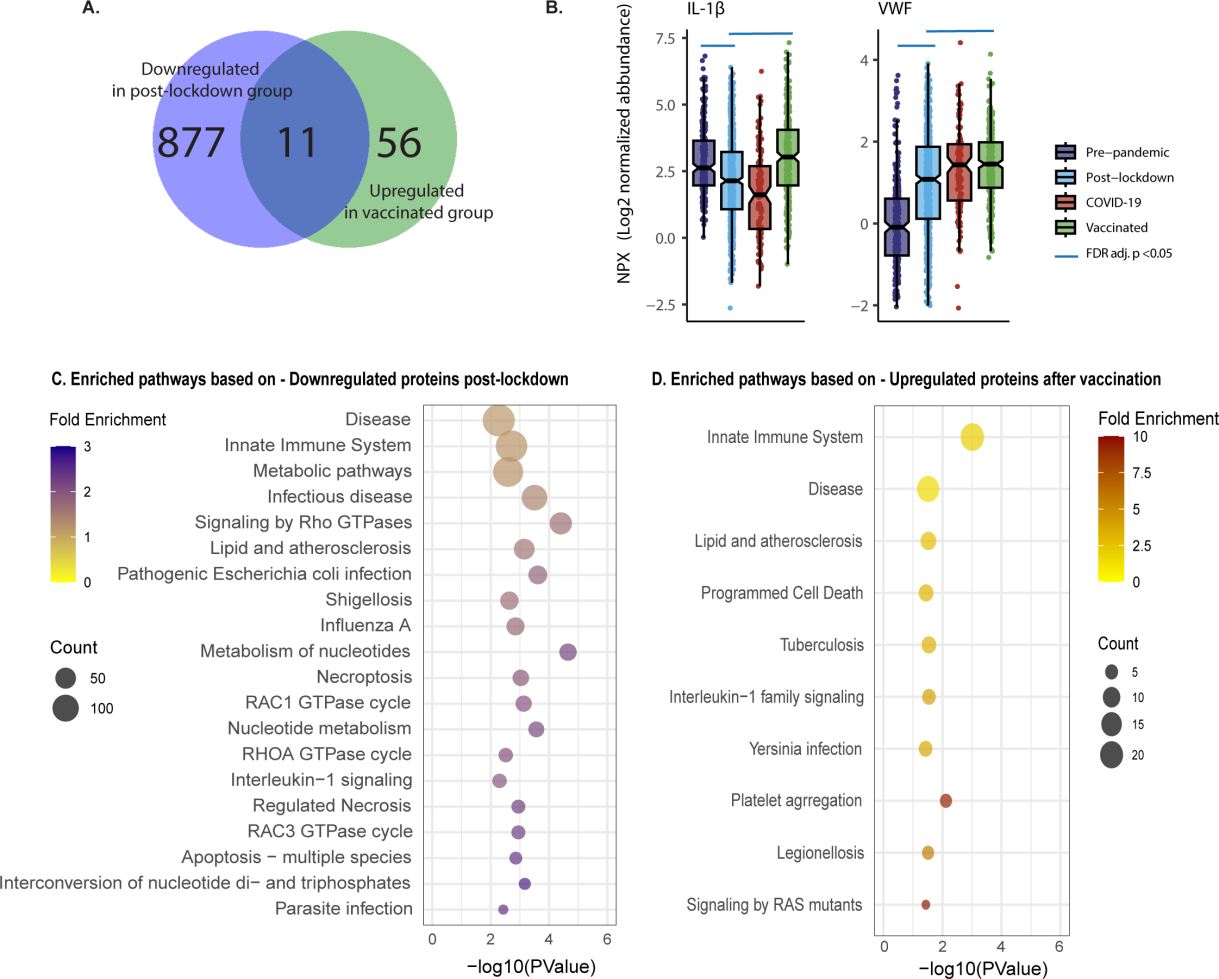
Distinct effects of vaccination and lockdown on plasma proteome in PLHIV. **(A)** Venn Diagram showing only 11 out of the 888 validated differentially abundant proteins (DAPs) that had lower concentration post lockdown, were upregulated after vaccination of the 2000HIV study. **(B)** Box Scatter Plots showing Log2 normalized protein abundance per group in the discovery cohort of the 2000HIV study. Blue line: FDR adj. p value <0.05 result from the differential abundance analysis. **(C, D)**. Top 20 most significant pathway enrichment analysis results based on p value, presented as a bubble plot for Lockdown **(C)**, and Vaccination **(D)**, ordered on count; duplicate pathways were removed.

To demonstrate consistency of directionality and effect size, unaffected by the differences in sample size between the groups, we generated scatter plots with log-fold change in both the discovery and validation cohort of the 2000HIV study, with proteins colored according to significance (significant in both cohorts, significant in only one cohort, or not significant in either). Post-lockdown, 888 proteins were validated as downregulated and 107 as upregulated in both cohorts (**Figure 1E**), with lockdowns showing a profound and coherent effect. COVID-19 infection did not result in any differentially abundant proteins (DAPs) that were validated between the cohorts, and there was no coherent effect in the log-fold change plot. After COVID-19 vaccination, we identified 119 validated downregulated and 67 upregulated proteins, exhibiting lower effect sizes than the effect of lockdowns. Interestingly, looking at the validated DAPs, only 11 out of the 888 proteins that were downregulated after lockdown have been subsequently upregulated after vaccination. This indicates a different/new proteomic profile after vaccination compared to the pre-pandemic status, not a return to the pre-pandemic inflammatory state (**Figure 2A**). An example of a protein down-regulated by lockdown and returning back to normal concentrations after vaccination is IL-1β (**Figure 2B**). In contrast, other proteins such as VWF were increased by lockdown, and further upregulated by vaccination (**Figure 2B**). However, since the COVID-19 vaccinated participants were included at a later timepoint after lockdown initiation than the post-lockdown group, the proteomic changes in the vaccinated group could be attributed to either the vaccination itself or the waning effects of the lockdown. When selecting only patients which were included between 150 and 200 days after lockdown, no DAPs were consistently validated to change after vaccination; however, only 18 unvaccinated participants remained to be included in the validation cohort. When correcting for days since most recent lockdown implementation, there were 17 upregulated proteins after COVID-19 vaccination (**Supplementary Figure 1C**). This significantly lower number compared to the overall number of proteins identified in the entire COVID-19 vaccine group suggests that an important factor driving changes in the proteome in this group compared with post-lockdown individuals is in fact a waning of the lockdown effects on the inflammatory proteome.

In the scatter plots presented in **Figure 1E**, specific DAPs are labeled, showing downregulation of pro-inflammatory cytokines, chemokines, and adhesion factors in the post-lockdown group, indicating reduced systemic inflammation. In contrast, the COVID-19 vaccinated group displayed an overall increased inflammatory profile. Functional enrichment analysis performed on DAPs confirmed that mainly immune-related pathways were downregulated after lockdown (**Figure 2C**, **Supplementary Figure 1D**). On the other hand, the pathways analysis of DAPs showed that circulating proteins involved in anti-microbial defense and inflammation (innate immune system, infectious diseases, IL-1 signaling) were mostly upregulated after COVID-19 vaccination (**Figure 2D**, **Supplementary Figure 1E**). Interestingly, platelet aggregation/integrin signaling was found to be upregulated both after lockdown and vaccination (**Figure 2C**, **Supplementary Figures 1D–H**).

### In PLHIV, innate immune responsiveness increased after lockdown, but decreased after vaccination

**Figure 3:**
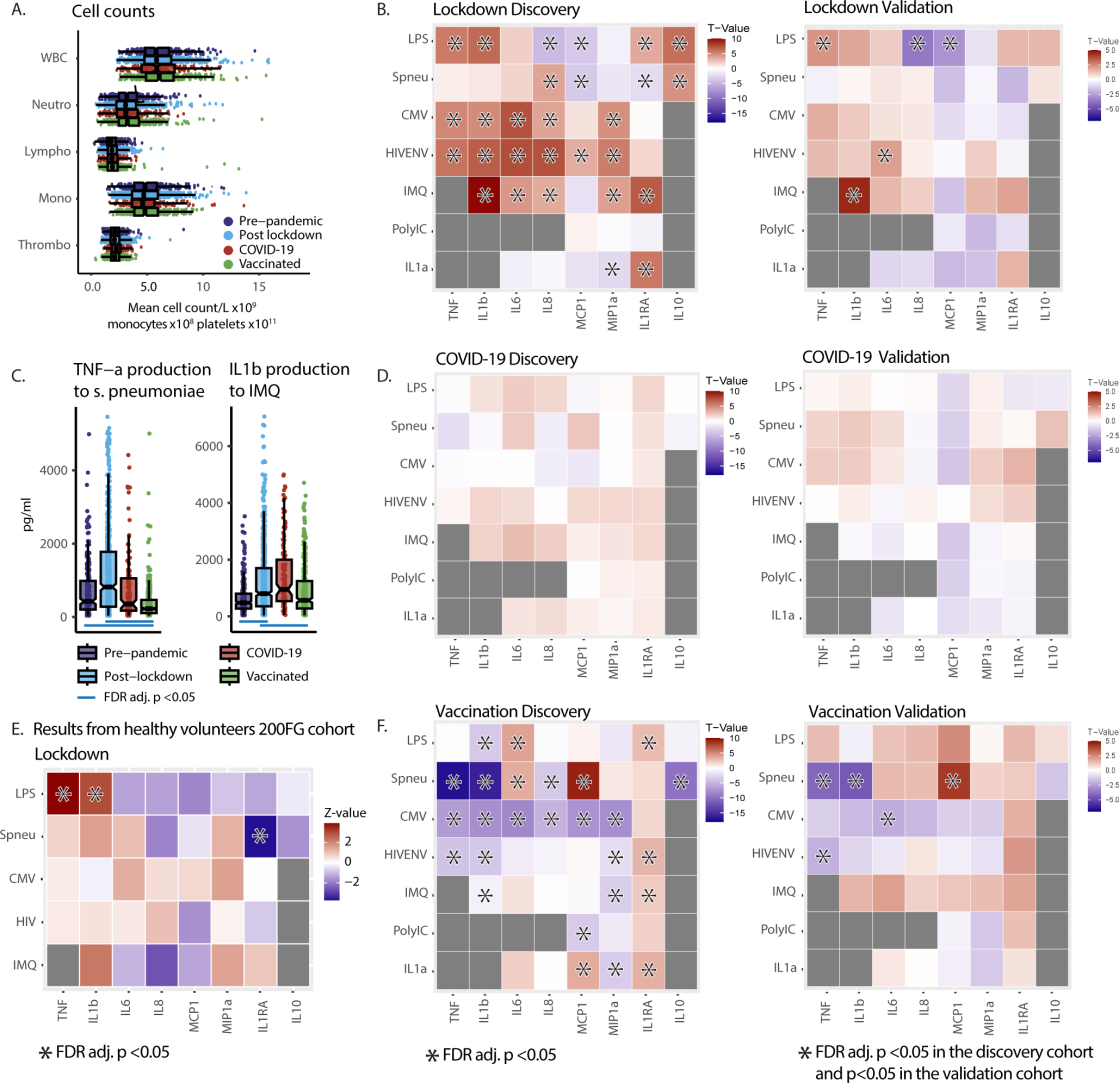
Pandemic’s impact on PBMC functional capacity in 2000HIV study participants and healthy subject (200FG) cohort **(A)** Cell counts from hemocytometry in PLHIV. Analysis of variance (ANOVA) per cell type showed no significant differences in cell counts between the groups. NB The X-axis scale differs to fit all cell types in 1 graph. WBC: white blood count [x10^9^/L]. Neutro: Neutrophils [x10^9^/L]. Lympho: Lymphocytes [x10^9^/L]. Mono: Monocytes [x10^8^/L]. Thrombo: Platelets [x10^11^/L]. **(B, D, F)** Heat Maps of cytokine production after 24-hour ex-vivo stimulation of PBMCs in the discovery and validation cohort of the 2000HIV study showing effects of pandemic. X-axis: cytokines. Y-axis: stimuli. Colors represent T-values. Red indicates higher cytokine production after lockdown **(B)**, COVID-19 **(D)** or vaccination **(F)**, blue represents lower production. Asterisks in discovery cohort: FDR adj. p <0.05. Asterisks in validation cohort: p <0.05 in validation and FDR adj. p <0.05 in discovery with same directionality: Results from ANCOVA on rank transformed data, adjusted for seasonality in all groups and also age and sex in COVID-19 group. **(C)** Box Scatter Plots showing untransformed TNF-α and IL-1β cytokine concentration in supernatants per group after 24 hour ex-vivo stimulation with S. Pneumoniae and imiquimod in the discovery cohort of the 2000HIV study. Blue line: FDR adj. p value <0.05 result from rank transformed ANCOVA analysis. **(E)** Heat Maps of cytokine production after 24-hours ex-vivo stimulation of PBMCs in healthy volunteers from 200FG cohort demonstrating a similar pattern of inflammatory cytokine production after the first lockdown. Colors are Z-values from pairwise Wilcoxon tests. Red indicates higher cytokine production after lockdown; blue lower. Asterisks mark FDR adj. p <0.05. HIVENV, HIV envelope; IMQ, imiquimod; pneu, heat killed S. Pneumoniae; TNF, TNF-α; MCP1, monocyte chemoattractant protein-1; MIP1a, macrophage inflammatory protein 1α.

Next, we evaluated the responsiveness of peripheral blood mononuclear cells (PBMCs) of PLHIV upon stimulation. First, hemocytometric blood analysis showed no differences in absolute monocyte, neutrophil, or lymphocyte cell counts between the groups before and during the pandemic (**Figure 3A**). Second, we observed a general upregulation of monocyte-derived cytokine production in response to a wide range of microbial stimuli post-lockdown. A total of five cytokine/stimuli pairs were statistically validated in the validation cohort, with an overall pattern of cytokine responsiveness consistently higher after lockdown in both the discovery and validation cohorts (**Figure 3B**).

In contrast, past COVID-19 infection did not exert a significant effect on immune responsiveness, in either the discovery or the validation cohort (**Figure 3D**). On the other hand, we observed a clear pattern of lower production capacity of TNF-α and IL-1β after COVID-19 vaccination, while the release of IL-1Ra was increased in response to almost all stimuli. Although only five cytokine/stimulus pairs were statistically validated in both cohorts, the direction of the effects between the discovery and validation cohorts was highly consistent (**Figure 3F**). Examples of effect sizes are shown in **Figure 3C**, and data for all cytokines can be found in **Supplementary Figures 2A–H**. These results demonstrate that the decreased systemic inflammation after lockdown as shown by the blood proteome analysis is accompanied by an increased production capacity of monocyte-derived cytokines after microbial stimulation. In contrast, after vaccination against COVID-19, a lower TNF-α and IL-1β production capacity was observed, complemented by higher anti-inflammatory IL-1Ra production capacity. It is important to note the timelines in which different groups were investigated: the vaccinated participants were recruited at later time points after subsequently easing of the restrictions, which may have resulted in a waning of immunological effects of the lockdown and return to pre-lockdown immune responsiveness. Indeed, analysis with an additional correction for the days since most recent lockdown initiation as a covariate, or selection of the participants recruited between 150 and 200 days since lockdown initiation, show that in this group the immunological changes can at least in part be explained by the waning of lockdown effects (**Supplementary Figure 2I**). Another approach to eliminate the effects of lockdown with respect to effects of vaccination is to investigate cytokines that were either not affected or impacted in the opposite direction by the lockdown, and show significant changes after vaccination compared to the pre-pandemic group. This analysis showed that TNF-α production to *S. pneumoniae* was lower after vaccination while it was unaffected by the lockdown, confirming a vaccination effect independent of the lockdown (**Supplementary Figure 2I**, **Figure 3C**).

### Increased cytokine responsiveness induced by lockdown can be replicated in a cohort of healthy individuals

The volunteers from the 2000HIV cohort are individuals on long-term antiretroviral treatment, virally suppressed, with generally normal CD4 T-cell levels and without any sign or symptom from an acute condition, which argues against the hypothesis that the observed changes in immune responsiveness are related to the HIV status of our participants. However, immune responsiveness may be different in PLHIV, even when using long-term ART. Therefore, we sought to validate our findings also in a cohort of healthy volunteers. We compared paired samples of PBMCs from 36 healthy donors harvested in 2020 after the lockdown and in October 2022, after the mitigation of all social distancing measures. After quality control, 30 samples were included in the analysis. Results from these healthy controls showed a similar pattern of increased production capacity of pro-inflammatory cytokines in response to most stimuli post-lockdown, providing evidence that these effects are generalizable also in healthy individuals, beyond PLHIV (**Figure 3E**).

### No differences between the effects of mRNA or adenovirus-based COVID-19 vaccines in PLHIV

As either mRNA or adenovirus-based vaccines were used at the beginning of the COVID-19 pandemic, and as both these vaccine platforms are new, we sought to investigate whether they have similar immunological effects. We did not see differences between the effects of these two types of vaccines: there were no consistent DAPs in the circulatory proteome between the two groups of PLHIV, and there was no consistency in the scatter plot showing effect size in the discovery versus validation cohort (**Supplementary Figure 1B**). There were also no significant differences in the immune cell responsiveness between PLHIV vaccinated with the two types of vaccines (**Supplementary Figure 2J**). An example of effect sizes for IL-1β showed similar amounts of cytokine production (**Supplementary Figure 2K**). That means that in our cohort the observed increased systemic inflammation and reduced functional capacity after vaccination is independent from the type of vaccine being used.

### T-cell responsiveness is not impacted by lockdowns, COVID-19 or vaccination in PLHIV

To assess T cell responsiveness, PMBCs of PLHIV were stimulated for 7 days with a range of microbial stimuli. In contrast to the effects in the innate compartment, only mild differences in single stimuli/cytokine pairs were detected, but no consistent or clear pattern of T cell dysfunction was observed after exposure to microbial stimuli (**Supplementary Figure 2l**). For example, IFNγ production was similar in all groups, the only exception being exposure to phytohaemagglutinin (PHA, **Supplementary Figure 2M**). However, PHA is a plant-derived lectin that does not mimic *in-vivo* microbial stimulations through antigen-presenting cells. Therefore, considering the absence of differences in IFNγ production after stimulation with other microbial or derived stimuli, it is likely that this does not represent an *in vivo* significant difference in T-cell function.

### DNA methylation changes exerted by lockdown, COVID-19 infection and vaccinations in PLHIV

**Figure 4:**
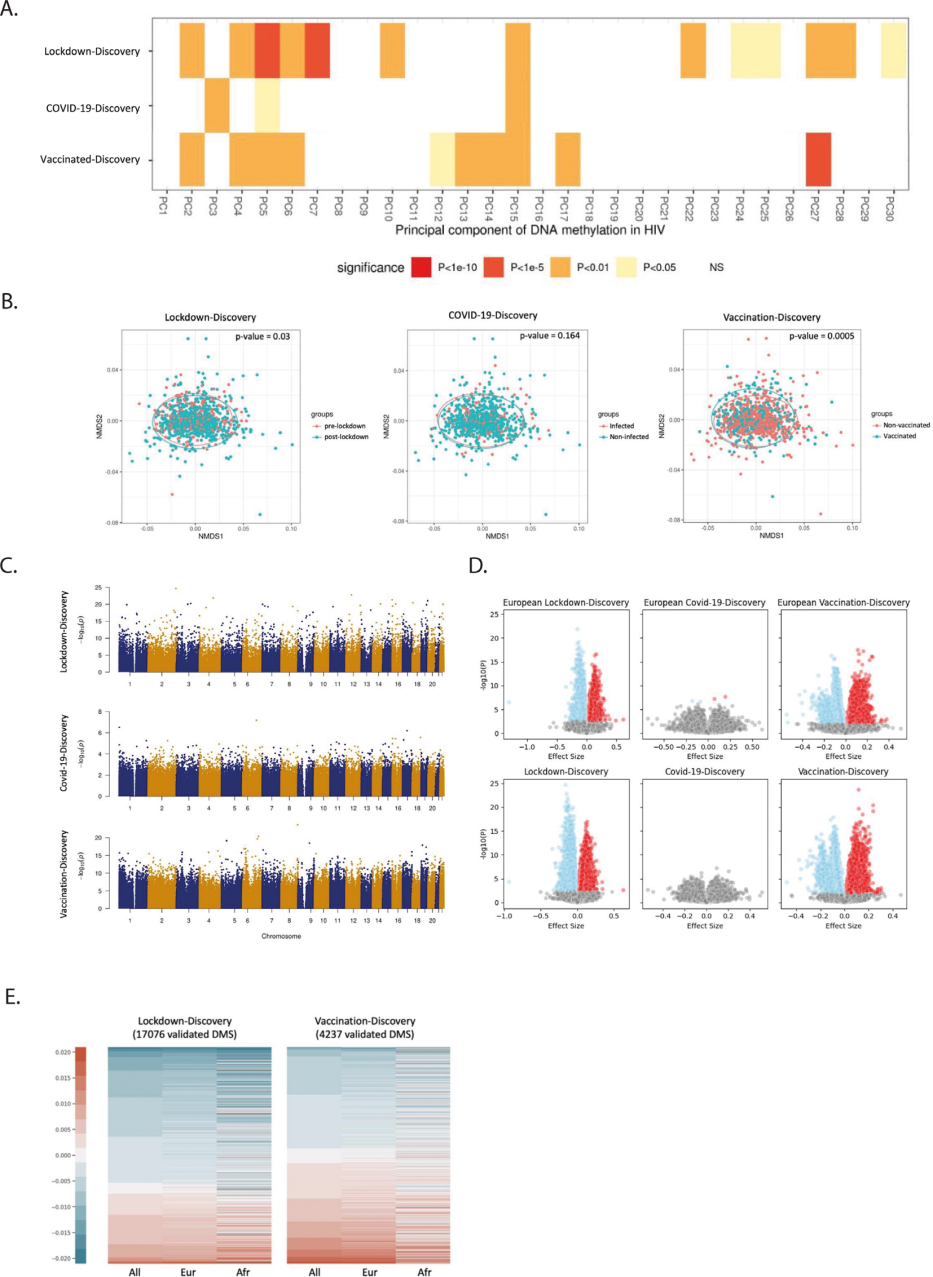
Influence of pandemic on DNA methylation in PLHIV. **(A)** Correlation between the top 30 PCAs and the variables (Lockdown, Covid-19, and Vaccination). FDR adjusted. Before- Lockdown: during-Lockdown = 279: 705; Covid-non-infected: Covid-infected = 705: 137; Non-vaccinated: vaccinated = 705: 330. **(B)** NMDS analysis of individuals in discovery cohort of the 2000HIV study with DNA methylation beta value variable greater than 0.001. Three dimensions and 1999 permutations were applied for this analysis. **(C)** Manhattan plot of the EWAS results in different studies on the 2000HIV cohort, –log10(p-value) of all the detected CpGs (x-axis) were plotted with the location (y-axis) through the genome. **(D)** Volcano plot of effect size and –log10(p-value) based on the EWAS results. Lower panels: entire study population. Upper panels: participants from European ancestry. Blue: negative effect size with FDR<0.05, red: positive effect size with FDR<0.05. **(E)** Lockdown and vaccination associated DMS changes among all population, the Europeans, and the Africans of the 2000HIV study. The DNA methylation beta value was used for this plot.

The baseline characteristic of the 2000HIV study populations included in DNA methylation analysis discovery and validation cohorts are presented in **Supplementary Tables S3**-**S6**. The same groups were analyzed as outlined in **Figure 1B**. This resulted in comparisons being conducted within the discovery cohort between 275 pre-lockdown and 705 post-lockdown volunteers, between 330 vaccinated and 705 non-vaccinated participants, and between 137 COVID-19 infected and 705 COVID-19 negative individuals. Following quality control of DNA methylation (see Methods for details), a total of 793,767 CpG sites were included in downstream analyses. **Figure 4A** displays the global variability of DNA methylation across various scenarios. In the volunteers of the 2000HIV cohort, the first 30 principal components (PCs) explained 27~29% of the variance in blood methylation. Notably, the heatmap in **Figure 4A** indicates a greater number of significant associations (as depicted by the color bar) between DNA methylation variability and lockdown/vaccination compared to COVID-19 infection. In addition, we used a nonmetric multi-dimensional scaling approach (NMDS) to illustrate the significant methylation difference between lockdown (adonis, R^2^ = 0.0028, p-value = 0.03, with 1999 permutations) and vaccination (adonis, R^2^ = 0.0029, p-value = 0.0005, with 1999 permutations) comparisons (**Figure 4B**). However, no significant difference between the COVID-19 infected vs non-infected individuals was observed (adonis, R^2^ = 0.0015, p-value = 0.164, with 1999 permutations). This result confirms the findings obtained from the PCA analysis, demonstrating that the overall greater effects on DNA methylation are exerted by lockdowns and vaccination, in contrast to SARS-CoV-2 infection which did not show significant effects after a median of 243 days post-mild infection.

Next, we conducted an Epigenome-Wide Association Study (EWAS) on each CpG site after correcting for age, sex, season effect, batch, immune cells counts and the first five PCs from genotype data of the same PLHIV when analyzing all populations (**Figure 4C**, **Supplementary Figures 3**, **4**). To address the potential inflation of the model, the BACON method was applied to control for bias and inflation. We applied the BACON adjustment (12) in the context of the lockdown and vaccination study. This adjustment reduced the inflation, but did not alter the ranking of the significance of the associations observed at the CpG sites (**Supplementary Figures 5**, **6**). In the analysis of the lockdown effects, the discovery EWAS analysis identified 57,730 genome-wide significant CpG sites influenced by lockdowns. 17,076 out of 57,730 CpG sites have been replicated in the validation cohort. Among these sites, cg08255374 annotated to *UBE2F* and *RAMP1* genes showed the most significant association (p-value =2.13 × 10^-25^, **Supplementary Tables S7**, **S8**).

Subsequently, we investigated the DNA methylation changes induced by COVID-19 infection and vaccinations in the 2000HIV study. We did not detect any genome-wide significant CpG sites associated with COVID-19 in the discovery cohort (**Figure 4C**). In contrast, the vaccination analysis identified 162,993 significant CpG sites that were impacted by the COVID-19 vaccines, with cg10675725, annotated to *GSDMD* and *MROH6*, showing the strongest association (p-value = 7.42 × 10^-20^, **Figure 4C**, **Supplementary Table S9**). Among them, 4,237 out of 162,993 CpG sites have been replicated and the most significant validated CpG site is cg12578536 which is annotated to gene *SEPP1* and *ANXA2R* (p-value= 7.42 ×10^-20^, **Supplementary Table S10**). When considering only mRNA vaccines in the analysis, similar effects on DNA methylation were observed compared to when all vaccines were included. (**Supplementary Figure 7**). However, when we compared mRNA vaccines to viral vector vaccines (**Supplementary Figure 8**, **Supplementary Table S11**), we identified two CpG sites (cg13510475 with p-value = 1.44 ×10^-8^ and cg04058821 with p-value =6.77 ×10^-8^) that were significantly different at the genome-wide level, but they could not be replicated in the validation cohort.

These findings suggest that lockdown and vaccination, but not COVID-19, induce important DNA methylation changes for more than 3 months after the event. As DNA methylation patterns have been observed to vary among different ethnic groups (13), we conducted an EWAS study which only included volunteers of European ancestry. Based on the volcano plots presented in **Figure 4D**, it is evident that ancestry does not exert a major influence on our overall differential methylation pattern. The volcano plots for the effects of lockdowns and vaccination exhibit a distinct separation between upregulated and downregulated CpG sites. In contrast, the volcano plot for COVID-19 infection reveals a more dispersed pattern, with only four significant CpG sites (cg21464724, cg19416239, cg24678928, and cg15772223) being identified in the European population and none across all population (**Figure 4D**, **Supplementary Table S12**). Comparing the impact of lockdown and vaccination on DNA methylation, we observed an overall effect of increasing DNA methylation by vaccination (p-value =1.12×10^-10^, one-sided proportional test) and an overall effect of decreasing DNA methylation by lockdowns (p-value < 2.2×10^-16^, one-sided proportional test), as depicted by the ratio of the blue and red color bar in **Figure 4E**.

**Figure 5:**
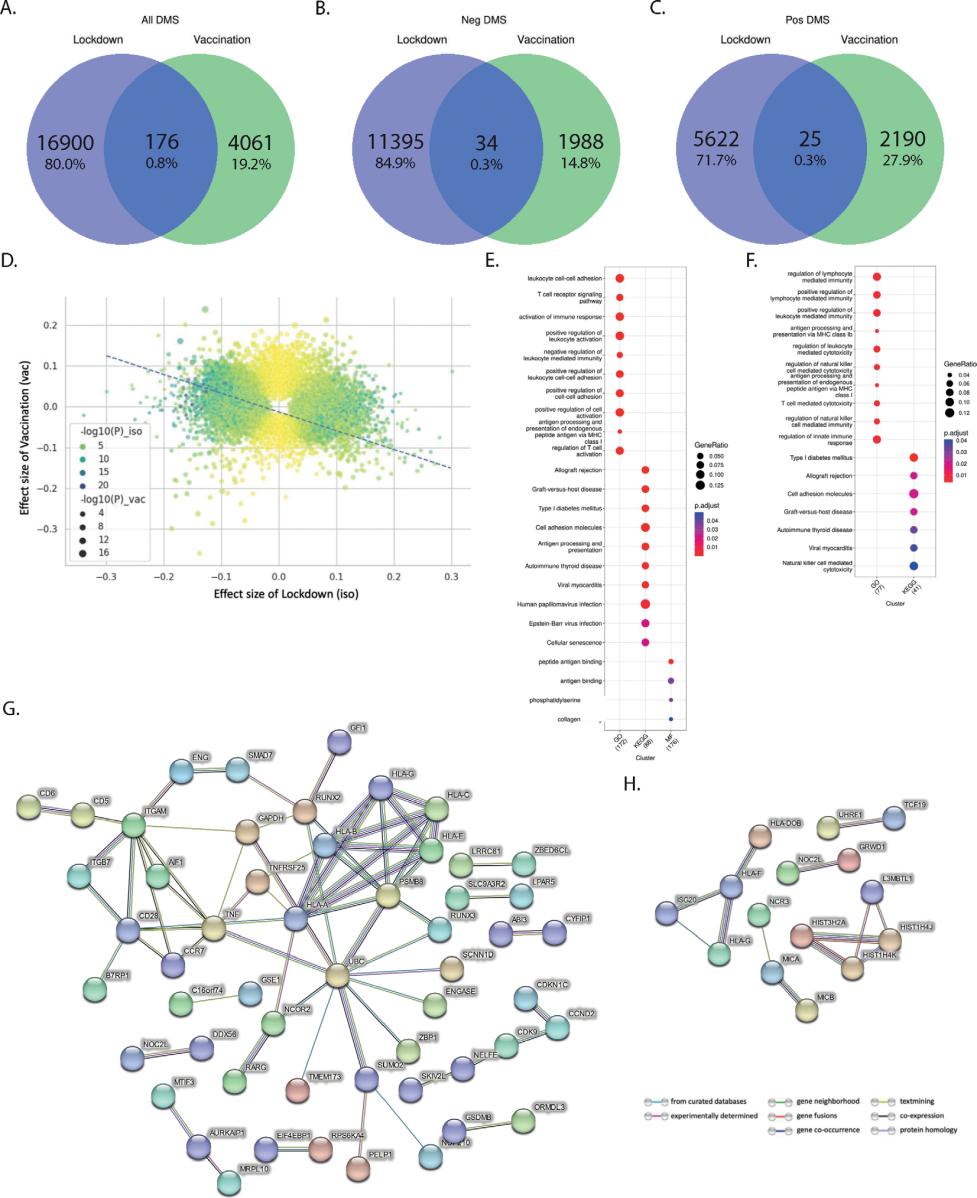
Distinct effects of vaccination and lockdown on DNA methylation in PLHIV. **(A–C)** Comparison between the number of validated DMS in lockdown and vaccination in all ethnicity of the 2000HIV study: **(A)** total number of DMS; **(B)** DMS having negative effect size; **(C)** DMS having positive effect size. **(D)** Correlation between the effect size of the lockdown and Vaccination: Residual standard error: 0.06263 on 21135 degrees of freedom, Multiple R-squared: 0.091, Adjusted R-squared: 0.091, p-value: < 2.2e-16, 14779 DMS are in different direction, 6358 DMS are in the same direction. **(E, F)** Pathway enrichment analysis associated with DMS that were present in ciseQTMS and also influenced due to **(E)** lockdown (209 DMS associated with 223 Genes) and **(F)** Vaccination (72 DMS associated with 102 Genes) in all ethnicities of the 2000HIV cohort. **(G, H)** Interaction (confidence >0.7) between gene products, i.e. proteins, that are associated with **(G)** lockdown and **(H)** Vaccination based on the cis-eQTMS database.

Next, we aimed to assess in the 2000HIV study whether the effects of lockdowns and vaccination were similarly exerted on the same loci, but in different directions. We first compared the CpG sites between the two scenarios and found very little overlap between the loci affected (**Figures 5A–C**, 0.8% in all directions, 0.3% in the positive direction, and 0.3% in the negative direction), which suggests different epigenetic changes between lockdown and vaccination. When comparing the effect size of significant CpG sites in the two scenarios, we found a clear inverse correlation (p-value < 2.2×10^-16^, adjusted R^2^ = 0.091, **Figure 5D**), which suggests that vaccination may have a partly inverse epigenetic effect compared to the effects of the lockdown.

We then used the BIOS QTL browser (14) to link gene expression with the identified CpG sites and performed a pathway enrichment analysis. Our findings showed that lockdowns mainly impacted genes enriched in regulation of immune effector process, T cell and leukocyte function. On the other hand, vaccination-influenced loci were enriched in pathways associated with lymphocyte mediated immunity, leukocyte and T cell mediated cytotoxicity (**Figures 5E, F**, and **Supplementary Figures 9**, **10**). Further, interaction analysis between the gene products, i.e., proteins, associated with the gene expression via STRING database (v11.5) (15) suggested that the effects of lockdowns may be mediated by regulation of ubiquitinilation processes (**Figure 5G**) (16).

Protein-protein interaction analysis of the effects exerted by anti-COVID-19 vaccination revealed one cluster (**Figure 5H**) comprised mainly of various HLA types, i.e., HLA-DOB, HLA-F, HLA-G, and ISG20. Earlier studies have also demonstrated the association of HLA type with COVID-19 vaccine antibody response (17). Experts have also suggested taking HLA genotype into account when designing SARS-CoV-2 vaccines to enhance effectiveness through T cell immunity, especially across diverse ethnic populations, and to potentially use them as a booster to reinforce immune responses (17).

Finally, as also mentioned earlier, because of the timeline of the study, the individuals in the COVID-19 vaccination group of the 2000HIV study were recruited at a later time point after lifting the lockdown restrictions, compared to the non-vaccinated group (**Figure 1A**). Therefore, one cannot exclude that some of the effects observed in the vaccination group may be attributable to the different kinetics of the waning of lock-down effects in the two groups. To investigate this, we conducted an EWAS involving COVID-19 vaccinated individuals from a narrower timeframe after the lock-down (**Supplementary Figure 11A**). Despite the continued significant difference in days-post-lockdown between the vaccinated and non-vaccinated groups within this narrowed timeframe, no genome-wide significant (FDR<0.05) differences in methylation at CpG sites were detected (**Supplementary Figures 11B, C**). Therefore, although the loss of power due to the smaller number of individuals in this verification analysis may explain the less strong effects on DNA methylation, we conclude that the dramatic epigenetic changes observed between vaccinated and non-vaccinated PLHIV were at least partly attributable to the waning of the lockdown effects.

## Discussion

In this study we report broad effects of various public health interventions during the COVID-19 pandemic on immune responses at a population level in a large cohort of people living with HIV in the Netherlands. On the one hand, the systemic inflammatory status, as assessed by targeted proteomics, decreased during the lockdown periods, while rebounding after the COVID-19 vaccination campaigns. On the other hand, the immune responsiveness as assessed by cytokine production capacity of circulating innate immune cells was strongly upregulated during lockdowns and was downmodulated by COVID-19 vaccines. These effects are likely mediated through changes in epigenetic regulation: lockdowns were associated with a very significant loss of DNA methylation in immune cells, while this effect was partially (although not completely) reversed during the vaccination campaigns. It is important to underline that the main cohorts in the present study consisted of PLHIV, and thus the conclusions can be mainly applied to this population. However, the most important immunological results are also validated in an independent cohort of healthy individuals, which suggest that similar effects are likely true in the general population, although more studies are needed to fully establish that.

The impact of the COVID-19 pandemic on public health has been profound and was exerted at multiple levels: direct impact of the infection itself on morbidity and mortality, decrease in the availability of medical care for non-COVID-19 diseases, and the psychological impact of social isolation measures (18, 19), just to name a few of these effects. One aspect that received less attention is the long-term impacts of both infection and public health measures (lockdowns, masks, vaccinations) on human immune responses in general and their possible influence on overall morbidity and pathology. Social interaction is one of the most important features of human societies and it is crucial for psychological and societal well-being. In addition, these contacts also determine the level of exposure to microorganisms and the resulting infections, which in turn can have immune modulating effects that impact various immune-mediated diseases (20). Continuous exposure to environmental microorganisms is important for the fine-tuning of immune responses, and immune dysregulation may follow after a lack of exposure to environmental stimuli, including micro-organisms. Extreme situations such as a complete lack of exposure in germ-free mice lead to inappropriate development of the immune system, and exacerbated responses upon inflammatory challenges (21, 22). In humans, lack of exposure to environmental cues in modern societies is thought to contribute to the increased incidence of autoimmune and allergic diseases (the Hygiene Hypothesis) (23–25).

From this perspective, the impact of lockdowns on general immune responses is very relevant. The decrease of systemic inflammatory biomarkers in the circulation during the lockdowns is expected, as a mirror of the decreased exposure to day-to-day microbial exposure. In itself, this decrease of systemic inflammation is unlikely to be deleterious as long as the host is not challenged with an immune stimulant. The concern arises when observing the strongly upregulated (or dysregulated) cytokine responses upon exposure to both microbial and non-microbial stimuli of immune cells isolated from volunteers after lockdown (**Figures 3B, E**). These overreacting immune responses, characterized by strongly increased production of proinflammatory cytokines and chemokines, were consistently observed in the discovery and validation cohort of the 2000HIV study and confirmed in a limited number of healthy individuals. The consequences of this immune hyperresponsiveness for infectious, inflammatory and allergic diseases upon the return of the population to the normal social interactions are not known, but they should be seriously considered and further investigated.

In addition to the effects of social isolation and diminished infectious pressure, COVID-19 itself and the vaccination campaigns with the new vaccines are also likely to modulate immune responses. The effect of past COVID-19 itself on immune responses was limited. The lack of a strong effect is likely due to the relatively long period between the infection and the measurements of inflammatory parameters, as well as the fact that the COVID-19 infections were mild or asymptomatic (with the exception of three participants). In contrast, stronger long-term effects were observed by COVID-19 vaccinations. The vaccines increased the concentrations of inflammatory proteins in the circulation, and in parallel induced a significant down-modulation of the inflammatory response (especially TNF-α and IL-1β production), while the release of the anti-inflammatory IL-1Ra release was increased. Interestingly, these effects were induced similarly by both mRNA- and adenoviral-based COVID-19 vaccines. Such changes are in line with several studies suggesting a broad effect of both COVID-19 mRNA and adenoviral vaccines on innate immune responses at transcriptional (26, 27) and functional (28) levels. The lipid nanoparticle (LNP) component of mRNA vaccines was reported to induce strong systemic pro-inflammatory responses (29), and recent studies have shown that BNT162b2 can also induce long-term transcriptional changes in myeloid cells (30). This suggests that the response of immune cells against various microorganisms other than SARS-CoV-2 could also change after BNT162b2 vaccination, as reported recently (31).

An important aspect relates to the likely mechanisms responsible for the effects of lockdowns and vaccines. On the one hand, direct causality cannot be conclusively proven due to the study’s cross-sectional design, this is difficult to further investigate due to obvious ethical considerations related to *in-vivo* pathway modulation or randomized/experimental designs in human studies involving exposure to lockdowns or infection. On the other hand, however, important mechanistic clues are given by the strong changes in DNA methylation that argue for epigenetic processes as the molecular substrate of these effects. DNA methylation is usually associated with transcriptional repression (32), and extensive literature documents the epigenetic changes associated with a wide range of environmental exposures, including exposure to smoking (33), air pollution (34) and infection (35). In this study, we have demonstrated, for the first time, the impact of lockdowns and COVID-19 vaccination on DNA methylation patterns. Importantly, the strongest effects during lockdown showed loss of DNA methylation (likely contributing to the increased responsiveness upon stimulation), while vaccination led mostly to an increased DNA methylation pattern (that could contribute to gene repression).

It is also interesting to identify the pathways which were most strongly modulated at the DNA methylation level. Lockdowns induced DNA methylation changes in genes important for anti-viral responses, which could be expected. In addition, vaccination induced DNA methylation changes were especially found in pathways related to lymphocyte and T cell function. One important factor when assessing the differences in DNA methylation between vaccinated and non-vaccinated individuals was the longer time interval after the lockdown in the vaccinated group, which may argue that some of the vaccination effects were in fact the result of waning of the lockdowns effect. Importantly, however, the changes induced by lockdown and vaccination, respectively, were not simply antagonistic: the vaccination did not merely reverse some of the changes induced by the lockdown but induced an own distinctive pattern. Three years into the pandemic, the DNA methylation status of the individuals is in a different state than before the pandemic. This conclusion is also supported by the blood proteomics data: the inflammatory status was not reversed by the vaccination, but it is translated into a different profile than before the pandemic.

Our data are derived from a cohort of PLHIV who are virally suppressed and on long-term ART. Although it is known that immune responsiveness of PLHIV is different from healthy population, even when virally suppressed and without any symptoms (36), the immunological effect of COVID-19 pandemic and its measures to mitigate it, are likely similar in PLHIV and healthy population. The successful replication of the functional immunological changes in a small group of 30 healthy volunteers suggests that our findings may also be applicable to the general population, although the number of included subjects was small and we had no samples to validate the proposed underlying mechanisms. Therefore, more data are needed to further demonstrate generalizability of the immune changes observed in the general population. An additional limitation of this research is its cross-sectional design, as observational studies inherently carry the risk of confounding factors. We aimed to minimalize their influence by using thorough correction methods and independent validation cohorts.

In conclusion, the present study demonstrates that immune responses in two independent cohorts of PLHIV underwent significant changes during the pandemic. The role of epigenetic factors in these changes was shown as well. Furthermore, the changes in immune responses were confirmed in a small cohort of healthy subjects, suggesting that our results may be applicable to the general population. Our data in PLHIV strongly suggest that the imposition of social interaction limitations such as lockdowns led to an altered regulation of inflammatory responses, most likely due to a lack of normal exposure to environmental stimuli. This may lead to exacerbated immune responses in allergies or immune-mediated diseases, not unlike the changes predicted by the hygiene hypothesis. Other changes such as modified patterns in physical activity or diet that have been reported during the lockdown (37, 38) could also play a role in the altered immune responses. A reverse of this situation has been recently reported in Indian populations, in which higher infectious pressure ensures a more tolerant immune response, which may well be responsible also for the lower morbidity during the pandemic (39). All these data suggest therefore that infectious pressure continuously modulates the immune system and that limitations of social interactions to prevent exposure to infectious agents, such as the lockdowns, has broad consequences on the immune system and may as such have unforeseen medical consequences, apart from the obvious psychological concerns. In addition, the COVID-19 vaccines that use the new mRNA and adenoviral technology platforms have immunological effects that are broader than anti-SARS-CoV-2 effects exerted through specific antibodies or T-cells, and their impact on non-COVID-19 immune-mediated pathology should be studied in the years to come.

## Methods

### Cohorts

This study uses data from two ongoing studies within the Human Functional Genomics Project: 2000HIV and 200FG. Study protocols have been approved by the Medical Ethical Review Committee Oost Nederland, Nijmegen, the Netherlands under the registration NL68056.091.18 (2000HIV) and 2018-399 EC (200FG). All participants provided their written informed consent prior to participation in the study. Experimental protocols were conducted following the principles of the Declaration of Helsinki.

The 2000HIV study cohorts and experimental methods have been extensively described by Vos et al. (9). It is an observational study, in which between 2019 and 2022, 1895 virally suppressed asymptomatic PLHIV were enrolled in two separate cohorts (a discovery and validation cohort, based on recruitment center). The discovery cohort was recruited in three specialized Dutch HIV treatment centers, two university medical centers and one large general hospital (*Radboudumc Nijmegen, Erasmus MC Rotterdam, and OLVG Amsterdam*). Participants in the validation cohort were recruited in a separate HIV expertise center, a large general hospital (*Elisabeth-TweeSteden Ziekenhuis Tilburg*). Although the samples of the two subcohorts were collected separately, processing and measurements were identical. Extensive multi-omics characterization was performed on immune cells isolated from the participants, as well as registration and measurement of clinical parameters and medical history. Cross-sectional data from the baseline visits were used. The 200FG cohort comprises of healthy individuals >18-year-old enrolled in 2018 from whom yearly samples are collected.

### Measurement methods

#### Blood collection

For the 2000HIV study participants, blood was collected via venipuncture during the baseline study visit in four different study centers in the Netherlands. Samples were transported to the laboratory at Radboudumc, Nijmegen, overnight at room temperature.

#### Hemocytometry

Hemocytometry was performed on whole blood with the XN-1000 Sysmex haematology analyzer.

#### PBMC ex-vivo stimulation

For stimulation experiments, peripheral blood mononuclear cells (PBMCs) were isolated using Ficoll-Paque density centrifugation. PBMCs were subsequently incubated in U-bottom 96-well plates at 0.5 × 10^6^ cells/well with various bacterial, fungal, and viral stimuli (**Supplementary Tables S15**, **S16**) at 37°C and 5% CO2, for either 24 hours or 7 days, after which supernatants were stored -20 °C. ELISAs were done on supernatants after conclusion of recruitment to determine IL-1β, IL-1Ra, IL-6, IL-8, IL-10, MCP-1, MIP-1a and TNF-α concentration in the 24-hour experiment, and IL-5, IL-10, IL-17, IL-22 and IFN-γ concentrations in the 7-day experiment (Duoset ELISA, R&D Systems). Based on pilot experiments some cytokines were not measured after stimulation with certain stimuli. The same stimulation panel and protocol was used on cryopreserved PBMC samples from 36 out of 101 200FG participants that donated blood in both October 2020 and October 2022.

#### Targeted proteomics

Plasma samples from both cohorts were used to measure 3072 targeted plasma proteins with Olink^®^ Explore panel (40). Olink uses proximity extension assay technology to measure relative concentrations of proteins, presented as log2 normalized protein abuncance level (NPX).

#### DNA methylation

DNA methylation was performed on a total of 1914 samples. DNA was isolated from EDTA whole blood by the Radboudumc Genetics Department using ChemagicStar automated configuration (consisting of the Microlab STAR and Chemagen Magnetic Separation Module 1, Hamilton Robotics) combined with Chemagen nucleic acid extraction technology with magnetic polyvinyl alcohol (M-PVA) beads, which follows a standard and automated bind-wash-elute procedure. The concentration of the DNA and 260/280nm ratio were determined using NanoDrop spectrophotometer, after which samples were normalized to 50 ng/µL in TE-buffer and randomly distributed amongst plates. High-quality DNA were selected for genome-wide DNA methylation profiling using the Illumina Infinium MethylationEPIC BeadChip array (MethylEPIC v1 manifest B5). Standard sample- and probe-based quality control were performed.

### Analysis

We compared unvaccinated COVID-19-negative PLHIV before and after the first lockdown in the Netherlands, unvaccinated PLWH with and without past COVID-19 infection, and PLWH with or without anti-COVID-19 vaccination, excluding those with past COVID-19 infection. Participants who had both a positive coronavirus status and were vaccinated (n=63), had no COVID serology measured (n=7), had positive COVID serology before the pandemic (n=7) or were on immunosuppressants (n=20) were excluded from our analysis, resulting in 1478 and 320 participants in the discovery and validation cohort respectively for downstream analysis (**Figure 1B**).

As a validation, cytokine production capacity in the healthy volunteers from the 200FG cohort was compared between years 2020 (at the height of pandemic lockdowns) and 2022 (after the lifting of lockdown restrictions).

### Proteomic analysis of the plasma

#### Processing

Protein concentrations in plasma samples from PLHIV were measured by proximity extension assay (Olink) in three batches. Bridging normalization was used to remove batch effects, whereafter standard quality control per protein and sample was performed. In **Supplementary Figure 12A** this process shown in detail. In each of the eight panels from the Olink^®^ Explore 3072 platform, IL-6, TNF-α, CXCL8, LMOD1, SCRIB, IDO1 were measured as technical duplicates for quality control purposes. Strong correlations were observed between the technical duplicates among panels, and therefore, we selected the measurements from the inflammatory panel. Next, we excluded proteins with limit of detection (LOD) ≥ 25 of the samples (n = 547 proteins were excluded), resulting in 2367 proteins for downstream data analysis.

During quality control (QC) per sample, we performed principal component analysis (PCA) using the NPX. Outliers were defined as those samples falling above or below four standard deviations (SD) from the mean of principal component one (PC1) and/or two (PC2). In total, seven samples were excluded based on PCA, resulting in 1777 samples analyzed. The overview of QC process is depicted in **Supplementary Figure 12B**.

#### PCA analysis

PCA analysis per group was performed on residuals after adjusting for sex and age using all protein NPX values as input. Wilcoxon sum rank test was used to compare distributions.

#### Differential abundance (DA) analysis

NPX values were compared between the groups of interest using a linear model with age, sex and seasonality as covariables. It has been previously described that annual seasonality is an important environmental factor influencing circulating cytokine concentrations and therefore, we corrected for seasonality effect using a harmonic model as described previously (41). P-values were adjusted for multiple testing comparisons using a false discovery rate (FDR) method and proteins with FDR adjusted p-value <0.05 and p-value <0.05 were considered statistically significant in the discovery and validation cohort, respectively. Additionally, scatter plots showing log-fold change in the validation and discovery cohorts were used to show consistency of directionality and effect size, which are not affected by the unequal group sizes. For DE analysis, we used the R package Limma adjusted to protein data, which is originally being used for the analysis of gene expression data (42). Limma uses an empirical Bayes method to moderate the standard errors of the estimated log- fold changes. A full list of validated DEPS is added in **Supplementary Tables S13** and **S14**.

#### Pathway enrichment analysis

Functional pathway enrichment analysis of validated (i.e. same direction and FDR adj. P <0.05 in discovery and P < 0.05 in validation cohort) differentially abundant proteins (DAPs) was performed using the DAVID bioinformatics tool, with the KEGG and Reactome library used as a reference library. A reference gene list of the genes that encode for the proteins measured with the OLINK Explore panel was used. Pathways were considered significant with a p-value <0.05 and protein count >3. Results are shown as a bubble plot using ggplot2 package, and as a network made using “enrichment map’’ in Cytoscape. Network nodes represent pathways and weighted edges represent the degree of gene overlap score between two pathways.

#### Gene set enrichment analysis (GSEA)

GSEA was performed using R package fgsea. We used the following strategy: regardless of P values, proteins that were expressed in similar direction in both the validation cohort and the discovery cohort were assigned a rank based on the t-statistic. GSEA was performed with KEGG, Reactome, and Hallmark reference libraries, using all measured proteins as a background.

### PBMC cytokine production *ex vivo* stimulation

#### Processing

24-hour experiment: Samples from 1742 participants were measured, of which 42 samples were excluded for being RPMI positive (which was the negative control), defined as having concentrations of above 2x lower limit of detection (LLOD) after RPMI stimulation in two out of TNF-α, IL-1β or IL-6. Outliers on PCA, defined as those +/- 4SD from the mean in PC1 and/or PC2, were removed (n=13). Data from the resulting 1687 participants was used in downstream analysis. 7-day experiment: Samples from 1744 participants were measured, of which 42 samples were excluded for being RPMI positive and 20 outliers on PCA analysis were removed (as in the 24-hours experiments). Data from a total of 1682 participants was used in downstream analysis. Healthy cohort (200FG): Samples from six out of 36 volunteers were excluded as they were RPMI positive in either the measurements from the 2020 or 2022 sample. Prioritization of stimuli with respect to PBMC yield was predetermined, and stimulation with RPMI, LPS, and IMQ was performed on all 30 participants, *S. Pneumoniae* on 26, CMV (pp65) on 15, and HIV-ENV on 9 participants’ samples.

#### Analysis

24 hour and 7-day experiment 2000HIV: Groups were compared using analysis of covariance (ANCOVA) on rank transformed data, implemented in the base R package ‘stats’. QQ plots before and after transformation are in **Supplementary Figure 2N**. The following covariables were considered as potential confounders: age, sex, seasonality [harmonic model (11)], ethnicity, latest CD4 count, BMI, current smoking status, center of inclusion, and latest viral load. Backward stepwise regression was performed to identify relevant covariates, which were included in the model if they met the following criteria: significance (p > 0.05) and a change >10% in the β-coefficient of the grouping variable. This criterion was met by seasonality in all groups, and age and sex in the COVID groups. The rank based ANCOVA model was adjusted for these covariates within their respective groups. The resulting t-values were visualized as colors, and p-values <0.05 were indicated with stars. Multiple testing adjustments were performed using the Benjamin Hochberg false discovery rate (FDR) method in the discovery cohort. For 24-hour experiment data of the healthy cohort, statistical testing was conducted using Wilcoxon’s signed-rank test on the paired samples. The analysis compared observations from October 2020 (post-lockdown) and October 2022 (normal situation well after mitigation of social distancing measures).

### DNA methylation

#### Processing

As previously described (9), the DNA methylation dataset was divided into a discovery cohort (n=1,546) and a validation cohort (n=322), and each cohort was analyzed separately. DNA methylation values were estimated from the raw IDAT files using the minfi package in R (v.4.2.0) (43). Preprocessing steps was done to discard two gender mismatch samples from discovery cohort, one bad quality samples from validation cohort (call rate < 99%). Probes (Discovery: n=2,743 and Validation: n=2,641) with methylation value missing (detection P>0.01) at >10% samples and probes within the sex chromosomes (n=19,627) were also excluded from the downstream analysis (44). Since the majority of the participants are European, we also removed the probes containing SNPs at the target CpG sites with a MAF>5% in European populations as well as the probes that mapped to multiple loci, i.e. polymorphic probes as suggested in (45) (Both Discovery and Validation: n=52,173).

#### Analysis

Next, we implemented stratified quantile normalization (46). Methylation value was also utilized for estimating proportion of six immune cell types, namely neutrophils, monocytes, B-Cells, NK cells, CD8-T cell and CD4 T cells, using modified Housman’s method available within the estimateCellCounts2 function of the FlowSorted.Blood.EPIC package of R (47). Methylation β‐values were calculated as a percentage: β = M/(M + U + 100), where M and U represent methylated and unmethylated signal intensities, respectively, and β‐values were then transformed to M‐values as log2(β/(1 − β)), and M‐values were used in all downstream analyses.

To mitigate the effect of extreme outliers in data, we trimmed the methylation set using: (25th percentile − 3*IQR) and (75th percentile + 3*IQR), where IQR = interquartile range. Differentially methylated CpG sites associated with lockdown, COVID-19 infection, and vaccination were identified by fitting a robust linear regression model. For lockdown and vaccination-associated EWAS results, we also considered the presence of surrogate variables by utilizing the SVA package (version 3.40.0) with leek method. However, no surrogate variables were found to have a significant impact (the estimated surrogate variable number was 0). The methylation M value was used as the outcome variable, and the model was corrected for age, sex, season effect, technical covariables, and immune cell proportions and surrogate variables (48). Surrogate variables were only considered for the analysis lockdown and vaccination effects due to high inflation of the model, and the SVA package (version 3.40.0) with leek method was employed. However, no surrogate variables were found to have a significant impact (the estimated surrogate variable number was 0). For EWAS with all ethnicities, the top five PCs extracted from the genotype of same individuals were included in the model for the correction of ethnicity.

CpGs were considered significantly replicated only if they have (i) Discovery cohort: FDR < 0.05, (ii) Validation cohort: same direction as in the discovery cohort and p-value < 0.05 and (iii) meta-analysis: same direction as in the discovery cohort, p-value < 0.05 in validation cohort and FDR (meta-analysis) <0.05. Standard error-weighted meta-analysis was performed with METAL (49). BIOS QTL browser (14) were further used to explore the correlation between CpG methylation and genes expression. Replicated CpGs associated genes were subjected to enrichment analysis using the clusterProfiler package (50) in R. Interaction between these genes’ product, i.e., protein, was carried out using the STRING database (51).

False Discovery Rate in this study was performed using the Benjamini-Hochberg procedure. The desired FDR threshold was set up to 0.05. The proportion test was performed with the pro.test function in the stats package (version 3.6.2) in R. All the plots in this study were plotted either with ggplot2 (version 3.4.2) package in R or seaborn (version 0.12.2) package in Python.

## Data Availability

The original contributions presented in the study are publicly available. This data can be found here: https://data.ru.nl/collections/ru/rumc/2000hiv_r0004571_dsc_373.

## References

[1] Wu F Zhao S Yu B Chen YM Wang W Song ZG. A new coronavirus associated with human respiratory disease in China. Nature. (2020) 579:265–9. doi: 10.1038/s41586-020-2008-3 PMC7094943 32015508

[2] Zhou P Yang XL Wang XG Hu B Zhang L Zhang W. A pneumonia outbreak associated with a new coronavirus of probable bat origin. Nature. (2020) 579:270–3. doi: 10.1038/s41586-020-2012-7 PMC7095418 32015507

[3] Loyal L Braun J Henze L Kruse B Dingeldey M Reimer U. Cross-reactive CD4(+) T cells enhance SARS-CoV-2 immune responses upon infection and vaccination. Sci 374 eabh1823. (2021). doi: 10.1126/science.abh1823 PMC10026850 34465633

[4] van de Veerdonk FL Giamarellos-Bourboulis E Pickkers P Derde L Leavis H van Crevel R. A guide to immunotherapy for COVID-19. Nat Med. (2022) 28:39–50. doi: 10.1038/s41591-021-01643-9 35064248

[5] Saravolatz LD Depcinski S Sharma M. Molnupiravir and nirmatrelvir-ritonavir: oral coronavirus disease 2019 antiviral drugs. Clin Infect Dis. (2023) 76:165–71. doi: 10.1093/cid/ciac180 PMC9383515 35245942

[6] Peluso MJ Deitchman AN Torres L Iyer NS Munter SE Nixon CC. Long-term SARS-CoV-2-specific immune and inflammatory responses in individuals recovering from COVID-19 with and without post-acute symptoms. Cell Rep. (2021) 36:109518. doi: 10.1016/j.celrep.2021.109518 34358460 PMC8342976

[7] Furman D Campisi J Verdin E Carrera-Bastos P Targ S Franceschi C. Chronic inflammation in the etiology of disease across the life span. Nat Med. (2019) 25:1822–32. doi: 10.1038/s41591-019-0675-0 PMC7147972 31806905

[8] Netea MG Joosten LA Li Y Kumar V Oosting M Smeekens S. Understanding human immune function using the resources from the Human Functional Genomics Project. Nat Med. (2016) 22:831–3. doi: 10.1038/nm.4140 27490433

[9] Vos W Groenendijk AL Blaauw MJT van Eekeren LE Navas A Cleophas MCP. The 2000HIV study: Design, multi-omics methods and participant characteristics. Front Immunol. (2022) 13:982746. doi: 10.3389/fimmu.2022.982746 36605197 PMC9809279

[10] Li Y Oosting M Deelen P Ricaño-Ponce I Smeekens S Jaeger M. Inter-individual variability and genetic influences on cytokine responses to bacteria and fungi. Nat Med. (2016) 22:952–60. doi: 10.1038/nm.4139 PMC5084084 27376574

[11] Ter Horst R Jaeger M van de Wijer L van der Heijden WA Janssen AMW Smeekens SP. Seasonal and nonseasonal longitudinal variation of immune function. J Immunol. (2021) 207:696–708. doi: 10.4049/jimmunol.2000133 34261668

[12] van Iterson M van Zwet EW Heijmans BT. Controlling bias and inflation in epigenome- and transcriptome-wide association studies using the empirical null distribution. Genome Biol. (2017) 18:19. doi: 10.1186/s13059-016-1131-9 28129774 PMC5273857

[13] Galanter JM Gignoux CR Oh SS Torgerson D Pino-Yanes M Thakur N. Differential methylation between ethnic sub-groups reflects the effect of genetic ancestry and environmental exposures. Elife. (2017) 6. doi: 10.7554/eLife.20532 PMC5207770 28044981

[14] Bonder MJ Luijk R Zhernakova DV Moed M Deelen P Vermaat M. Disease variants alter transcription factor levels and methylation of their binding sites. Nat Genet. (2017) 49:131–8. doi: 10.1038/ng.3721 27918535

[15] Szklarczyk D Gable AL Lyon D Junge A Wyder S Huerta-Cepas J. STRING v11: protein-protein association networks with increased coverage, supporting functional discovery in genome-wide experimental datasets. Nucleic Acids Res. (2019) 47:D607–d613. doi: 10.1093/nar/gky1131 30476243 PMC6323986

[16] Bzdok D Dunbar RIM. Social isolation and the brain in the pandemic era. Nat Hum Behav. (2022) 6:1333–43. doi: 10.1038/s41562-022-01453-0 36258130

[17] Mentzer AJ O’Connor D Bibi S Chelysheva I Clutterbuck EA Demissie T. Human leukocyte antigen alleles associate with COVID-19 vaccine immunogenicity and risk of breakthrough infection. Nat Med. (2023) 29:147–57. doi: 10.1038/s41591-022-02078-6 PMC9873562 36228659

[18] Moynihan R Sanders S Michaleff ZA Scott AM Clark J To EJ. Impact of COVID-19 pandemic on utilisation of healthcare services: a systematic review. BMJ Open. (2021) 11:e045343. doi: 10.1136/bmjopen-2020-045343 PMC7969768 33727273

[19] Fioravanti G Bocci Benucci S Prostamo A Banchi V Casale S. Effects of the COVID-19 pandemic on psychological health in a sample of Italian adults: A three-wave longitudinal study. Psychiatry Res. (2022) 315:114705. doi: 10.1016/j.psychres.2022.114705 35809495 PMC9250412

[20] Domínguez-Andrés J Netea MG. Impact of historic migrations and evolutionary processes on human immunity. Trends Immunol. (2019) 40:1105–19. doi: 10.1016/j.it.2019.10.001 PMC7106516 31786023

[21] Fiebiger U Bereswill S Heimesaat MM. Dissecting the interplay between intestinal microbiota and host immunity in health and disease: lessons learned from germfree and gnotobiotic animal models. Eur J Microbiol Immunol (Bp). (2016) 6:253–71. doi: 10.1556/1886.2016.00036 PMC5146645 27980855

[22] Round JL Mazmanian SK. The gut microbiota shapes intestinal immune responses during health and disease. Nat Rev Immunol. (2009) 9:313–23. doi: 10.1038/nri2515 PMC4095778 19343057

[23] Strachan DP. Hay fever, hygiene, and household size. Bmj. (1989) 299:1259–60. doi: 10.1136/bmj.299.6710.1259 PMC1838109 2513902

[24] Bach JF. The hygiene hypothesis in autoimmunity: the role of pathogens and commensals. Nat Rev Immunol. (2018) 18:105–20. doi: 10.1038/nri.2017.111 29034905

[25] Bach JF. The effect of infections on susceptibility to autoimmune and allergic diseases. N Engl J Med. (2002) 347:911–20. doi: 10.1056/NEJMra020100 12239261

[26] Arunachalam PS Scott MKD Hagan T Li C Feng Y Wimmers F. Systems vaccinology of the BNT162b2 mRNA vaccine in humans. Nature. (2021) 596:410–6. doi: 10.1038/s41586-021-03791-x PMC8761119 34252919

[27] Li C Lee A Grigoryan L Arunachalam PS Scott MKD Trisal M. Mechanisms of innate and adaptive immunity to the Pfizer-BioNTech BNT162b2 vaccine. Nat Immunol. (2022) 23:543–55. doi: 10.1038/s41590-022-01163-9 PMC8989677 35288714

[28] Murphy DM Cox DJ Connolly SA Breen EP Brugman AA Phelan JJ. Trained immunity is induced in humans after immunization with an adenoviral vector COVID-19 vaccine. J Clin Invest. (2023) 133. doi: 10.1172/JCI162581 PMC9843058 36282571

[29] Ndeupen S Qin Z Jacobsen S Bouteau A Estanbouli H Igyártó BZ. The mRNA-LNP platform’s lipid nanoparticle component used in preclinical vaccine studies is highly inflammatory. iScience. (2021) 24:103479. doi: 10.1016/j.isci.2021.103479 34841223 PMC8604799

[30] Qin Z Bouteau A Herbst C Igyártó BZ. Pre-exposure to mRNA-LNP inhibits adaptive immune responses and alters innate immune fitness in an inheritable fashion. PloS Pathog. (2022) 18:e1010830. doi: 10.1371/journal.ppat.1010830 36054264 PMC9477420

[31] Föhse K Geckin B Zoodsma M Kilic G Liu Z Röring RJ. The impact of BNT162b2 mRNA vaccine on adaptive and innate immune responses. medRxiv. (2023) 2021:2005.2003.21256520. doi: 10.1016/j.clim.2023.109762 37673225

[32] Moore LD Le T Fan G. DNA methylation and its basic function. Neuropsychopharmacology. (2013) 38:23–38. doi: 10.1038/npp.2012.112 22781841 PMC3521964

[33] Joubert BR Felix JF Yousefi P Bakulski KM Just AC Breton C. DNA methylation in newborns and maternal smoking in pregnancy: genome-wide consortium meta-analysis. Am J Hum Genet. (2016) 98:680–96. doi: 10.1016/j.ajhg.2016.02.019 PMC4833289 27040690

[34] Gruzieva O Xu CJ Breton CV Annesi-Maesano I Antó JM Auffray C. Epigenome-wide meta-analysis of methylation in children related to prenatal NO2 air pollution exposure. Environ Health Perspect. (2017) 125:104–10. doi: 10.1289/EHP36 PMC5226705 27448387

[35] Xu CJ Söderhäll C Bustamante M Baïz N Gruzieva O Gehring U. DNA methylation in childhood asthma: an epigenome-wide meta-analysis. Lancet Respir Med. (2018) 6:379–88. doi: 10.1016/S2213-2600(18)30052-3 29496485

[36] van der Heijden WA Van de Wijer L Keramati F Trypsteen W Rutsaert S Horst RT. Chronic HIV infection induces transcriptional and functional reprogramming of innate immune cells. JCI Insight. (2021) 6. doi: 10.1172/jci.insight.145928 PMC8119206 33630761

[37] Kass L Desai T Sullivan K Muniz D Wells A. Changes to physical activity, sitting time, eating behaviours and barriers to exercise during the first COVID-19 ‘Lockdown’ in an english cohort. Int J Environ Res Public Health. (2021) 18. doi: 10.3390/ijerph181910025 PMC8508153 34639327

[38] Bennett G Young E Butler I Coe S. The impact of lockdown during the COVID-19 outbreak on dietary habits in various population groups: A scoping review. Front Nutr. (2021) 8:626432. doi: 10.3389/fnut.2021.626432 33748175 PMC7969646

[39] Geckin B Zoodsma M Kilic G Debisarun PA Rakshit S Adiga V. Differences in immune responses in individuals of Indian and european origin: relevance for the COVID-19 pandemic. Microbiol Spectr. (2023) 11:e0023123. doi: 10.1128/spectrum.00231-23 36779734 PMC10100912

[40] Assarsson E Lundberg M Holmquist G Björkesten J Thorsen SB Ekman D. Homogenous 96-plex PEA immunoassay exhibiting high sensitivity, specificity, and excellent scalability. PloS One. (2014) 9:e95192. doi: 10.1371/journal.pone.0095192 24755770 PMC3995906

[41] Ter Horst R Jaeger M Smeekens SP Oosting M Swertz MA Li Y. Host and environmental factors influencing individual human cytokine responses. Cell. (2016) 167:1111–1124.e1113. doi: 10.1016/j.cell.2016.10.018 27814508 PMC5787854

[42] Ritchie ME Phipson B Wu D Hu Y Law CW Shi W. limma powers differential expression analyses for RNA-sequencing and microarray studies. Nucleic Acids Res. (2015) 43:e47. doi: 10.1093/nar/gkv007 25605792 PMC4402510

[43] Aryee MJ Jaffe AE Corrada-Bravo H Ladd-Acosta C Feinberg AP Hansen KD. Minfi: a flexible and comprehensive Bioconductor package for the analysis of Infinium DNA methylation microarrays. Bioinformatics. (2014) 30:1363–9. doi: 10.1093/bioinformatics/btu049 PMC4016708 24478339

[44] Liu Z Kilic G Li W Bulut O Gupta MK Zhang B. Multi-omics integration reveals only minor long-term molecular and functional sequelae in immune cells of individuals recovered from COVID-19. Front Immunol. (2022) 13:838132. doi: 10.3389/fimmu.2022.838132 35464396 PMC9022455

[45] Pidsley R Zotenko E Peters TJ Lawrence MG Risbridger GP Molloy P. Critical evaluation of the Illumina MethylationEPIC BeadChip microarray for whole-genome DNA methylation profiling. Genome Biol. (2016) 17:208. doi: 10.1186/s13059-016-1066-1 27717381 PMC5055731

[46] Touleimat N Tost J. Complete pipeline for Infinium(^®^) Human Methylation 450K BeadChip data processing using subset quantile normalization for accurate DNA methylation estimation. Epigenomics. (2012) 4:325–41. doi: 10.2217/epi.12.21 22690668

[47] Salas LA Koestler DC Butler RA Hansen HM Wiencke JK Kelsey KT. An optimized library for reference-based deconvolution of whole-blood biospecimens assayed using the Illumina HumanMethylationEPIC BeadArray. Genome Biol. (2018) 19:64. doi: 10.1186/s13059-018-1448-7 29843789 PMC5975716

[48] Leek JT Storey JD. Capturing heterogeneity in gene expression studies by surrogate variable analysis. PloS Genet. (2007) 3:1724–35. doi: 10.1371/journal.pgen.0030161 PMC1994707 17907809

[49] Willer CJ Li Y Abecasis GR. METAL: fast and efficient meta-analysis of genomewide association scans. Bioinformatics. (2010) 26:2190–1. doi: 10.1093/bioinformatics/btq340 PMC2922887 20616382

[50] Wu T Hu E Xu S Chen M Guo P Dai Z. clusterProfiler 4.0: A universal enrichment tool for interpreting omics data. Innovation (Camb). (2021) 2:100141. doi: 10.1016/j.xinn.2021.100141 34557778 PMC8454663

[51] Szklarczyk D Kirsch R Koutrouli M Nastou K Mehryary F Hachilif R. The STRING database in 2023: protein-protein association networks and functional enrichment analyses for any sequenced genome of interest. Nucleic Acids Res. (2023) 51:D638–d646. doi: 10.1093/nar/gkac1000 36370105 PMC9825434

