# Supplementary material for: Impact of COVID-19, lockdowns and vaccination on immune responses in a HIV cohort in the Netherlands
Source: Front Immunol. 2024 Dec 18;15:1459593. doi: 10.3389/fimmu.2024.1459593 (PMC11688194; doi:10.3389/fimmu.2024.1459593)

**Figure S1 targeted proteomics results**


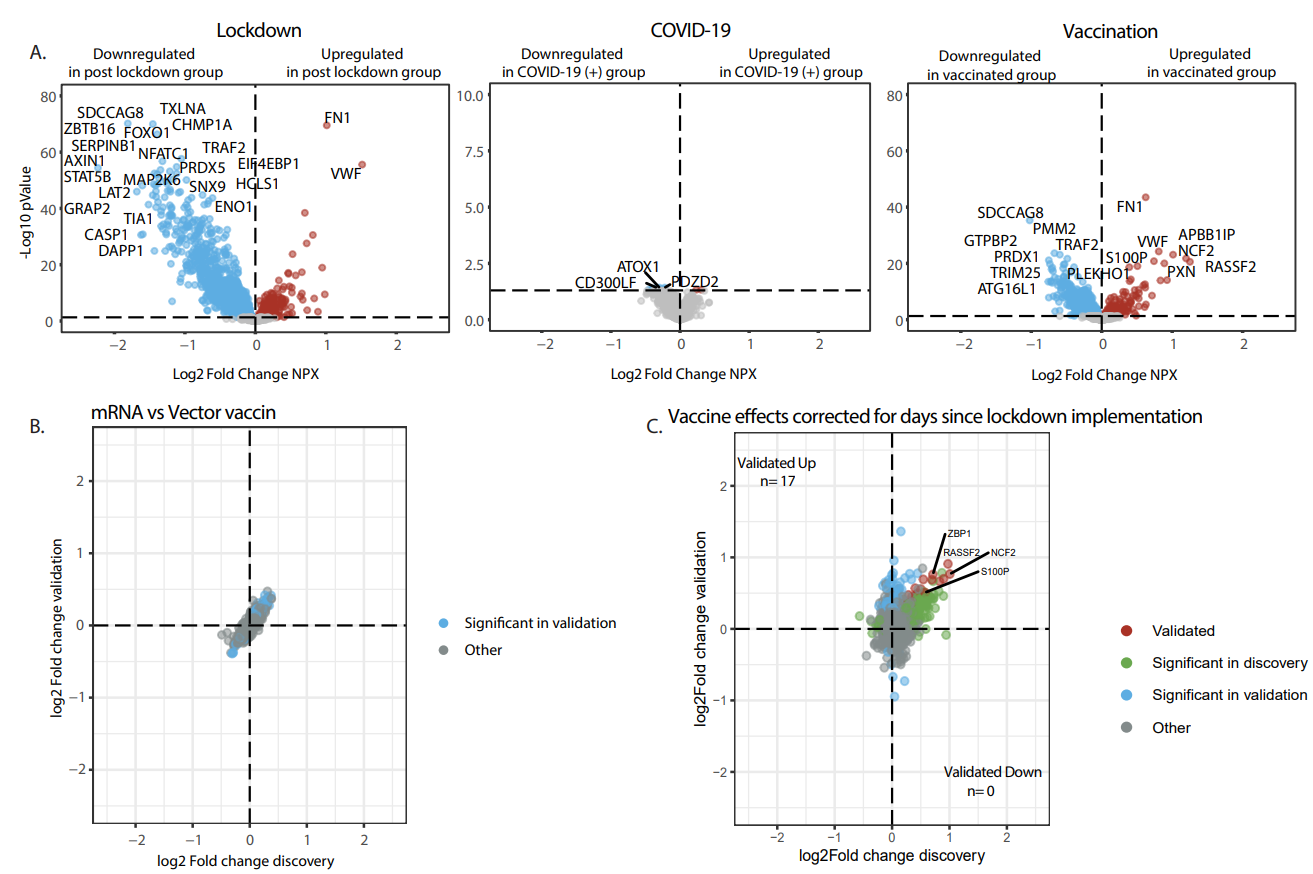


Figure S1: Results from targeted proteomics.

A. Volcano Plots Showing Differential Abundance of Proteins in the Discovery Cohort. X-axis: Log2 Fold Change of Normalized Protein Expression (NXP). Y-axis: -Log10 p-value. Colored dots: p value <0.05. Red dots indicate upregulated proteins, while blue dots represent downregulated proteins. Please note the deviant Y-axis range in COVID-19 plot. Results from linear models adjusted for age, sex, and seasonality. Labeled are the most significantly differentially abundant proteins (DAPs)

B, C. Four Quadrant Scatter Plot Showing Log2 Fold Change in Protein Expression (NXP) in the Discovery Cohort on the X-axis and Validation Cohort on the Y-axis. Green dots represent proteins significant only in the discovery cohort (FDR adj. p < 0.05). Blue dots indicate proteins significant only in the validation cohort (p < 0.05). Red dots indicate proteins significant in the same direction in both cohorts (=validated). B. Results from comparing participants who received mRNA based vaccines versus those who received vector-based vaccins. C. Results from analyzing COVID-19 vaccination effect, while adding days since most recent lockdown implementation as covariate.


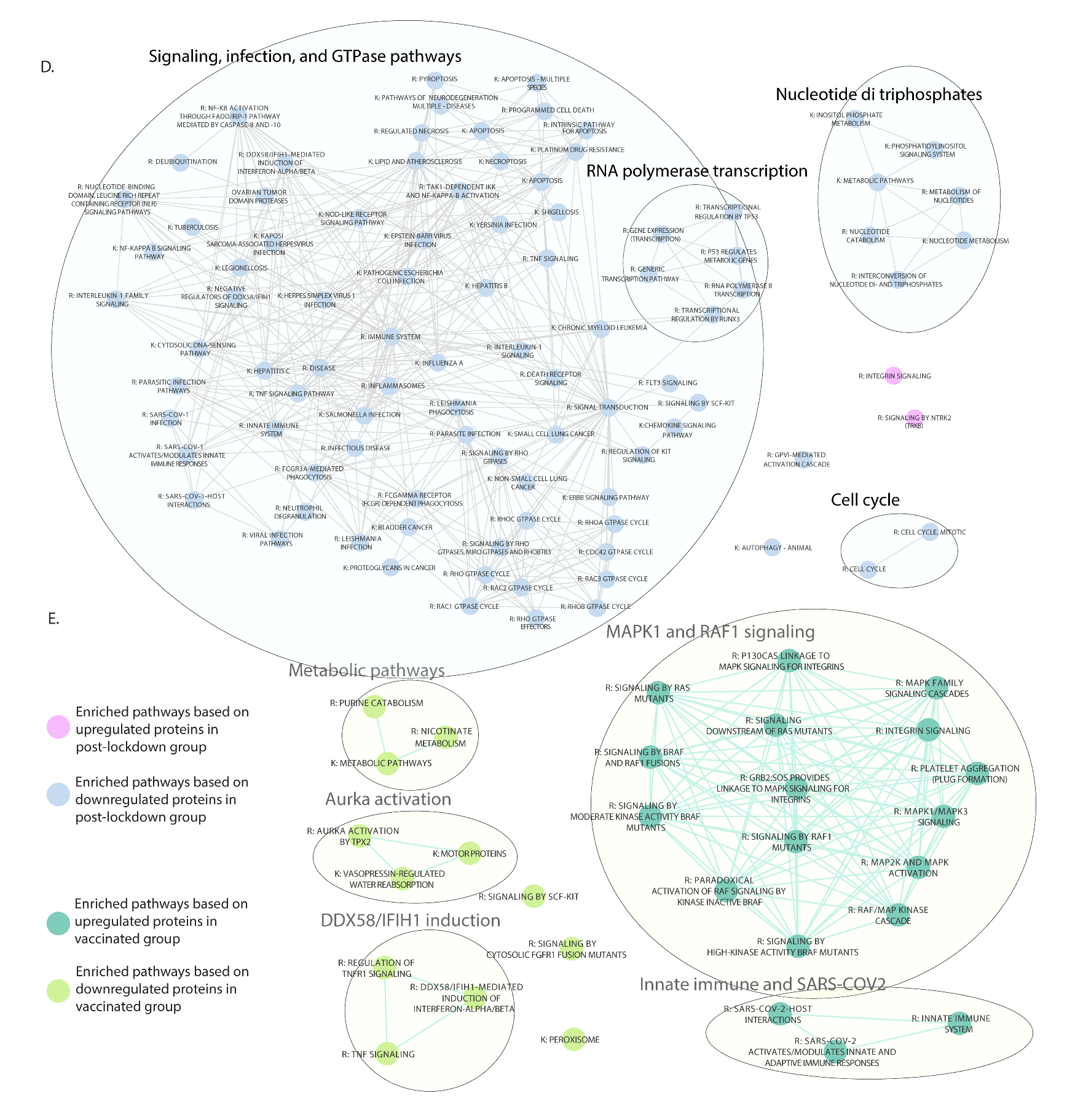


Figure S1 D,E: Results from targeted proteomics.

Pathway Enrichment Analysis results presented as a network for Lockdown (D) and Vaccination(E). Nodes represent pathways and weighted edges represent the degree of gene overlap score between two pathways.

F.

**
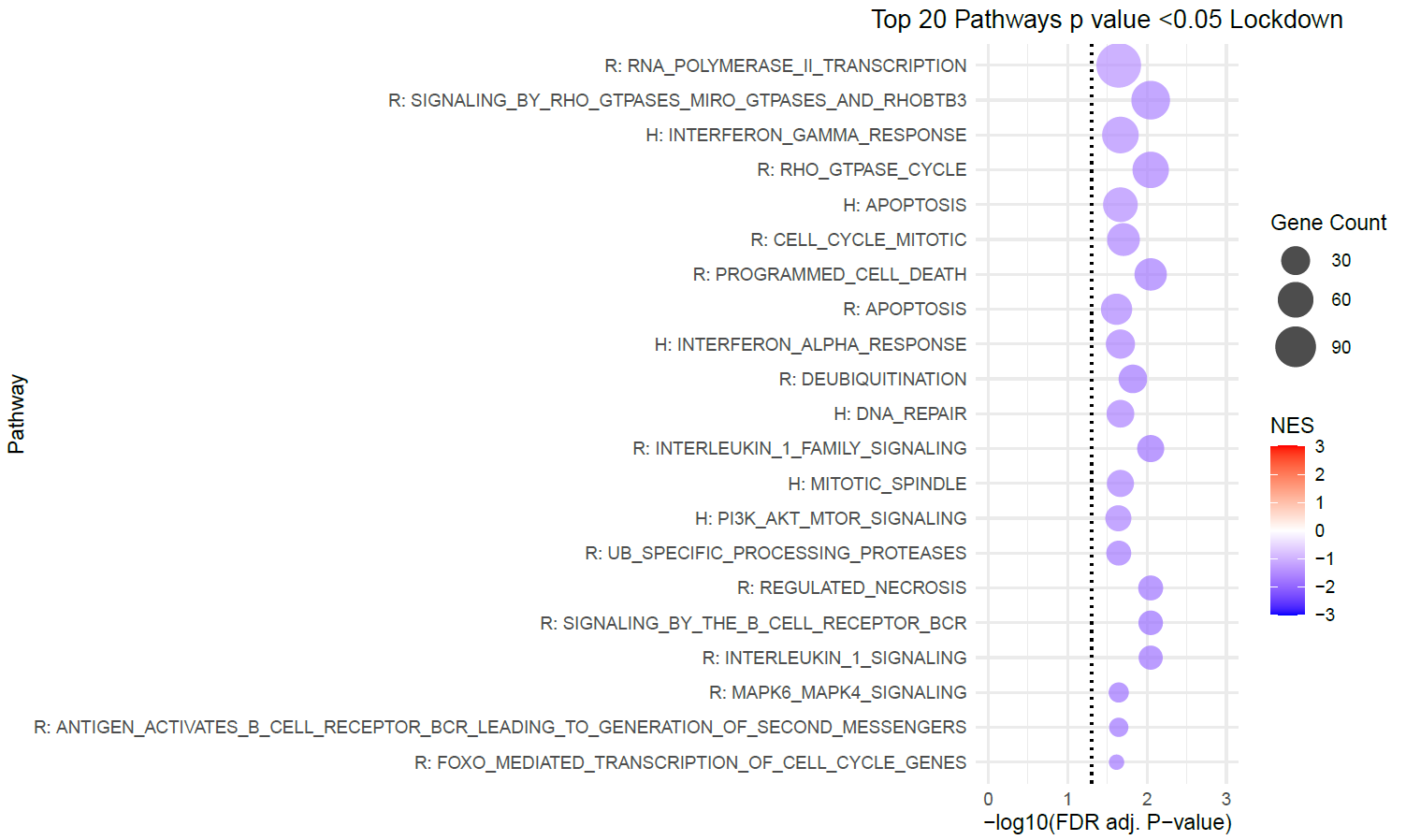
**

G.


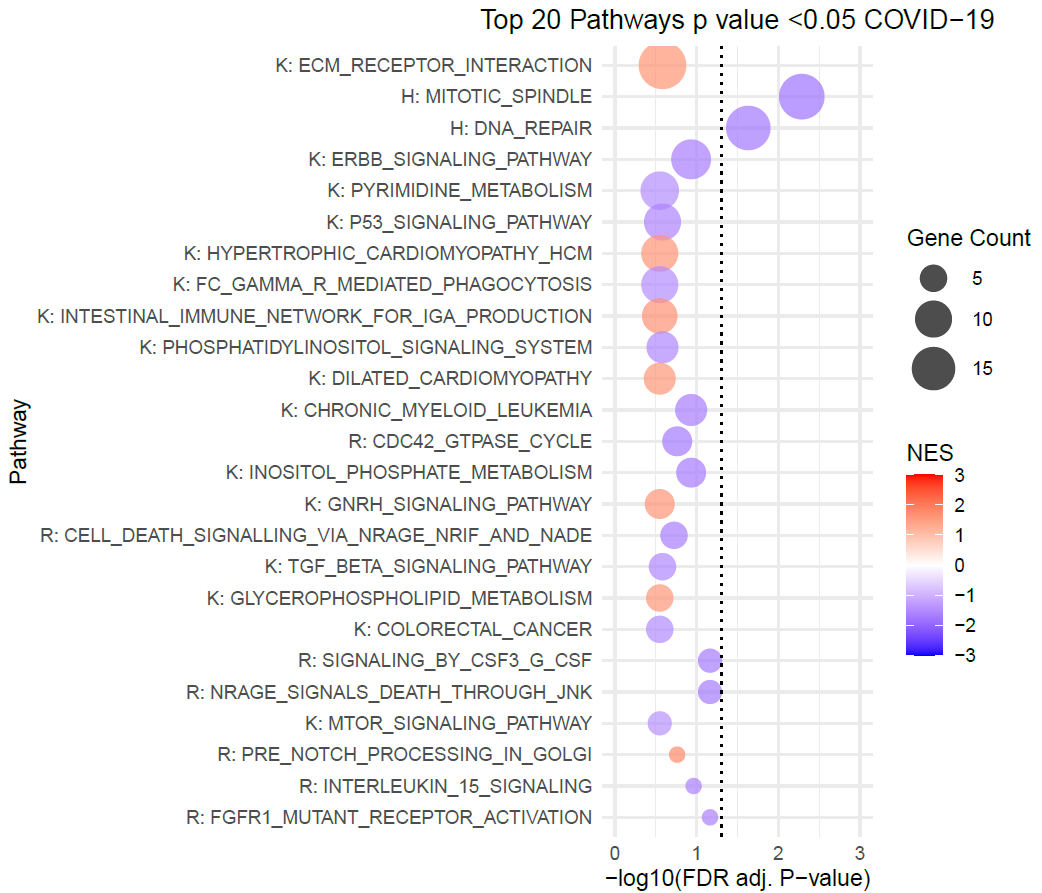

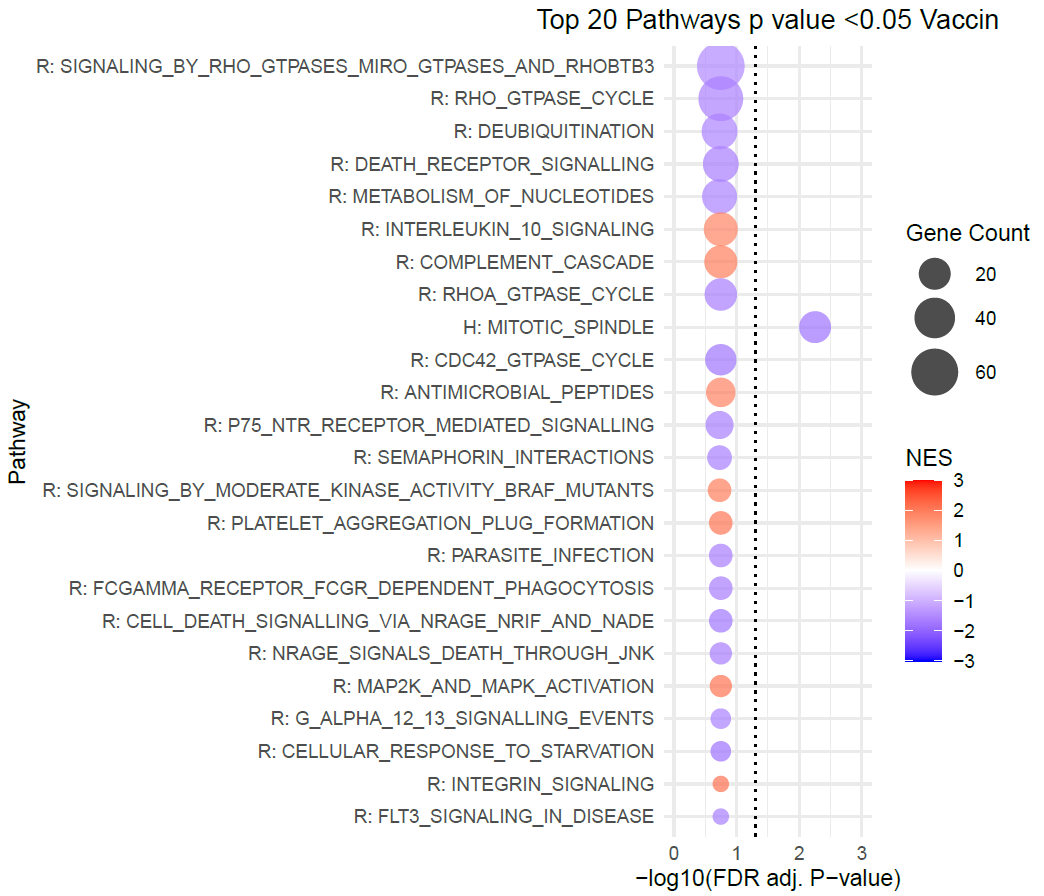
H.

Figure S1 F,E,H: Results from Gene Set Enrichment Analysis (GSEA)

F-H: For each pandemic related group, as a more sensitive approach in addition to pathway enrichment analysis, proteins that were expressed in similar direction in both the validation cohort and the discovery cohort regardless of statistical significance, were assigned a rank based on the t-statistic. GSEA was performed with KEGG, Reactome, and Hallmark reference libraries. Pathways were filtered on nominal P value <0.05, and top 20 pathways based on P value are visualized as a bubble plot. Pathways were ordered on count. Dotted line represents threshold for FDR adjusted p value <0.05; NES: normalized expression score, positive (red) is enriched in proteins with higher expression; negative (blue) lower expression. R: Reactome; K: KEGG; H: Hallmark

**Figure S2 ex-vivo stimulation results**


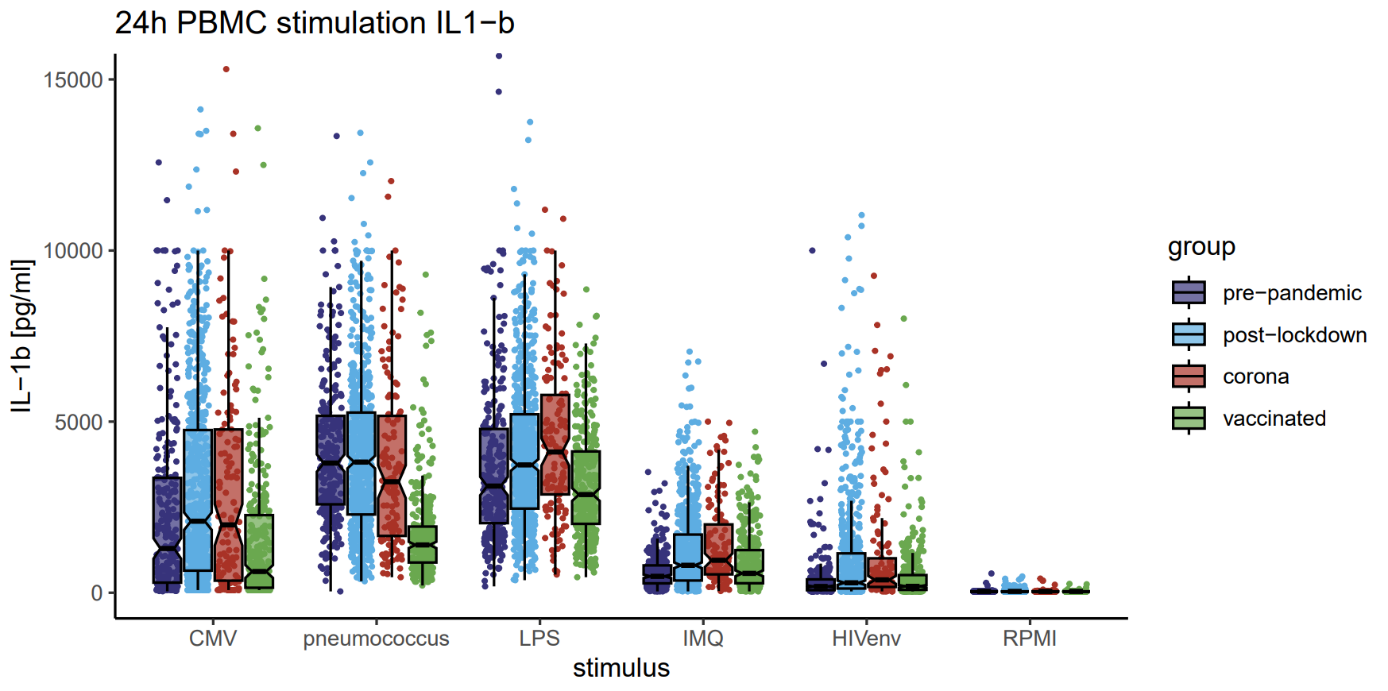
A.


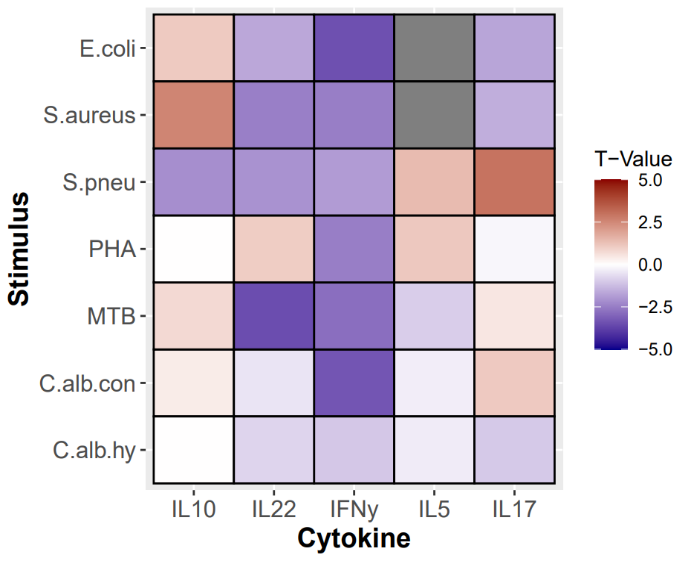


**
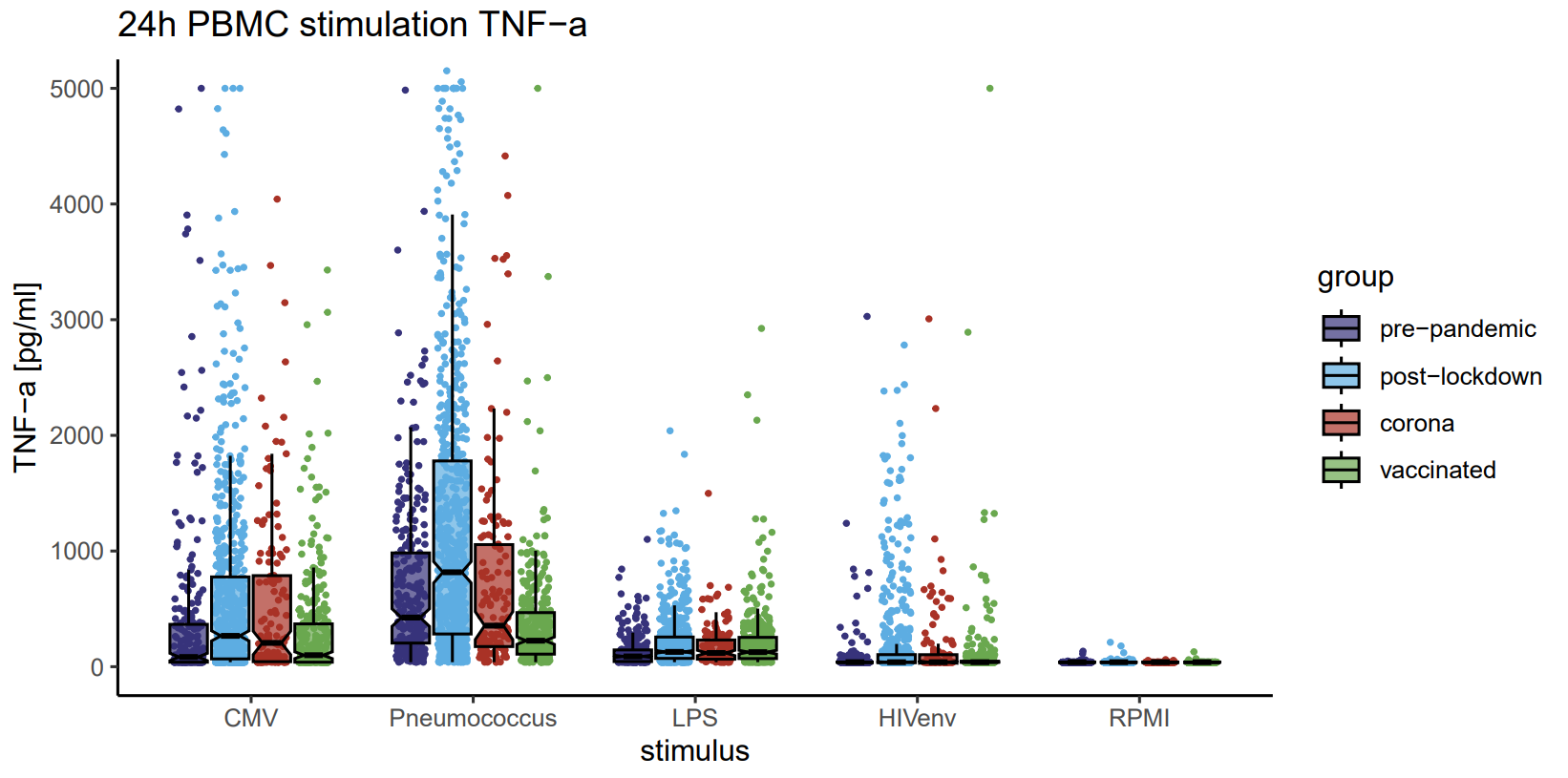
**

B.


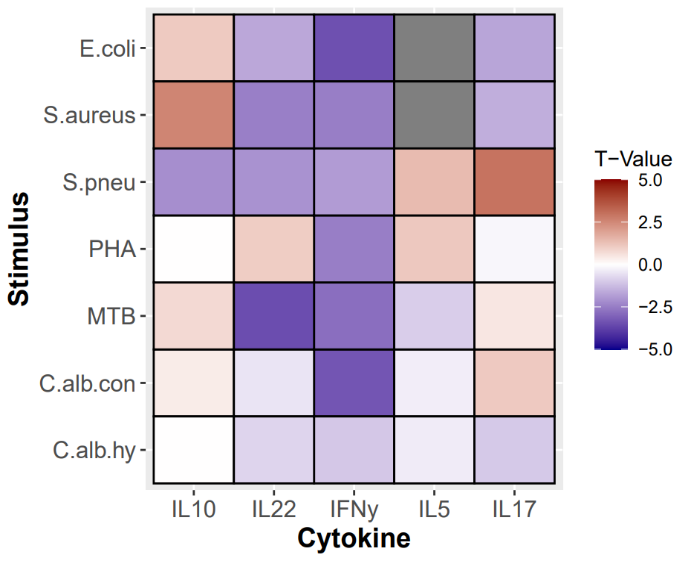


**
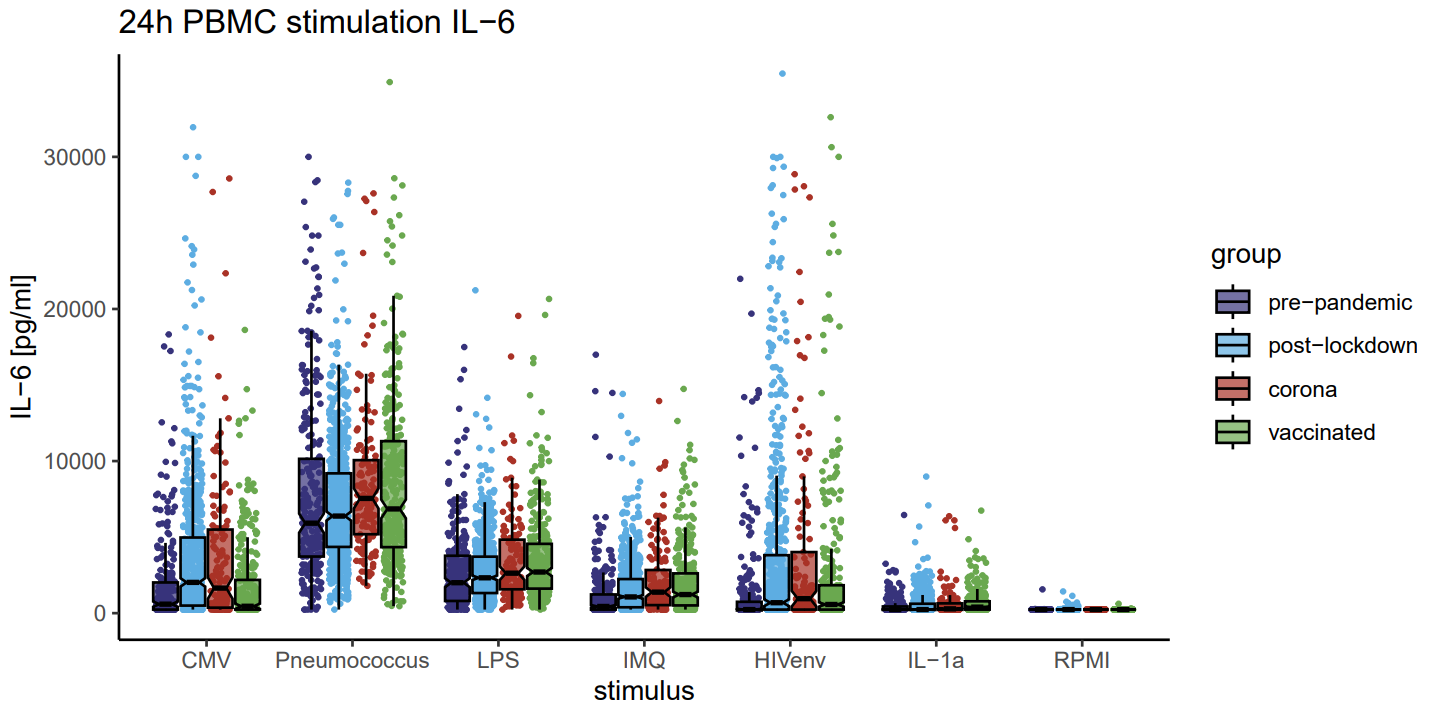
**C.


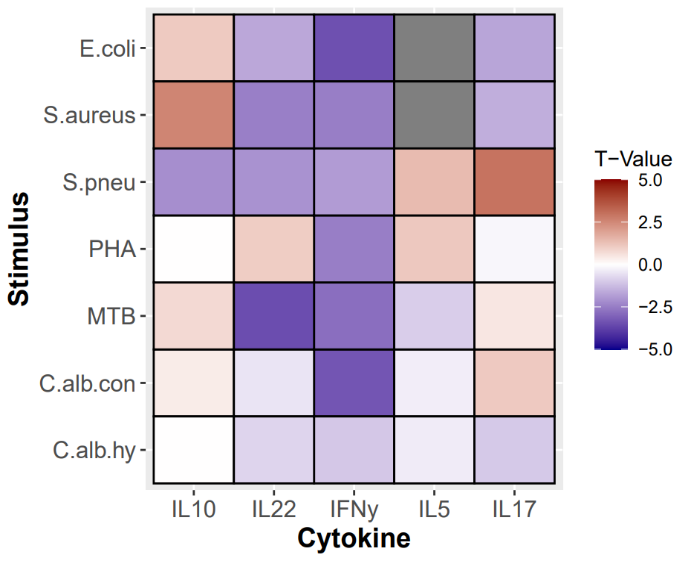


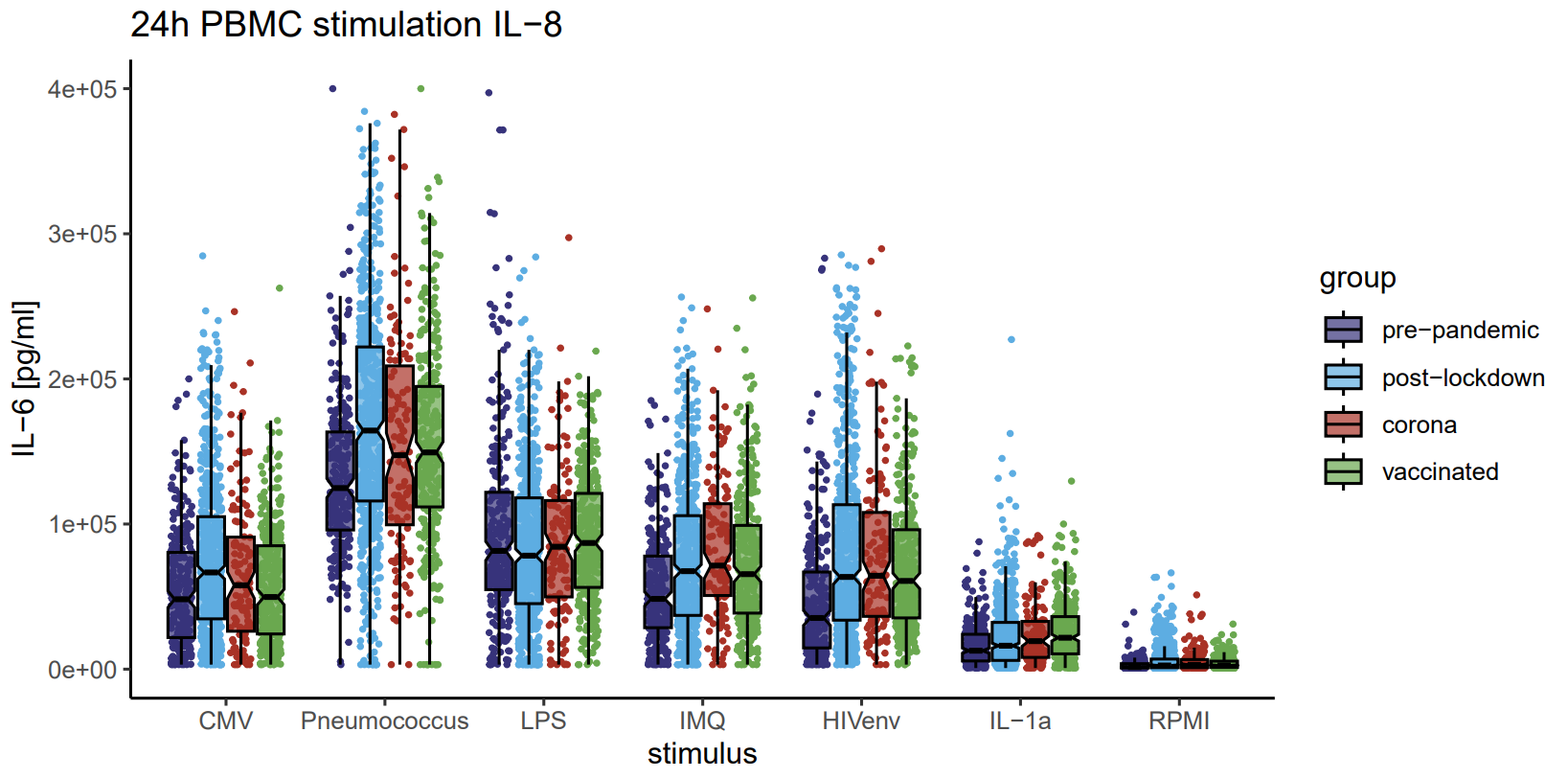
D.


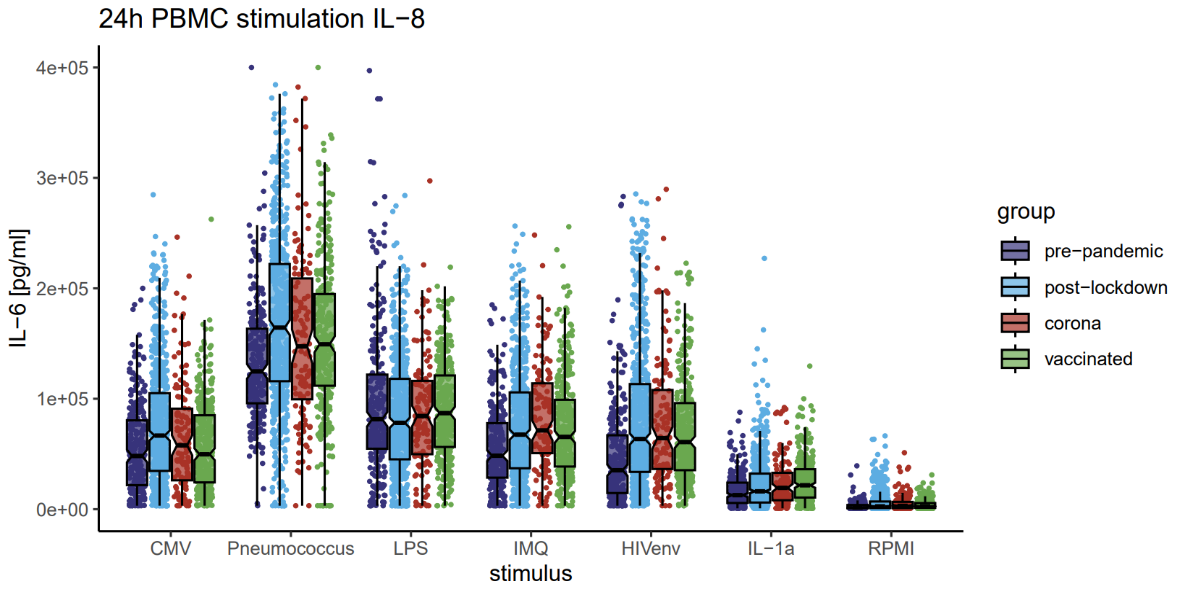


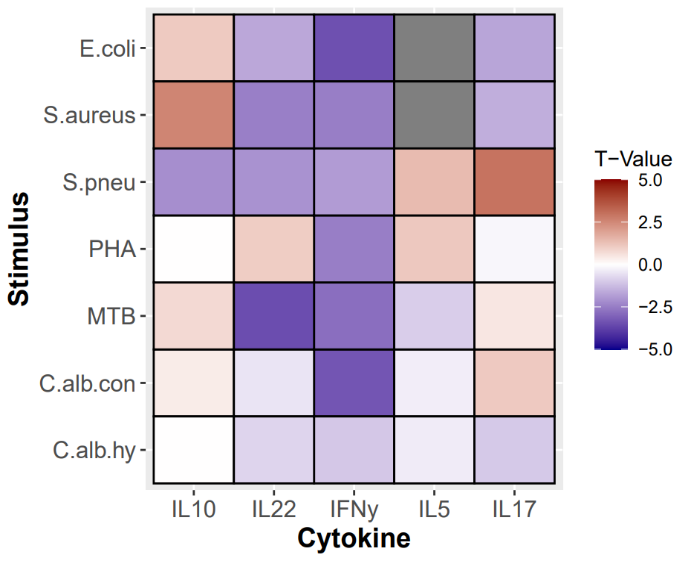


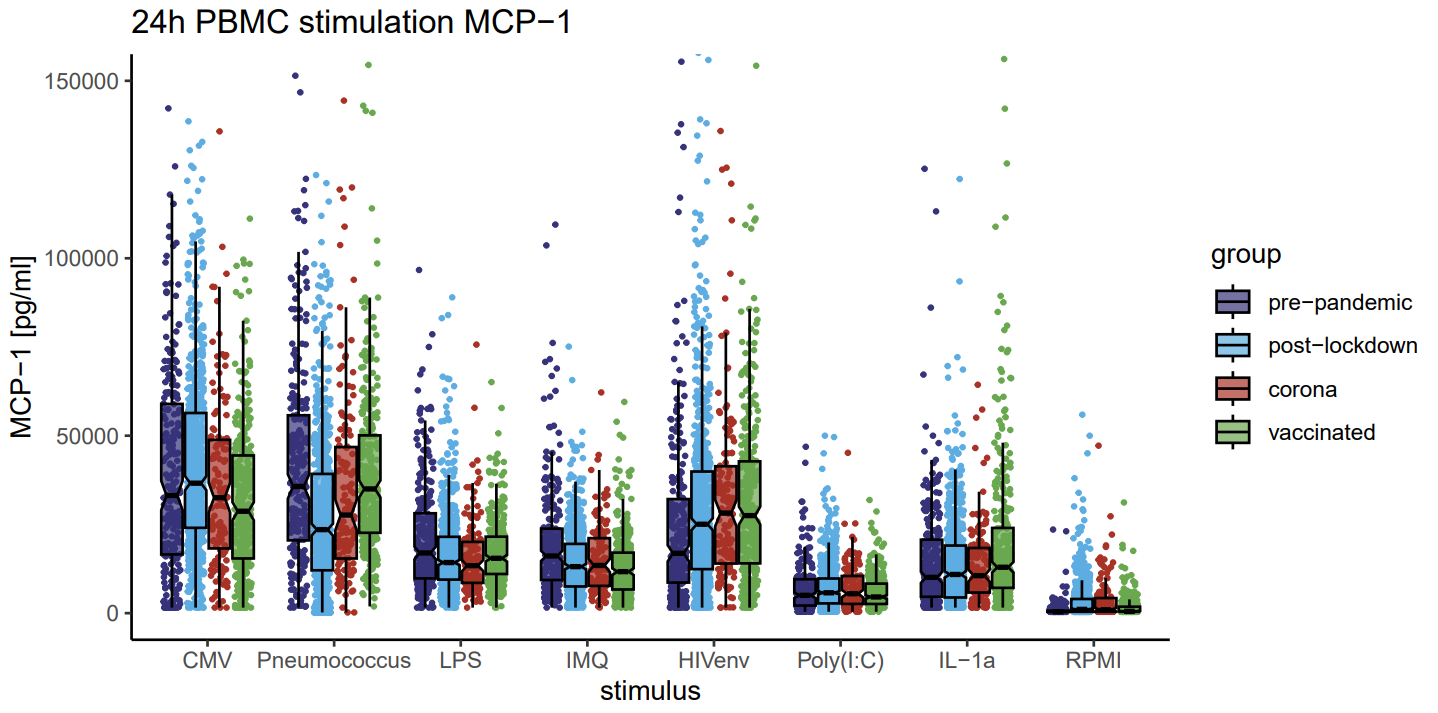
E.


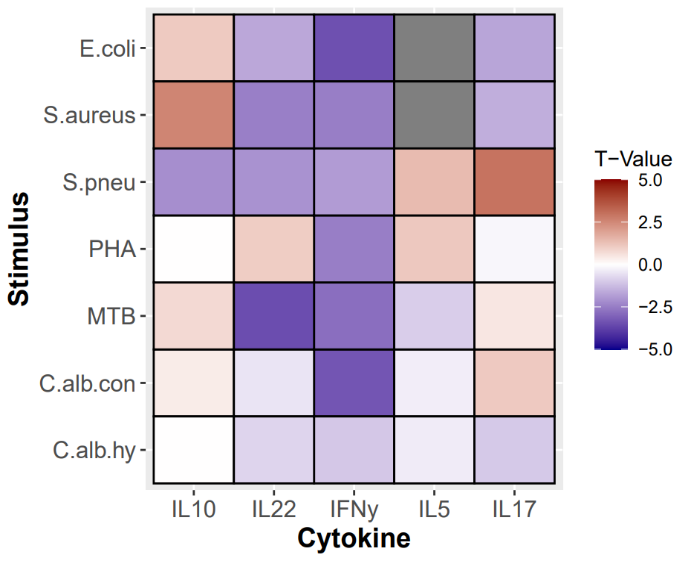


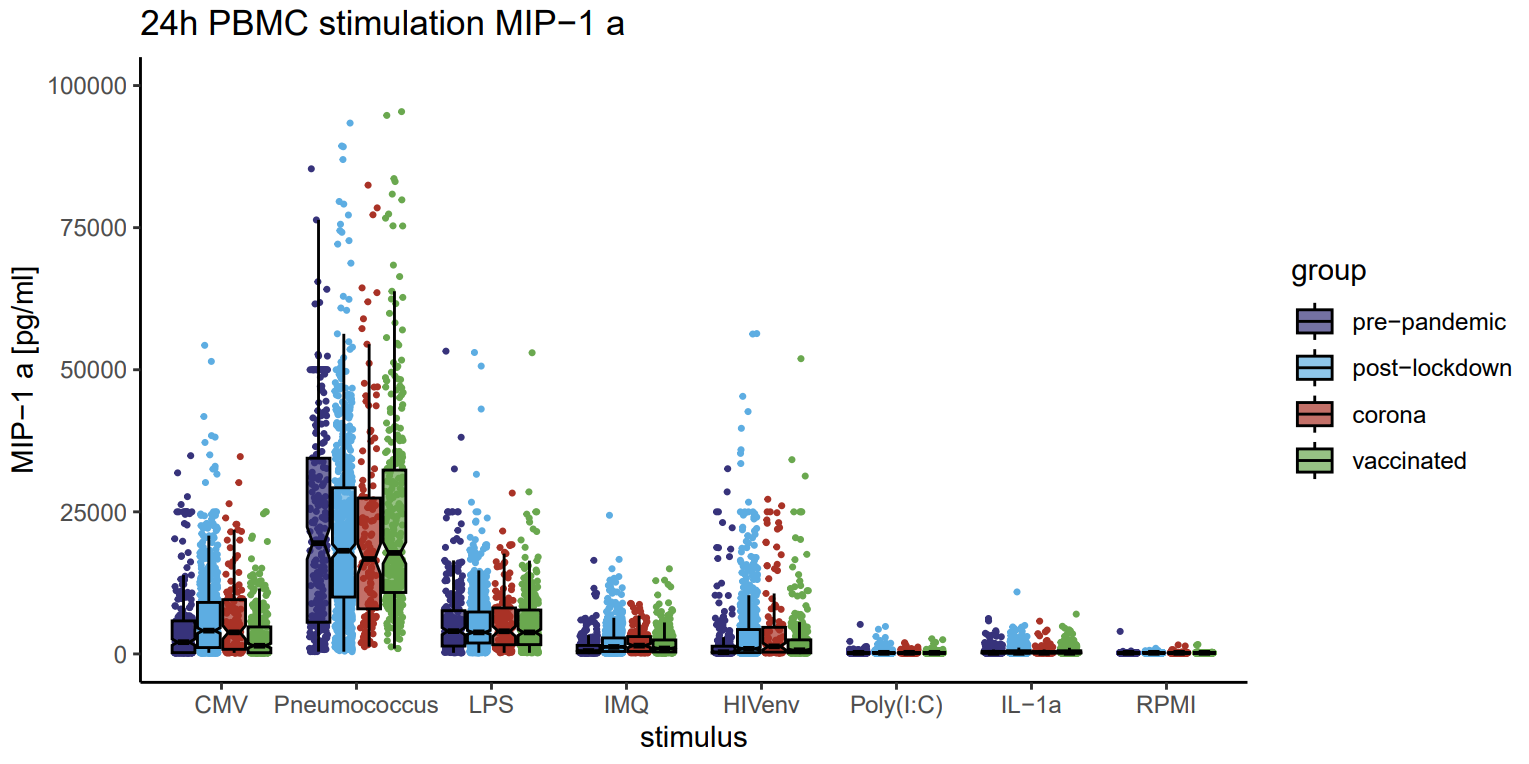
F.


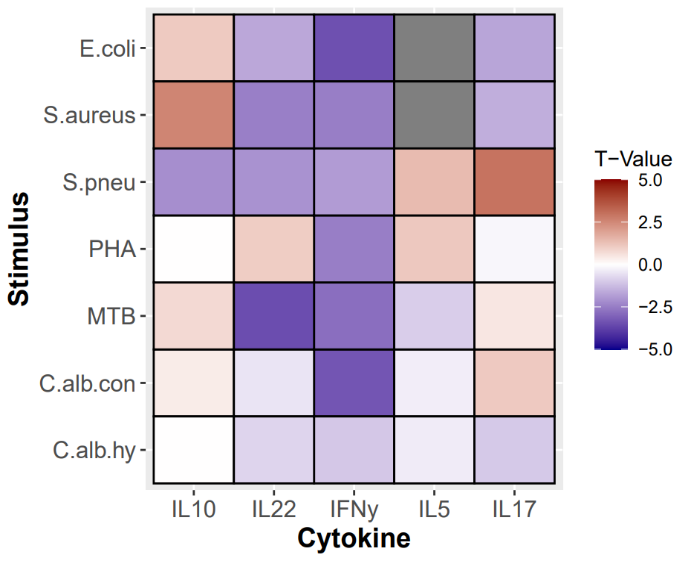


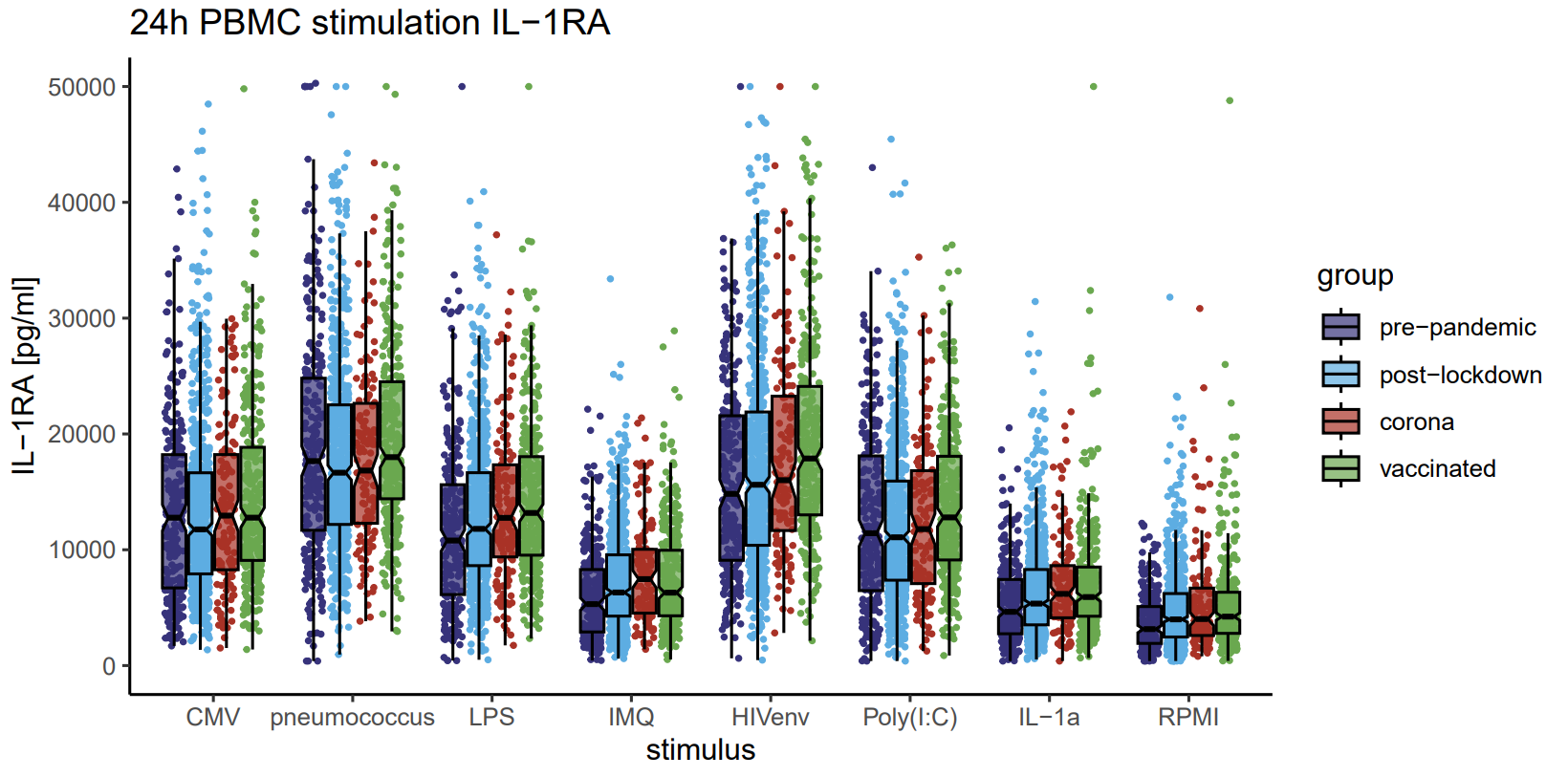
G.


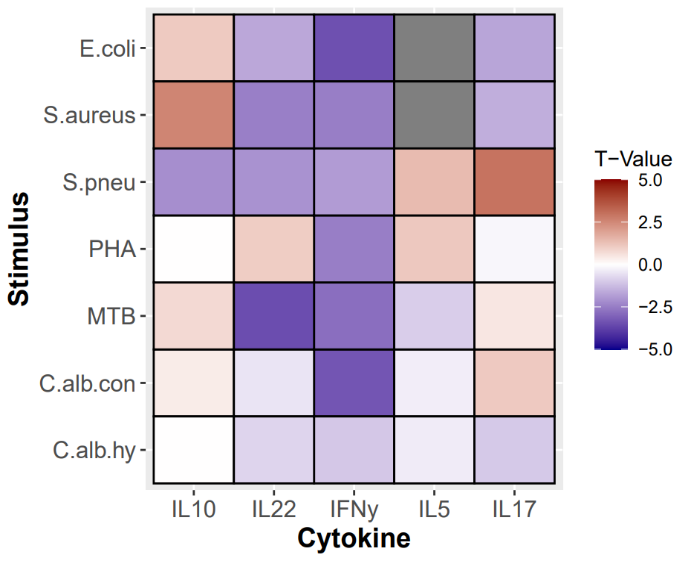


[Trek de aandacht van uw lezer met een veelzeggend citaat uit het document of gebruik deze ruimte om een belangrijk punt te benadrukken. Sleep dit tekstvak als u het ergens anders op de pagina wilt plaatsen.]


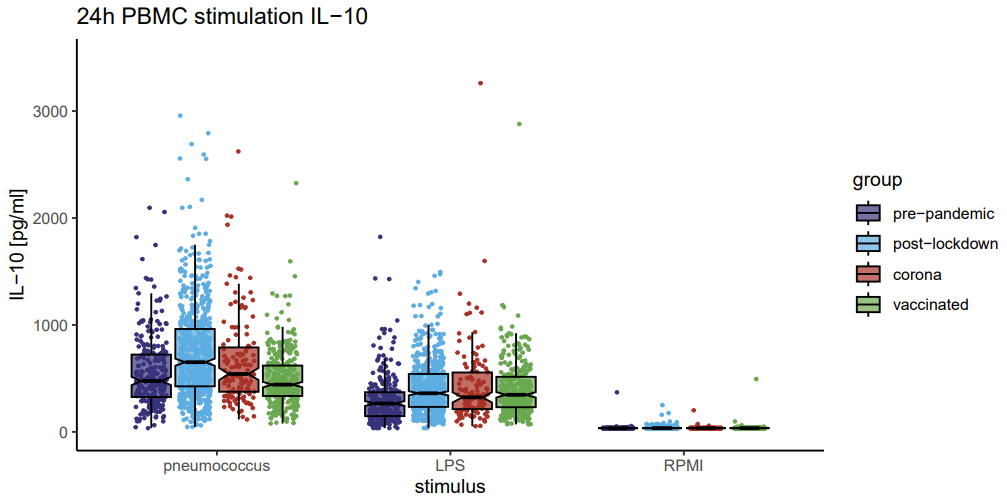


H.


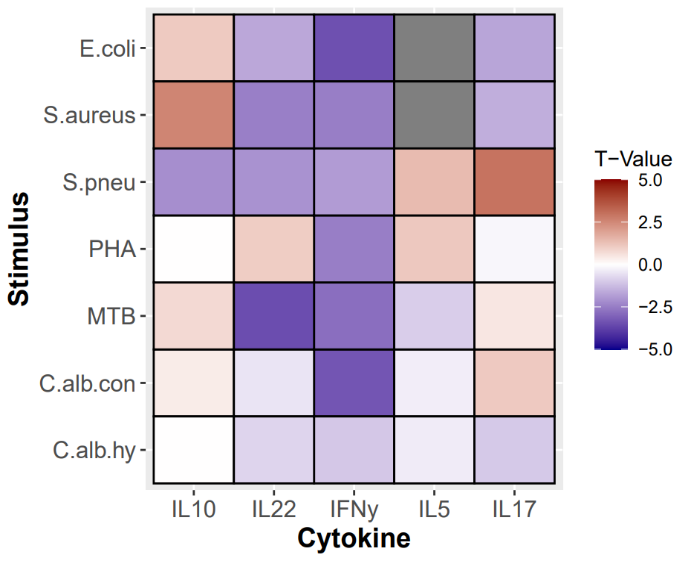


[Trek de aandacht van uw lezer met een veelzeggend citaat uit het document of gebruik deze ruimte om een belangrijk punt te benadrukken. Sleep dit tekstvak als u het ergens anders op de pagina wilt plaatsen.]

Figure S2 A-H: Box Scatter Plots showing untransformed cytokine concentrations in supernatants per group after 24 hour ex-vivo stimulation. Ordered per cytokine. Y-axis: cytokine concentration. X-axis stimuli.

A. IL-1b B. TNF-a C. IL-6 D. IL-8 E. MCP-1 F. MIP-1a G. IL-1RA H. IL-10

Abbreviations: HIVENV: HIV envelope; IMQ: imiquimod; S.Pneu: heat killed S. Pneumoniae; MCP1: monocyte chemoattractant protein-1; MIP1a: macrophage inflammatory protein 1α

I. correction vaccination effects for possible waning lockdown effect.

Adding days since lockdown as a covariate:


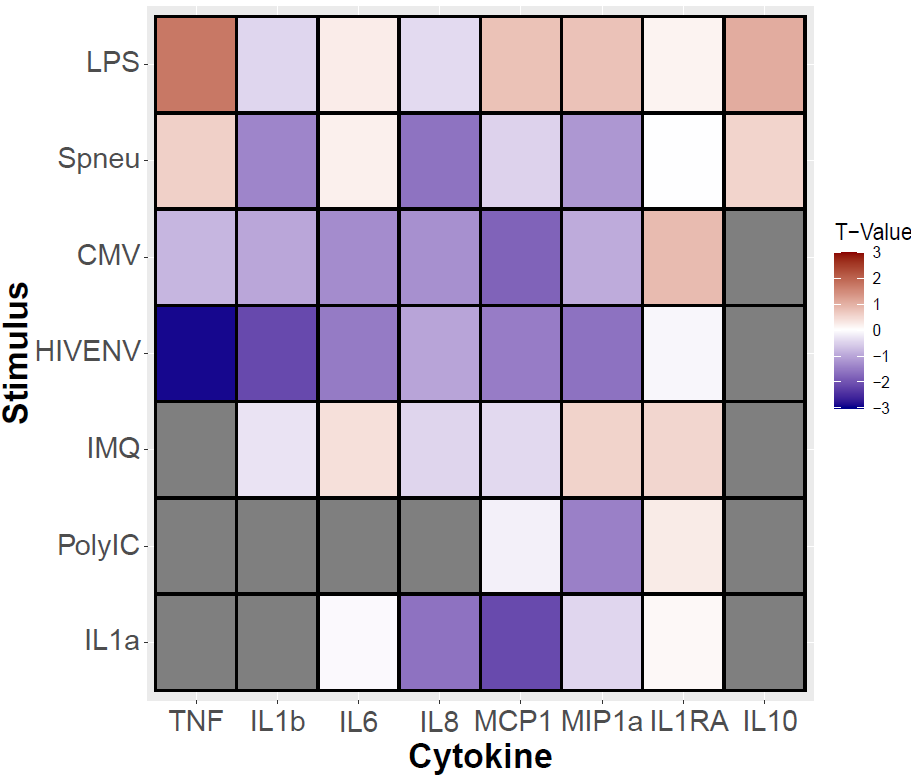

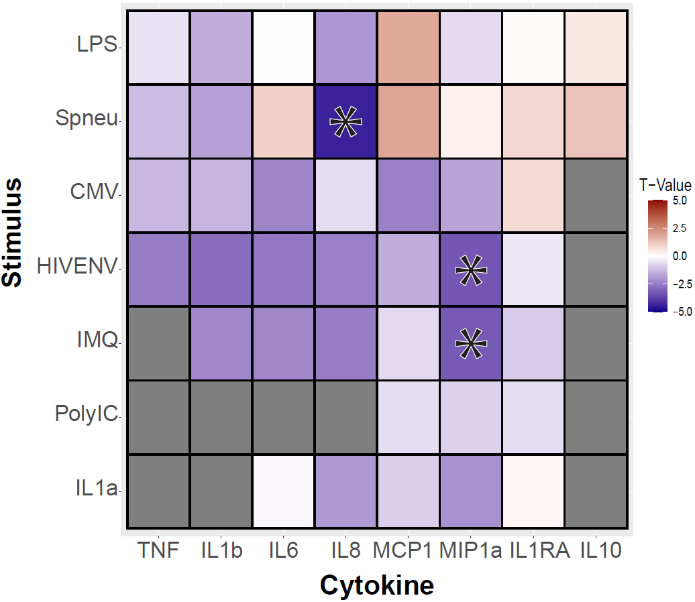
Discovery cohort *= FDR adj p <0.05 Validation cohort * = significant in discovery and p <0.05 in validation

Selecting participants included between 150 and 200 days after lockdown implementation


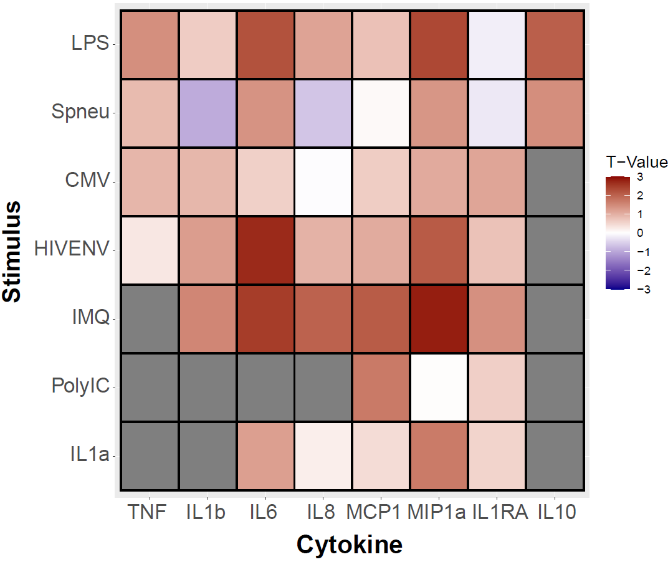

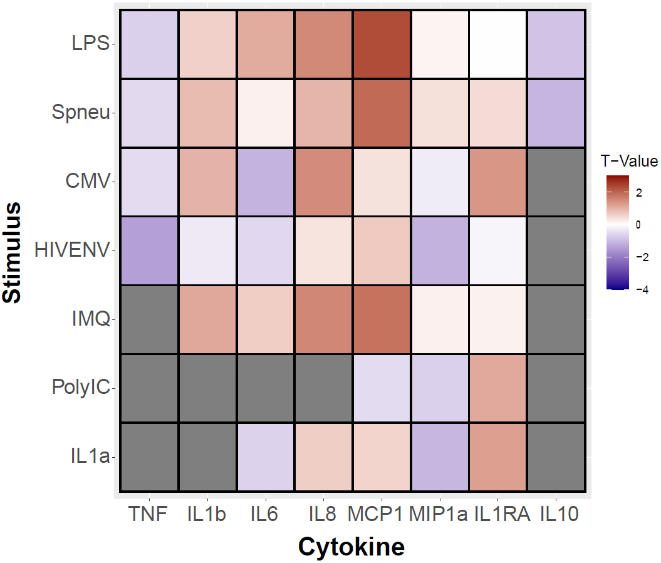
Discovery cohort (n vaccinated = 78; post lockdown n = 110) Validation cohort (n vaccinated = 32 n; post lockdown = 14)

Pre-pandemic group vs vaccinated group, marking with asterisks cytokines only affected by vaccination (not by lockdown)

Discovery cohort: Validation cohort:


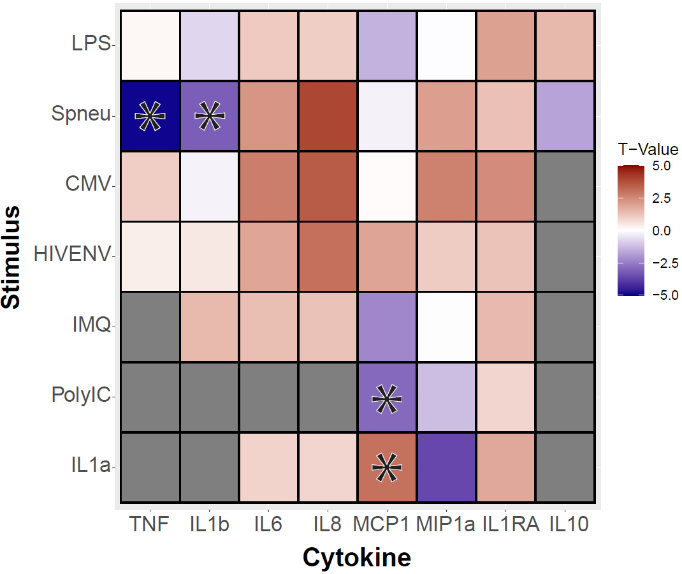
*= FDR adj p <0.05 discovery AND unaffected after lockdown *= significant in discovery and p<0.05 in validation cohort


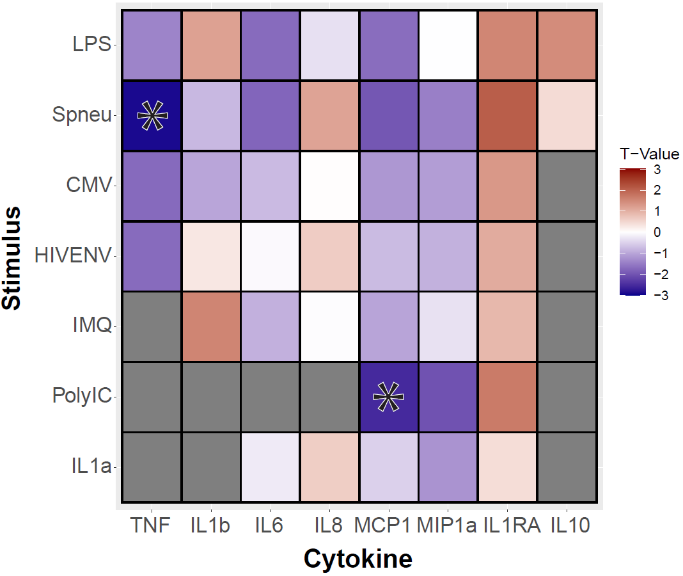


NB: Legend on next page


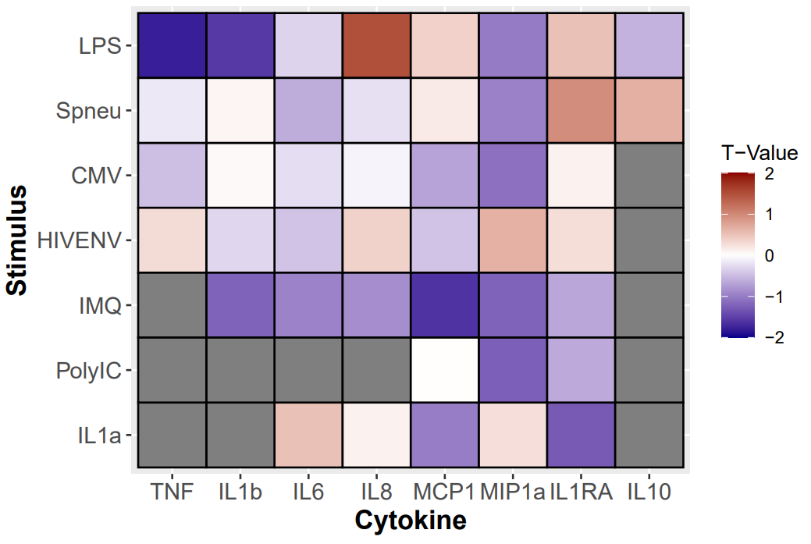
J. mRNA vs Vector Vaccin - Discovery mRNA vs Vector - Validation


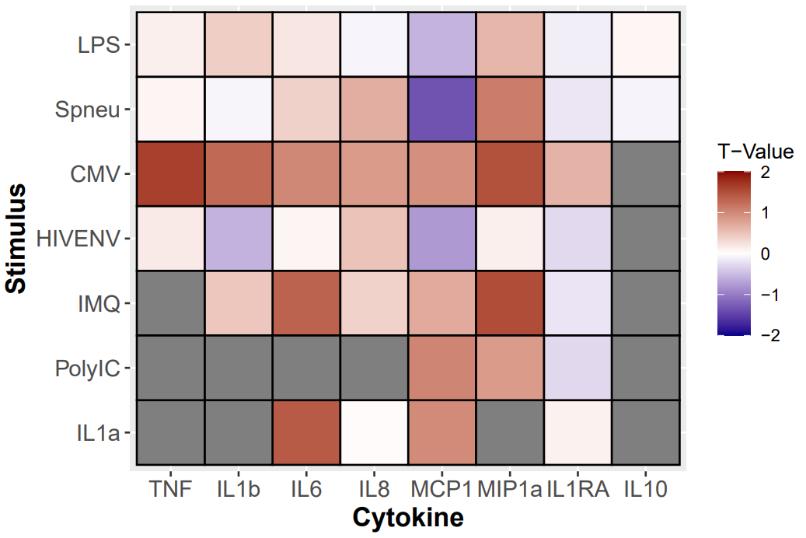


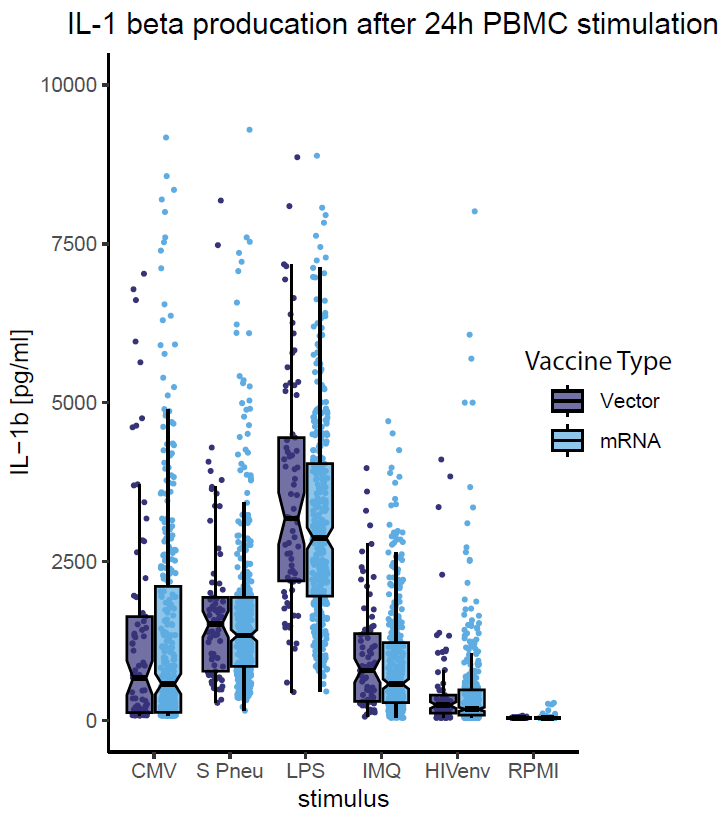
K.

Figure S2 I: Heatmap series displaying methods for investigating possible waning effect of lockdown on the effects of vaccination in the 24 ex-vivo PBMC stimulation experiment. Colors: T values. Blue: lower after vaccination. Red: higher. Upper panels: Results from adding days since most recent lockdown as covariate. Middle panels: results from selecting only participants recruited 150-200 days since lockdown implementation. Lower panels: results from comparing the pre-pandemic group with vaccinated group. Asterisks lower panel indicate cytokines that were not affected or impacted in the opposite direction by the lockdown, but showed significant changes after vaccination compared to the pre-pandemic group.

Figure S2 J-K: Results from comparing participants who received mRNA based vaccines versus those who received vector-based vaccins.

J. Heat Maps of cytokine production after 24-hour ex-vivo stimulation of PBMCs in the discovery and validation cohort of PLWHIV. X-axis: cytokines. Y-axis: stimuli. Colors represent T-values. Red indicates higher cytokine production in mRNA vaccinated group, blue lower. Results from ANCOVA on rank transformed data, adjusted for seasonality and days since most recent lockdown. There were no statistically significant differences.

K. Box Scatter Plot showing untransformed IL-1b concentration in supernatants per vaccine group after 24 hour ex-vivo stimulation. Y-axis: cytokine concentration. X-axis stimuli

L. 7 day stimulation heat maps


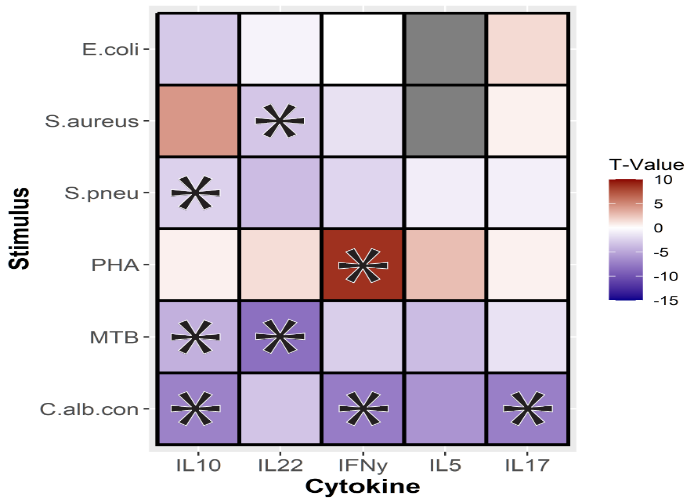


**Lockdown- Discovery Lockdown- Validation**


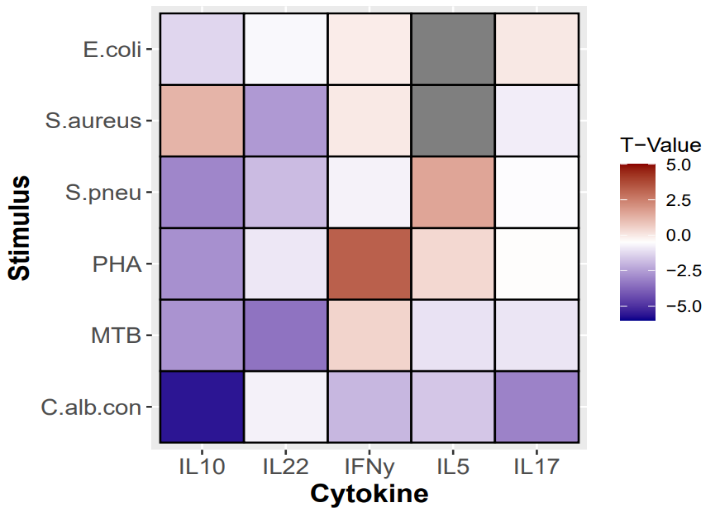


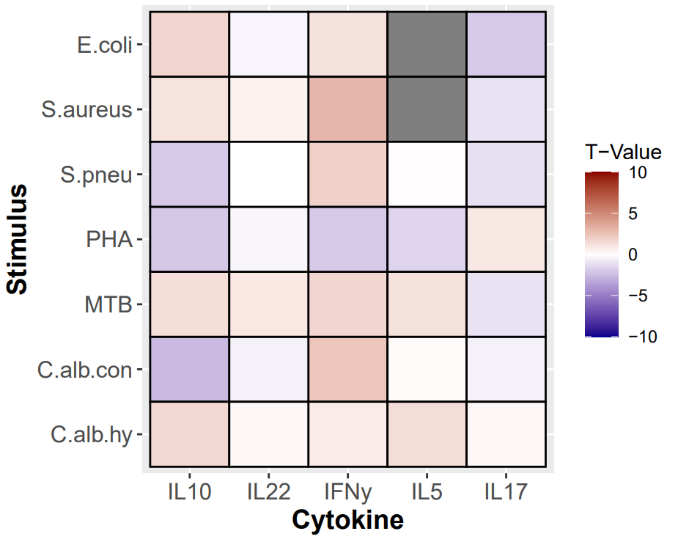

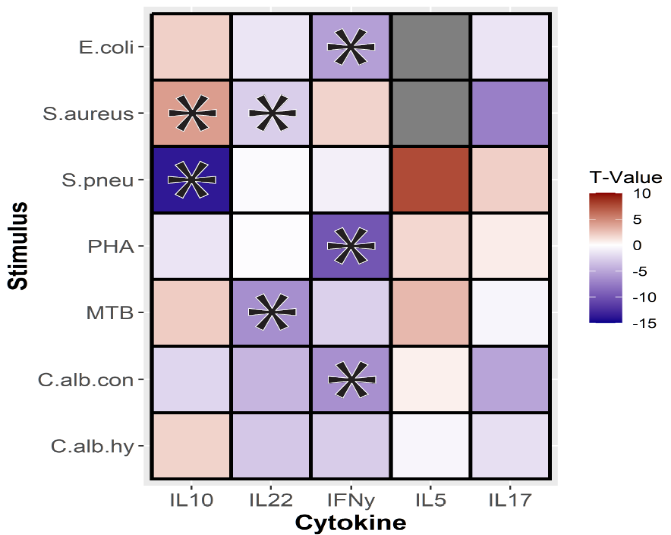

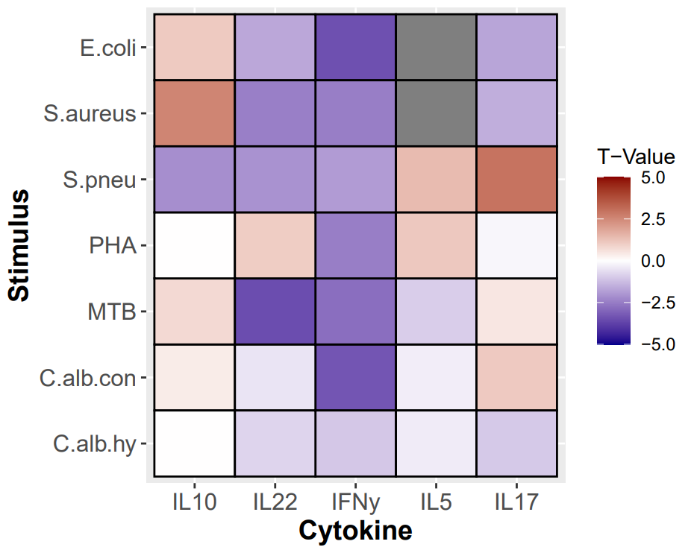

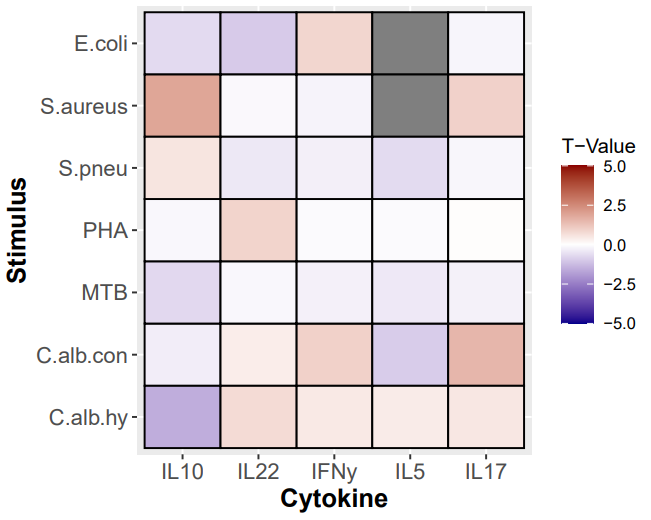


Figure S2 L: Heat Maps of cytokine production after 7-day ex-vivo stimulation of PBMCs in the discovery and validation cohort of PLWHIV showing effects of pandemic. X-axis: cytokines. Y-axis: stimuli. Colors represent T-values. Red indicates higher cytokine production after lockdown, COVID-19 or vaccination (as indicated), blue represents lower production. Asterisks in discovery cohort: FDR adj. p <0.05 in discovery cohort and p <0.05 in validation with same directionality: Results from ANCOVA on rank transformed data, adjusted for seasonality.

PHA: phytohemagglutinin; MTB: M. Tuberculosis; C.Alb.con: Candida albicans. C.alb.hy: C. Albicans hyphae. S. Pneu: S. Pneumoniae

*: FDR adj. p < 0.05 in discovery cohort and p < 0.05 in validation cohort

**Vaccination- Discovery Vaccination - Validation**

**COVID19- Discovery COVID-19 - Validation**

M.


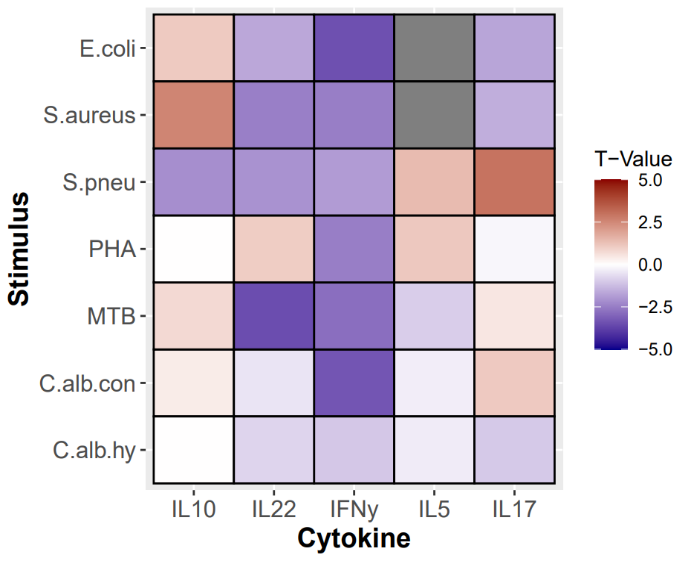

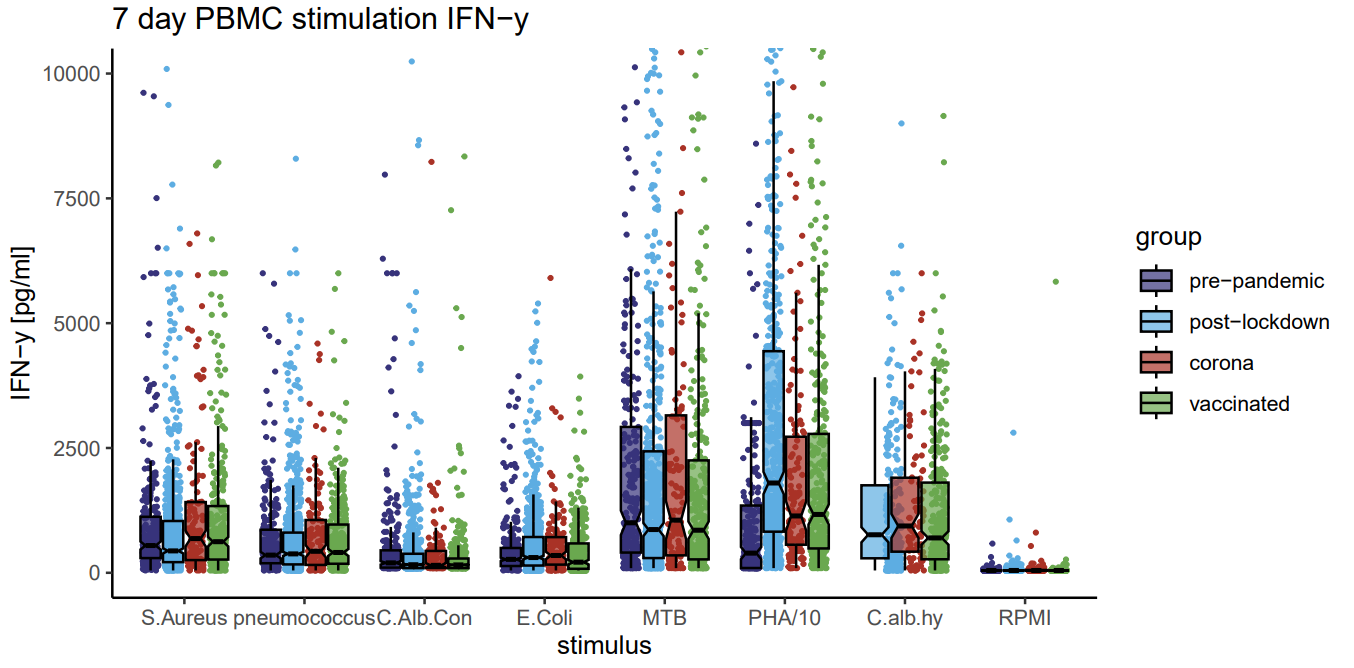


N. QQ plot ANCOVA before and after rank transformation

24h IL-1b – LPS RAW vs transformed


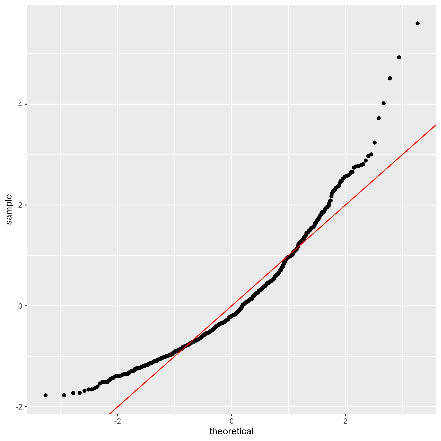

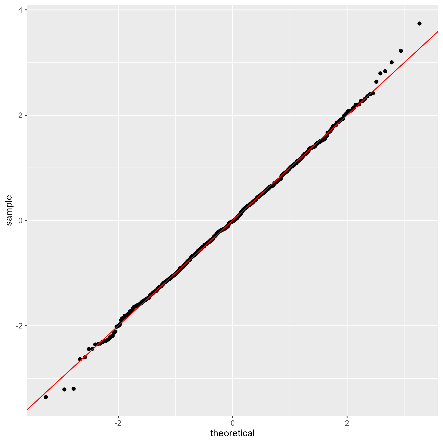


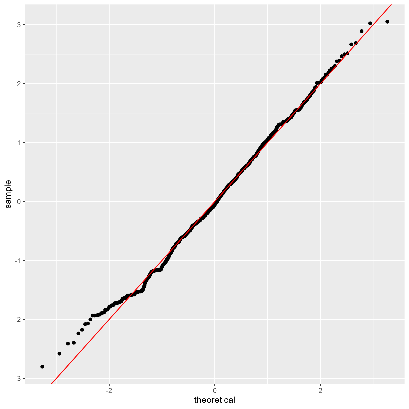

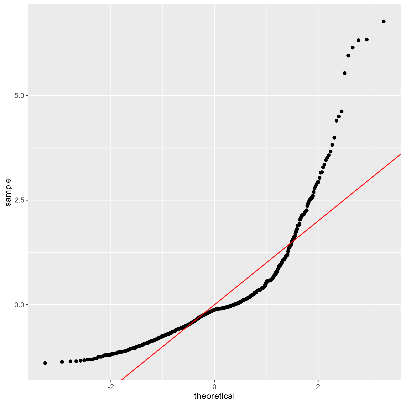
7d IFN-y – PHA RAW vs transformed

Figure S2 M: Box Scatter Plot showing raw data IFN-y concentration in supernatants per group after 7 day ex-vivo stimulation per group. Y-axis: IFN-y concentration. X-axis stimuli. The IFN-y concentration after PHA is divided by 10 to fit on graph.

Figure S2 N: QQ plots before (RAW) and after rank transformation (transformed) for IL-1b production after 24 hours stimulation with LPS (upper graphs) and IFN-y after 7 day stimulation with PHA (lower graphs). Data points represent the observed quantiles of cytokine production, while the expected quantiles under the assumption of a normal distribution are represented by the reference line. Deviations from the reference line indicate departures from normality. Graphs serve as examples for effect and necessity for non-parametric transformation.


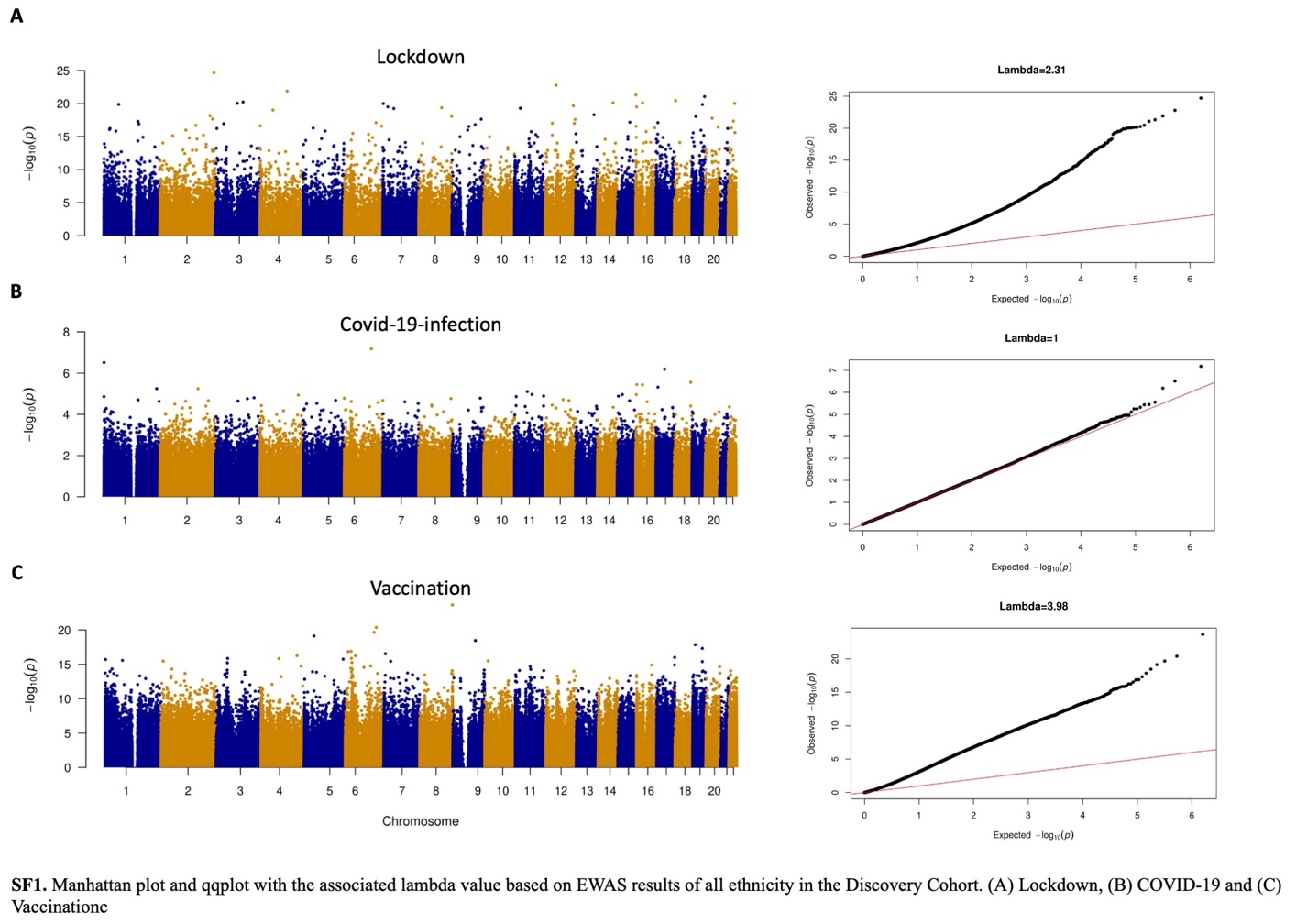


Figure S3. Manhattan plot and the QQ plot of EWAS study of investigating the effect of lockdown, COVID-19 infection and vaccination. (A) Lockdown (pre-lockdown, post-lockdown), (B) COVID-19 infection (infected and non-infected) and (C) Vaccination (vaccinated and non-vaccinated). The EWAS analysis includes individuals of all ethnicities from the Discovery cohort. The -log10(p-value) of the CpG sites is employed for plotting the results.


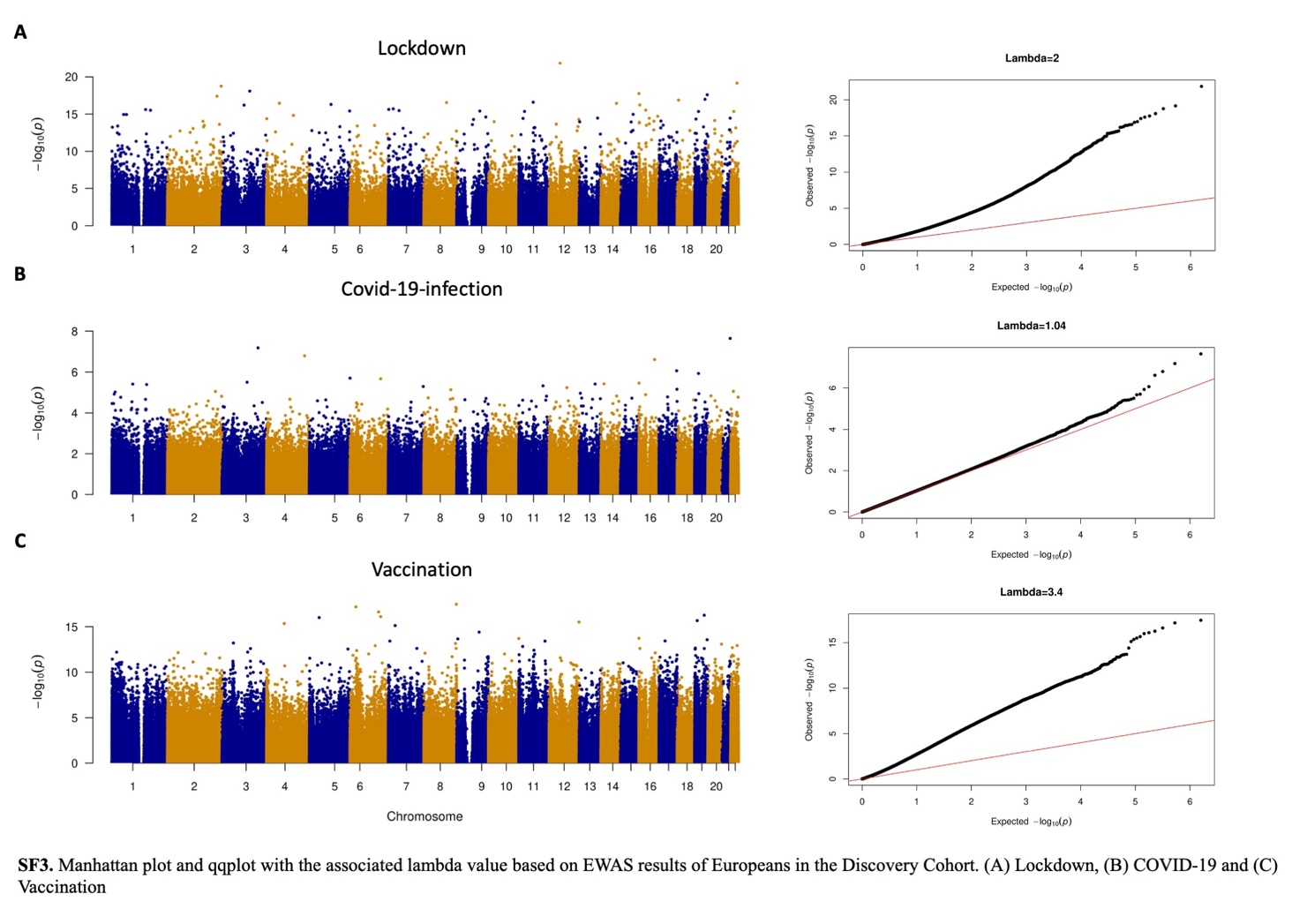


Figure S4. Manhattan plot and the QQ plot of EWAS study of investigating the effect of lockdown, COVID-19 infection and vaccination (A) Lockdown (pre-lockdown, post-lockdown), (B) COVID-19 infection (infected and non-infected) and (C) Vaccination (vaccinated and non-vaccinated). The EWAS analysis includes individuals of the European population from the Discovery cohort. The -log10(p-value) of the CpG sites is employed for plotting the results.


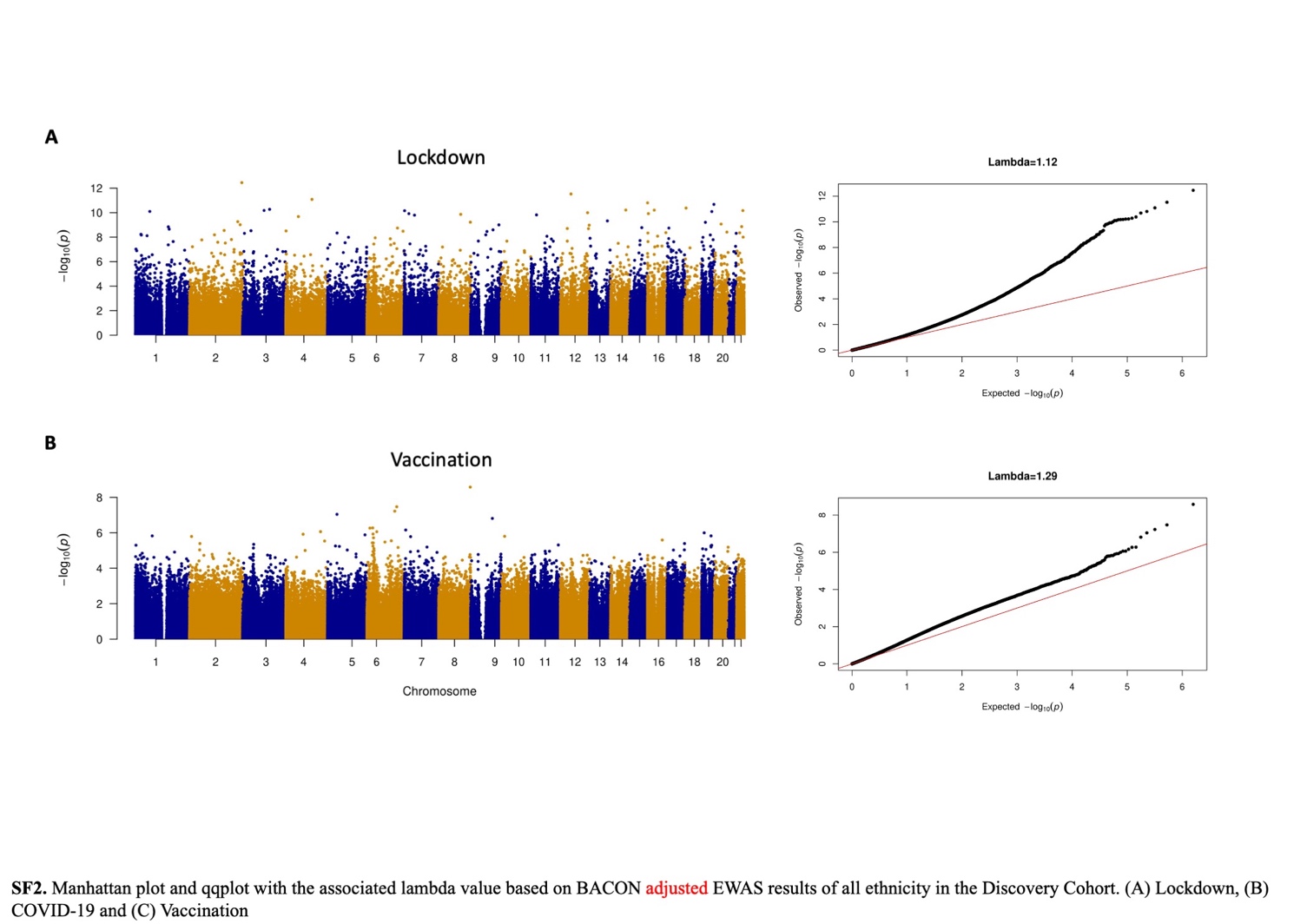


Figure S5. Manhattan plot and the QQ plot of EWAS study of investigating the effect of lockdown and vaccination after BACON adjustment. (A) Lockdown (pre-lockdown, post-lockdown), (B) Vaccination (vaccinated and non-vaccinated). The EWAS analysis includes individuals of all ethnicity from the Discovery cohort. The -log10(p-value) of the CpG sites is used for the plotting.


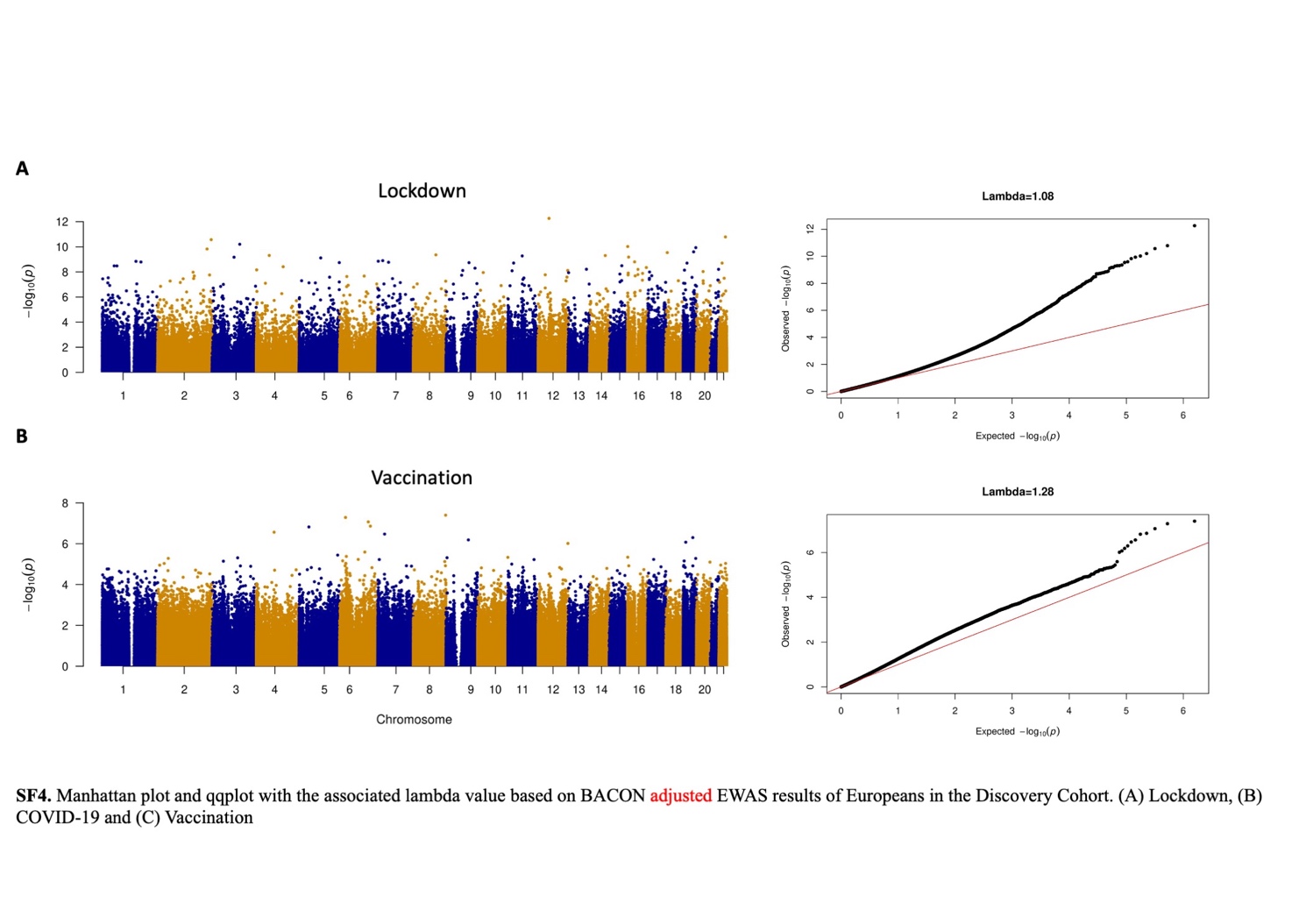


Figure S6. Manhattan plot and the QQ plot of EWAS study of investigating the effect of lockdown, COVID-19 vaccination after BACON adjustment. (A) Lockdown (pre-lockdown, post-lockdown), (B) Vaccination (vaccinated and non-vaccinated). The EWAS analysis includes individuals of the European population from the Discovery cohort. The -log10(p-value) of the CpG sites is employed for plotting the results.


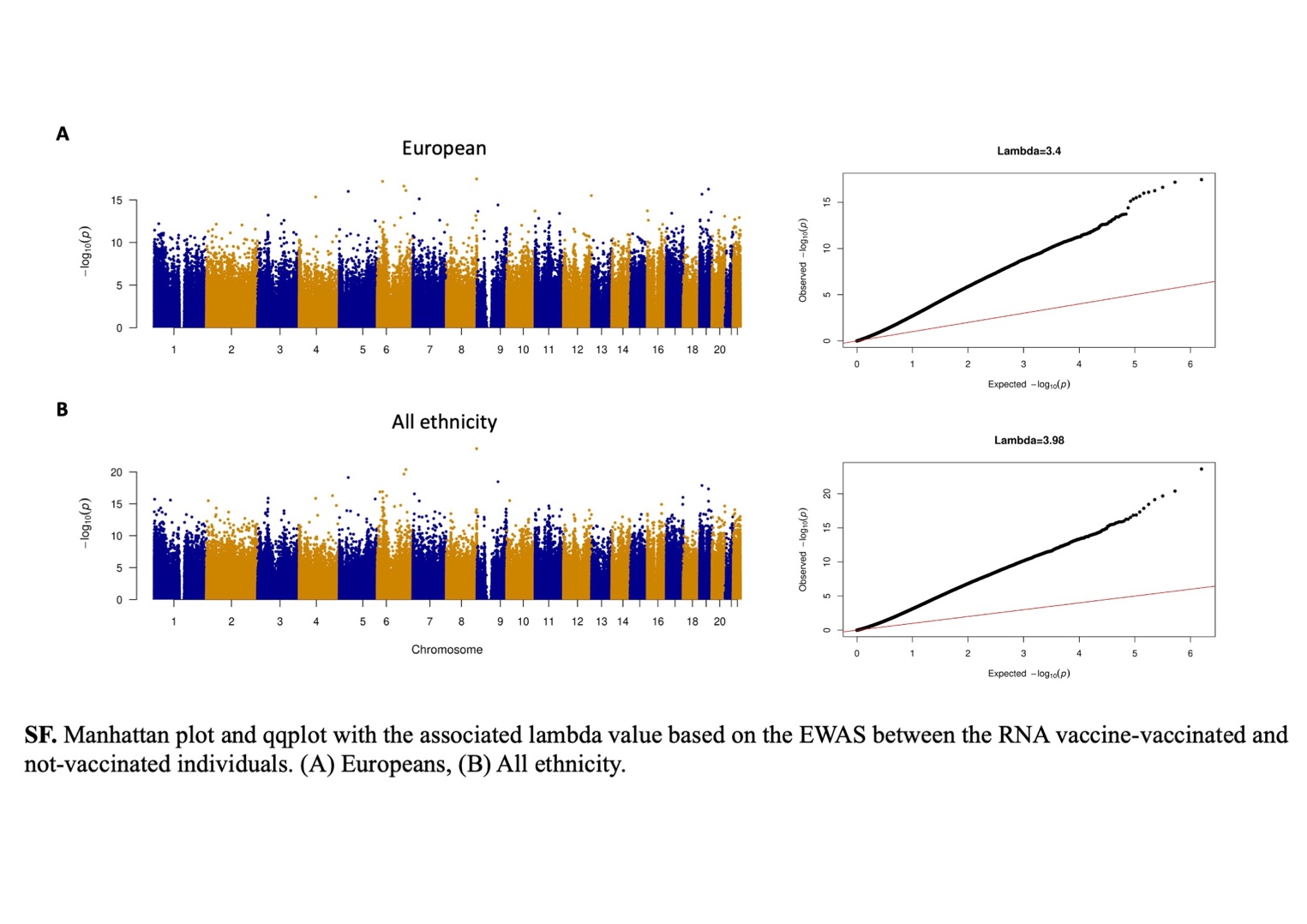


Figure S7. Manhattan plot and QQ plot of the EWAS study investigating the effect of mRNA vaccine on CpG sites in vaccinated and non-vaccinated individuals. (A) presents the EWAS analysis conducted with the European population in the Discovery cohort, (B) includes individuals of all ethnicities within the Discovery cohort. The -log10(p-value) of the CpG sites is employed for the plotting of results.


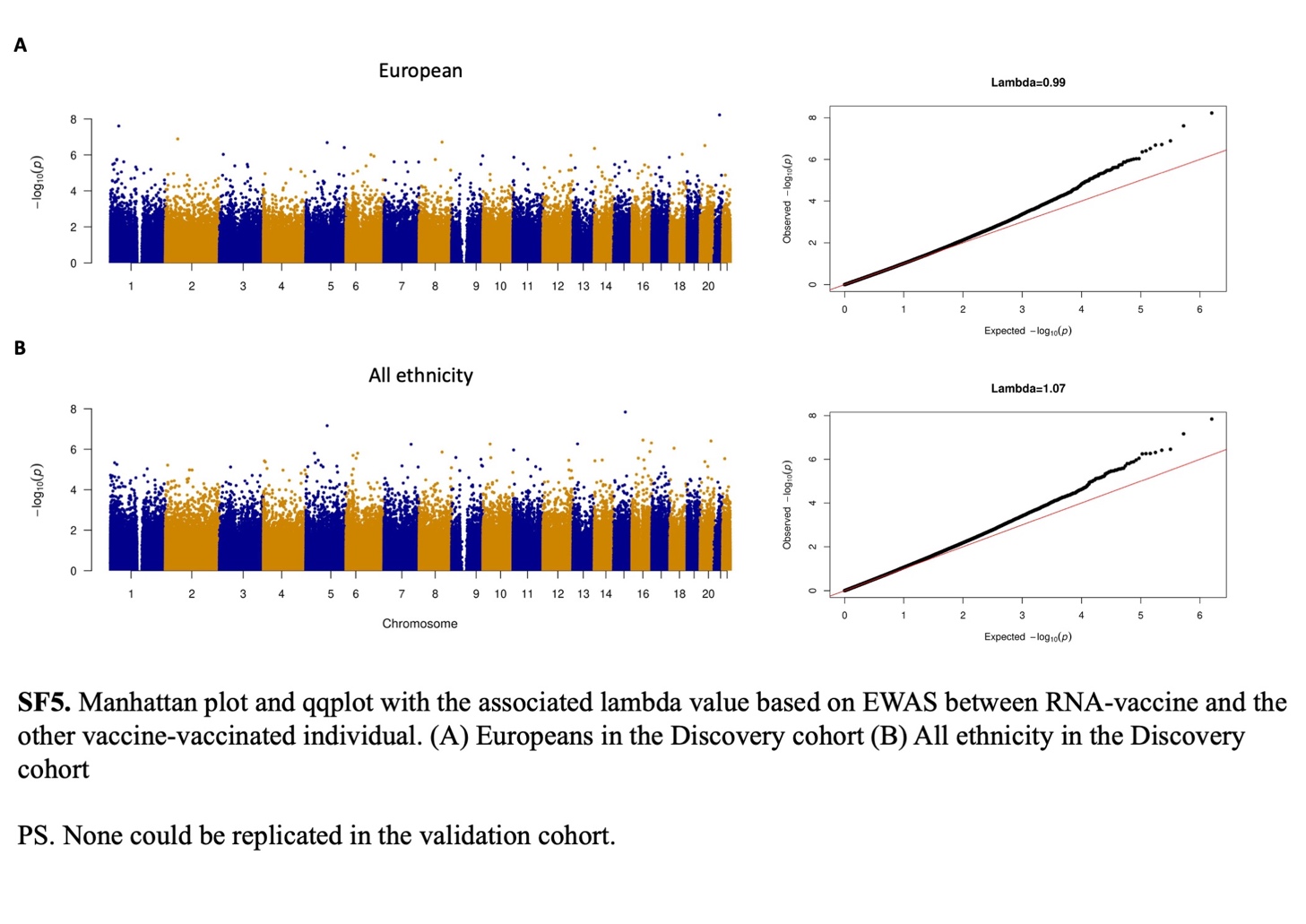


Figure S8. Manhattan plot and QQ plot of the EWAS study investgating the effect of mRNA vaccine and viral vector vaccines. (A) The EWAS analysis includes individuals of the European population from the Discovery cohort., (B) The EWAS analysis includes individuals of all ethnicities from the Discovery cohort. -log10(p-value) of the CpG sites is used for the plotting.


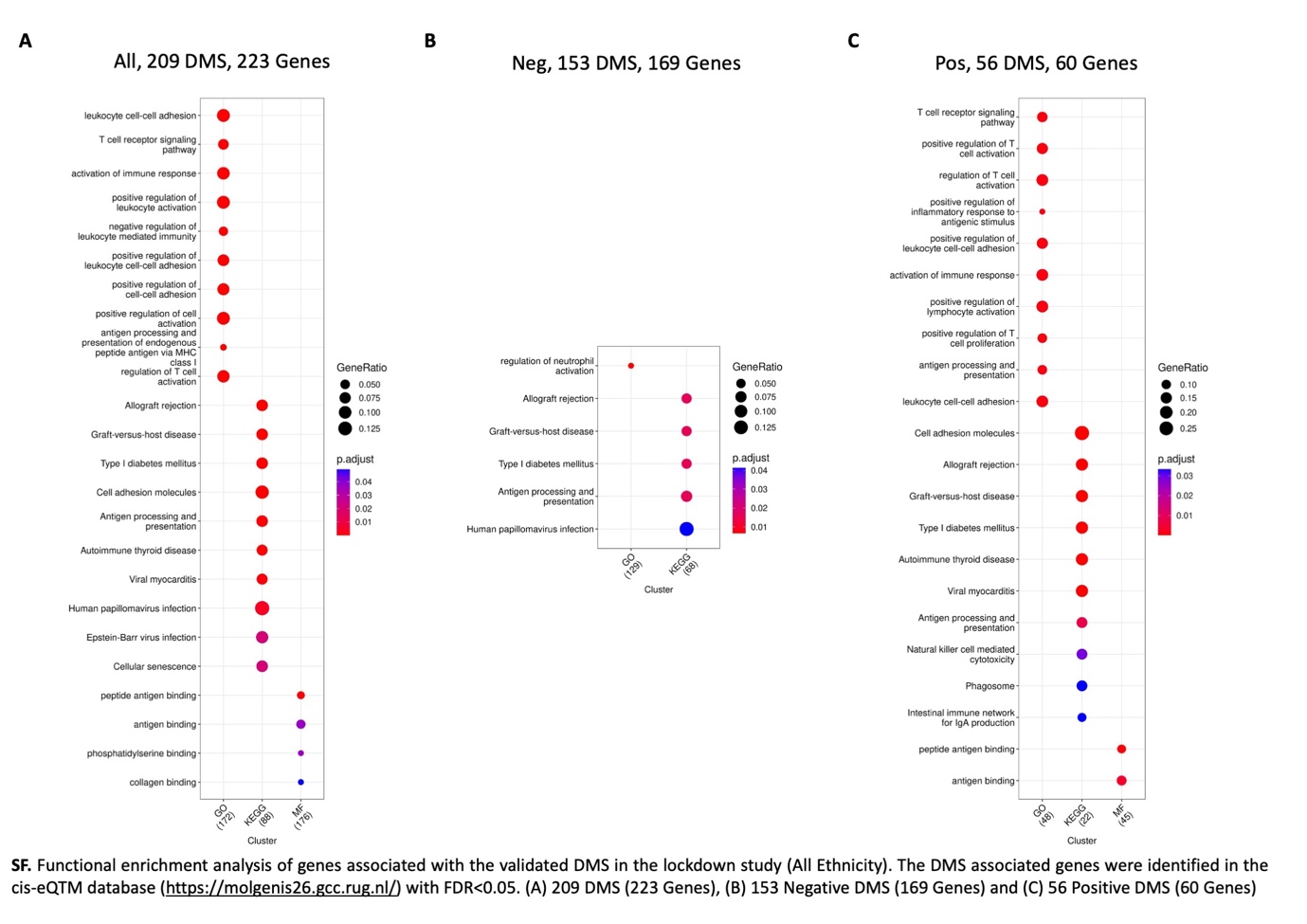


Figure S9. Functional enrichment analysis of genes associated with the validated differentially methylated sites (DMS) in the lockdown study (all ethnicity). The DMS associated genes were identified in the *cis*-eQTM database (<https://molgenis26.gcc.rug.nl/>) with FDR<0.05. (A) 209 DMS showed in the *cis*-eQTM database with 223 associated Genes, (B) 153 de-methylated (Neg) DMS due to lockdown with 169 associated Genes and (C) 56 more methylated DMS (Pos) due to lockdown with associated with 60 Genes.


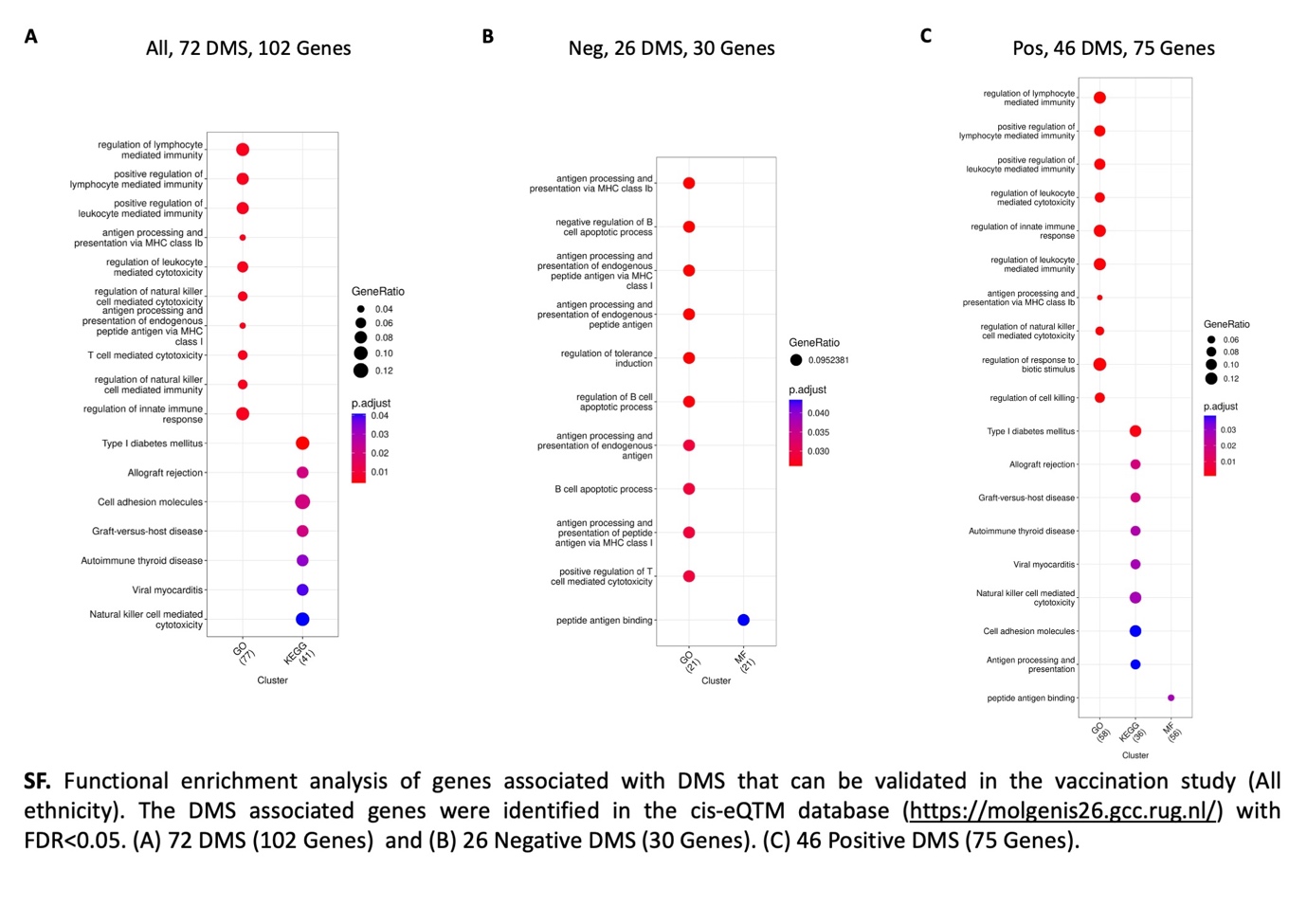


Figure S10. Functional enrichment analysis of genes associated with the validated DMS in the vaccination study (all ethnicity). The DMS associated genes were identified in the *cis*-eQTM database (<https://molgenis26.gcc.rug.nl/>) with FDR<0.05. (A) 72 DMS showed in the *cis*-eQTM database 102 associated Genes, (B) 26 de-methylated (Neg) DMS due to vaccination with 30 associated Genes and (C) 46 more methylated DMS (Pos) associated with vaccination, 75 associated Genes were found.


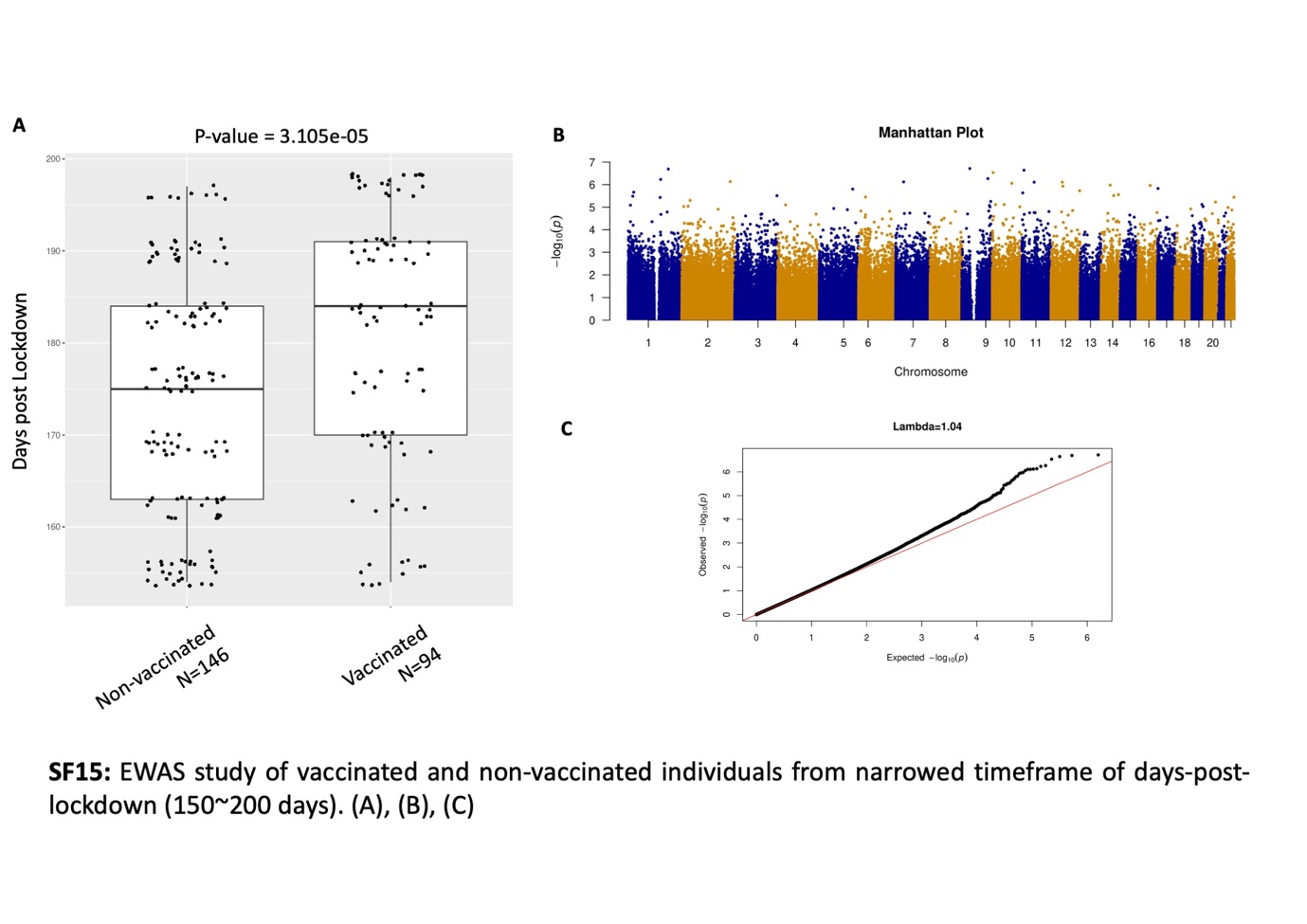


Figure S11. EWAS study comparing vaccinated and non-vaccinated individuals within a narrowed timeframe of days-post-lockdown (150~200 days). (A) Comparison of days-post-lockdown between the vaccinated and non-vaccinated groups. Wilcoxon test was used to detect the difference between the two groups. (B) Manhattan plot for CpG sites associated with vaccinated and non-vaccinated. (C) QQ plot displaying the distribution of CpG sites in (B). -log10(p-value) of the CpG sites is used for the plotting.

**Figure S12 Olink panel (proteome) bridging process**

A.
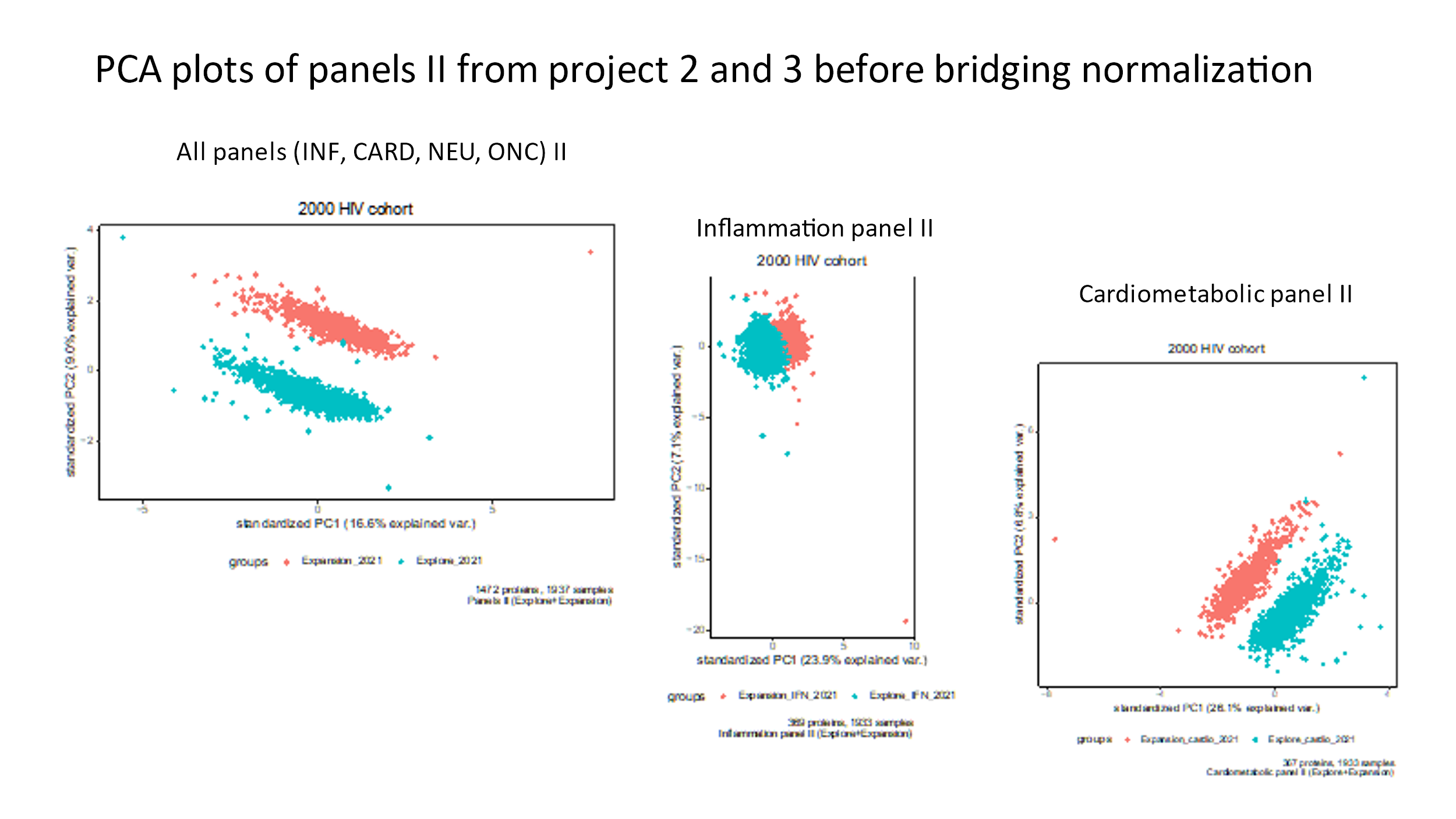


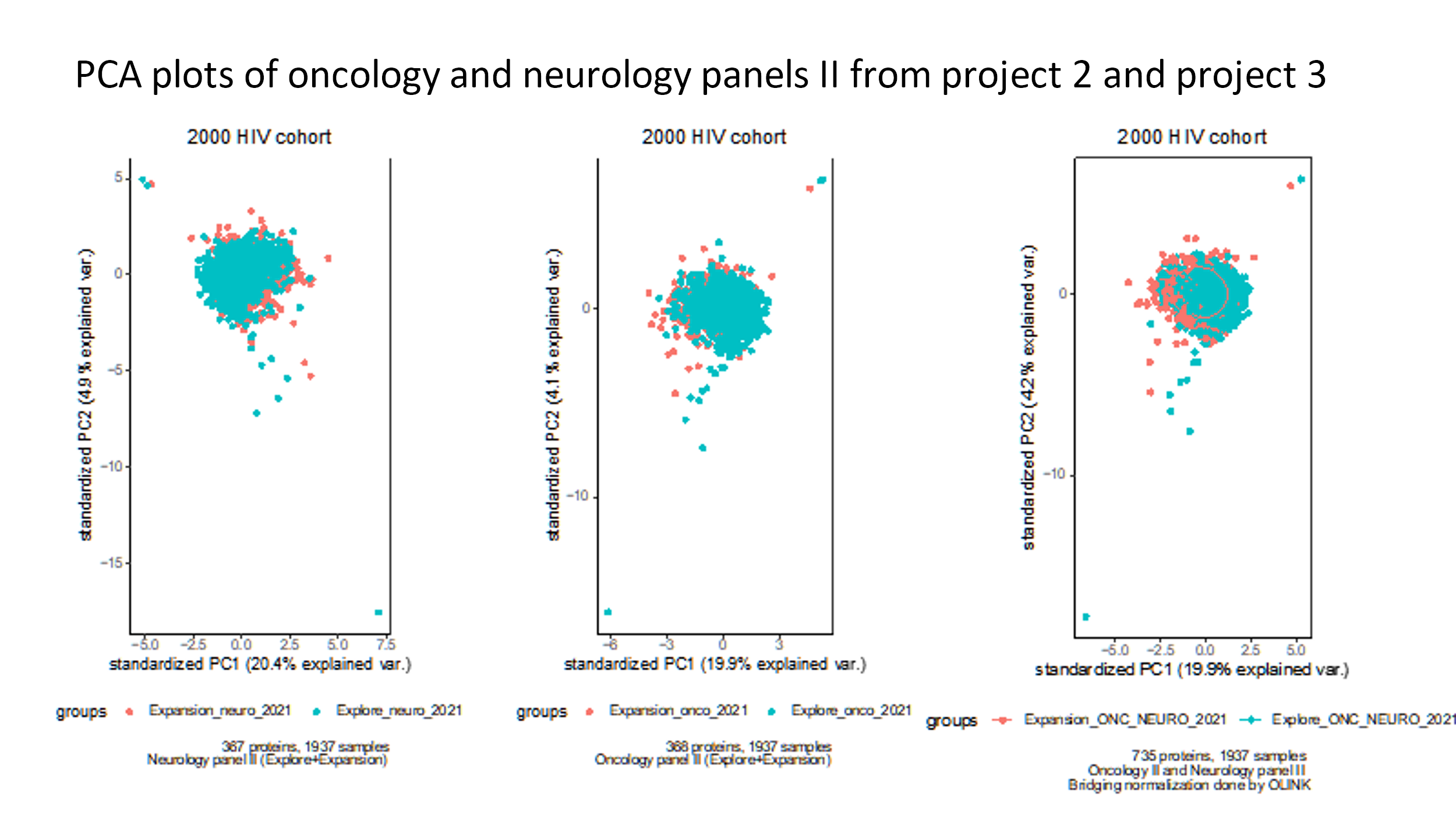


Plasma samples from PLHIV were measured in three batches. The first batch (n = 692 samples) was measured using the library Olink® Explore 1536 consisting of 1472 proteins divided into four 384-plex panels focused on inflammation, oncology, cardiometabolic and neurology proteins (panels I). The second batch (n = 692 samples) was measured using the Olink® Explore Expansion 1536 consisting of 1472 proteins divided into four 384-plex panels focused on additional inflammation II, oncology II, cardiometabolic II and neurology II proteins (panels II). The third batch was measured using the full library (Olink® Explore 3072) consisting of ~3000 proteins divided into eight 384-plex panels focused on inflammation, oncology, cardiometabolic and neurology proteins (panels I and II). Protein measurements are delivered as Normalized Protein expression (NPX) values, which is Olink’s relative protein quantification unit on log2 scale. Olink has developed a built-in quality control (QC) system using internal controls to control over technical performance of assays and samples. In addition, Olink services performed bridging normalization between oncology panel II and neurology panel II from batch two and three using 27 bridging samples. In addition to Olink’s QC, we performed bridging normalization to remove batch effect between inflammation, oncology, cardiometabolic and neurology panels from batch one and three using 32 bridging samples. In addition, we performed bridging normalization between cardiometabolic panel II and inflammation panel II from batch two and three using 23 bridging samples. Bridging normalization was performed by following the next steps for each protein: (1) We first calculated the median of the bridging samples for each protein in the two batches. (2) Then, we calculated the median difference keeping one batch as a reference. (3) We finally subtracted the median difference from each protein in the non-reference batch. For bridging normalization between batch one and three, we used 1461 unique proteins measured in 692 samples that were common in batch one and three. For normalization between cardiometabolic II and inflammation II panel from batch two and three, we used 736 proteins measured in 1268 samples. PCA showed no batch effect after bridging normalization (example is shown in this figure). Limit of detection (LOD) values per protein were re-adjusted by the same adjustment factor as the respective protein measurements after bridging normalization.

B. Flow diagram


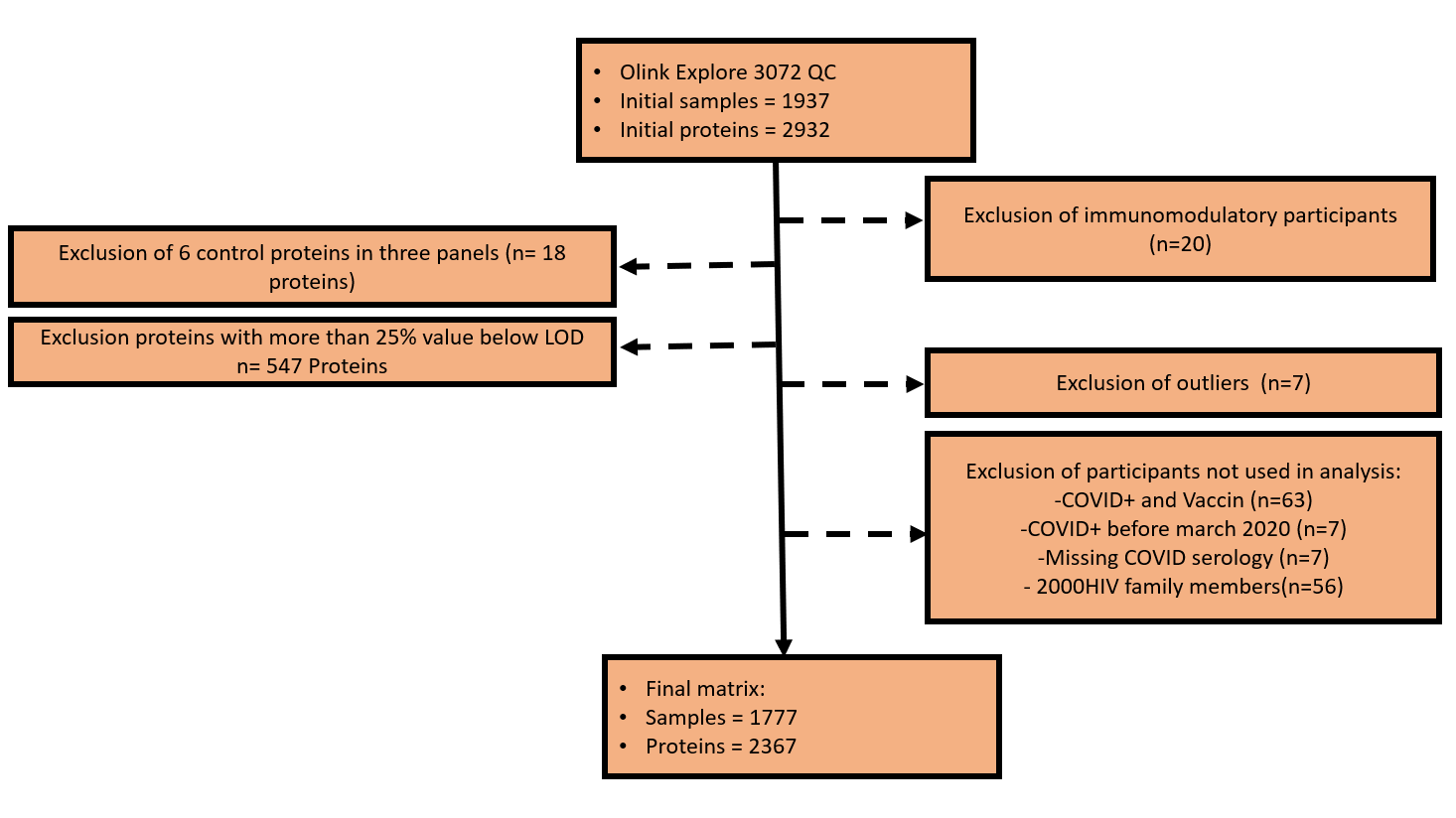

Supplement: Supplementary file 1 [file DataSheet1.docx]
